# Supplementary material for: Deconvolution of Buparlisib's mechanism of action defines specific PI3K and tubulin inhibitors for therapeutic intervention
Source: Nat Commun. 2017 Mar 9;8:14683. doi: 10.1038/ncomms14683 (PMC5347140; doi:10.1038/ncomms14683)
Supplement: Supplementary Information — Supplementary Figures, Supplementary Tables, Supplementary Methods and Supplementary References [file ncomms14683-s1.pdf]

SUPPLEMENTARY FIGURES

Supplementary Fig. 1

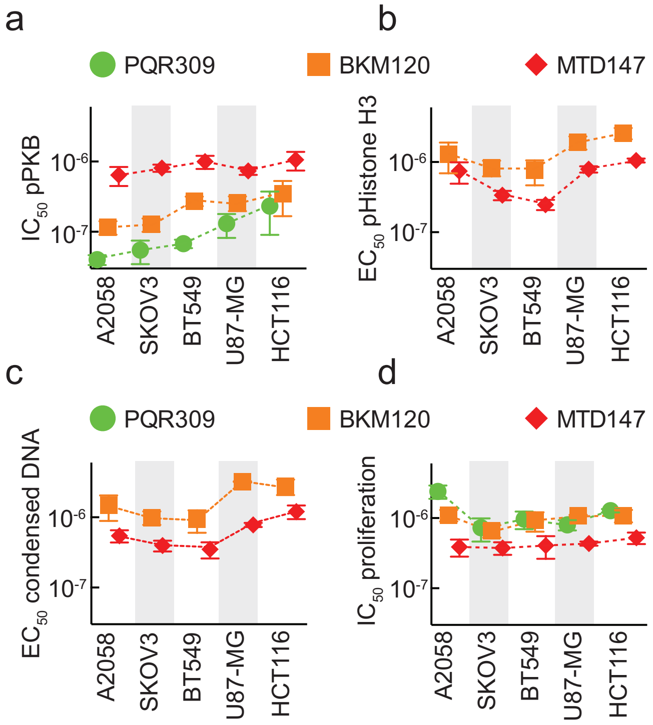

Supplementary Fig. 1

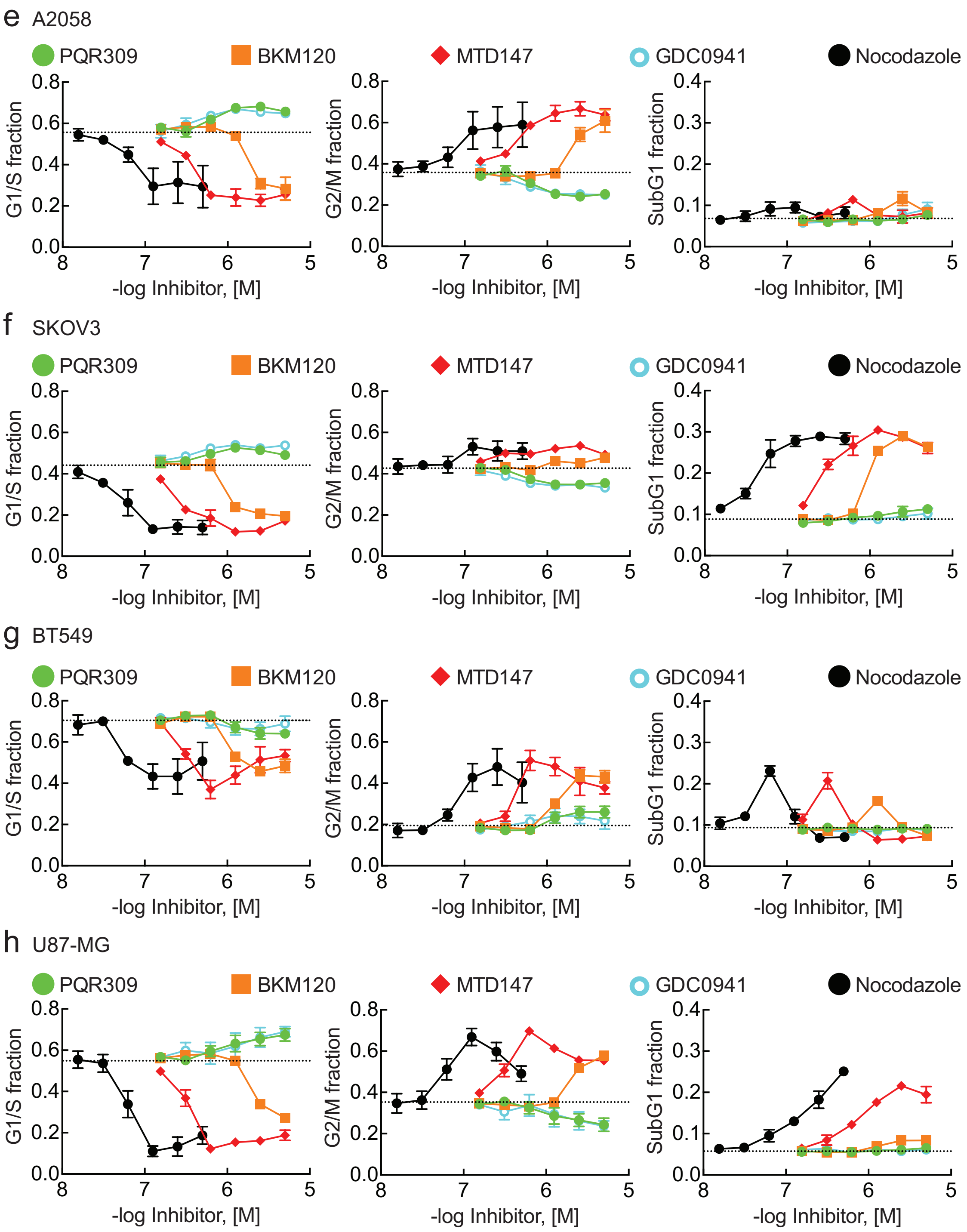

Supplementary Fig. 1

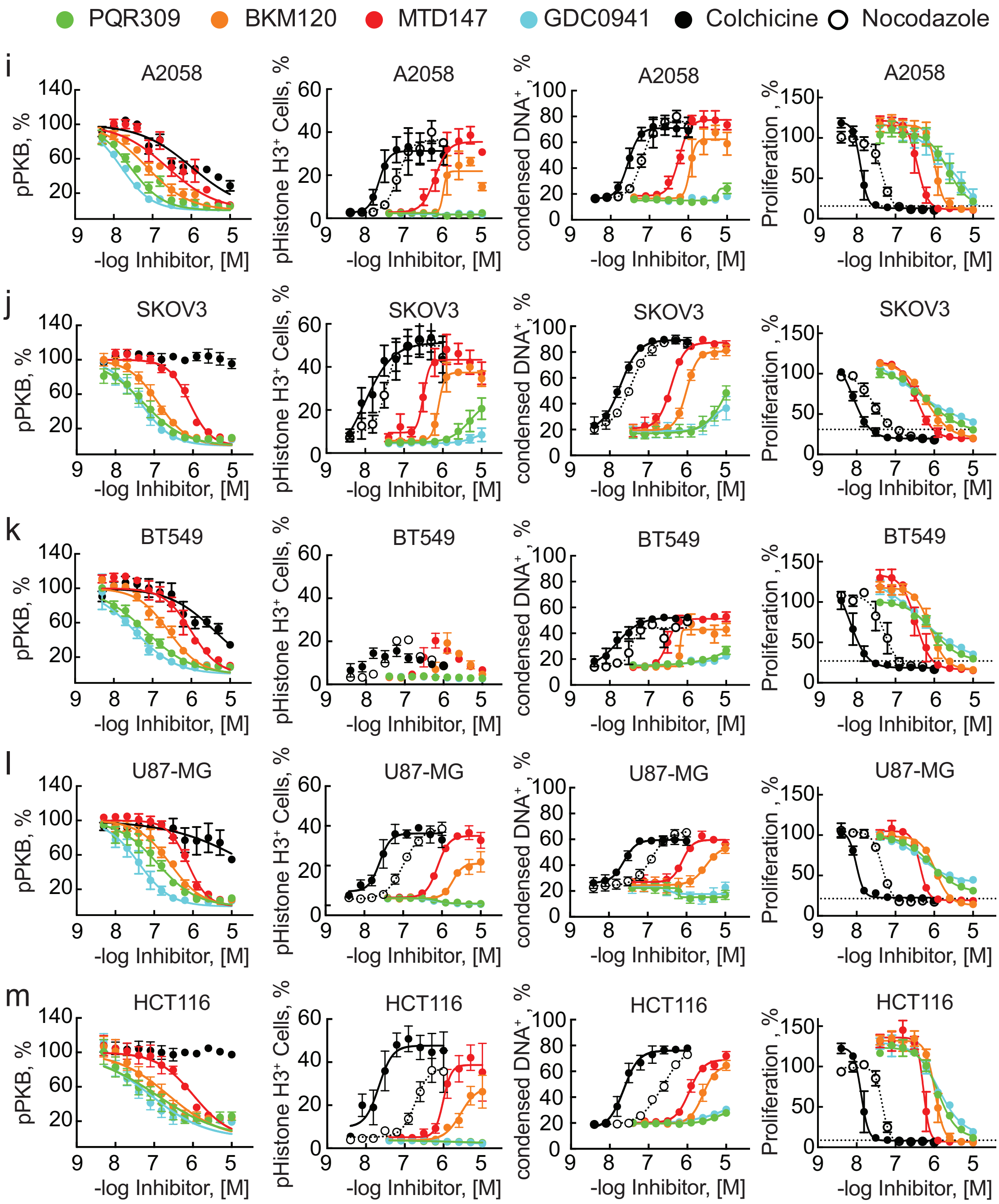

Supplementary Fig. 1

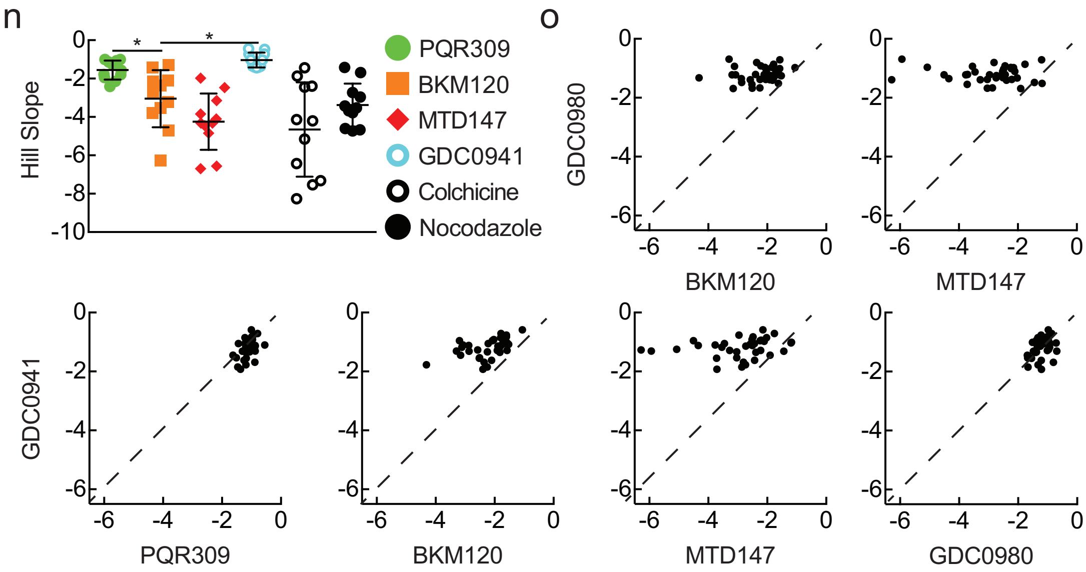

Supplementary Fig. 1

p

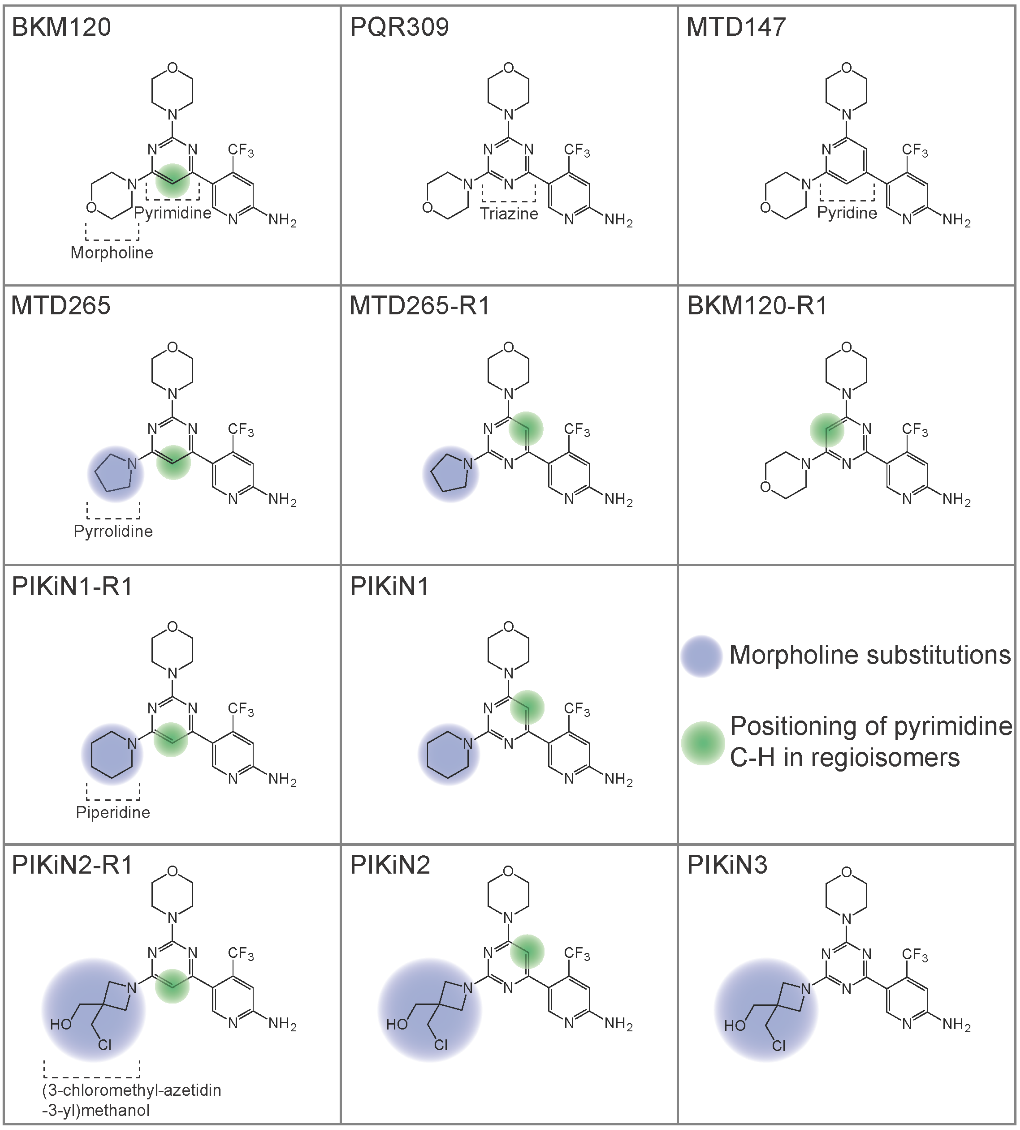

Reference compounds

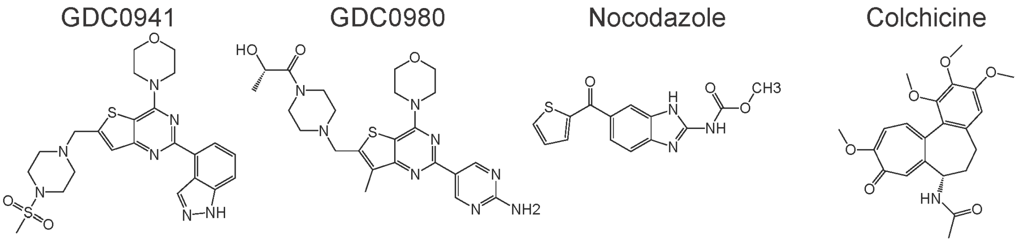

Supplementary Fig. 1 Depicted cell lines were exposed to PQR309 (•) BKM120 (■) and MTD147 (◆), and (**a**) the IC_{50} for PKB/Akt phosphorylation (n=3-5, mean ± SEM), (**b**) the EC_{50} of phospho-Histone H3 positive cells (n=3, BT-549 n=4, mean ± SEM), (**c**) the EC_{50} of appearance of cells with condensed nuclear DNA (n=3, BT-549 n=4, mean ± SEM) and (**d**) IC_{50} for proliferation was determined (n=3, mean ± SEM). (**e-h**) Cell cycle distribution of indicated cells as function of drug concentration after 24 h drug exposure; PQR309 (•) BKM120 (■) and MTD147 (◆), GDC0941 (○) and nocodazole (•). Cells were classified into G1/S, G2/M and sub G1 phases. Cell numbers are expressed as % of total cells (n=4; mean ±SEM, dashed line indicates mean of DMSO). (**i-m**) Dose response curves of PQR309, BKM120 and MTD147 – including the reference drugs GDC0941 (PI3K inhibitor) and colchicine or nocodazole (microtubule disrupting agents) for the phosphorylation of PKB (% of DMSO control), the percentage of phospho-Histone H3 positive cells, the percentage of cells with condensed DNA and cell proliferation (as % of DMSO control) in the indicated cell lines (n≥3 as in Supplementary Fig. 1a-d, data points: mean ± SEM). Curves were used for IC_{50}, EC_{50} determinations (Supplementary Fig. 1a-d) and hill slope values of growth inhibition (Supplementary Fig. 1n) (**n**) Quantification of hill slope steepness of non-linear regression curves with variable slope fitted to cell proliferation for the indicated drugs on 5 cell lines (n=2 per cell line, n=3 for U87-MG, * $p < 0.05$; one-way ANOVA, Tukey's multiple comparisons test). BKM120 was not statistically significant different from MTD147, colchicine and nocodazole. (**o**) Additional correlation blots of hill slope steepness. Dashed black line indicates correlation =1.0. (**p**) Overview of chemical structures used in this study.

Supplementary Fig. 2

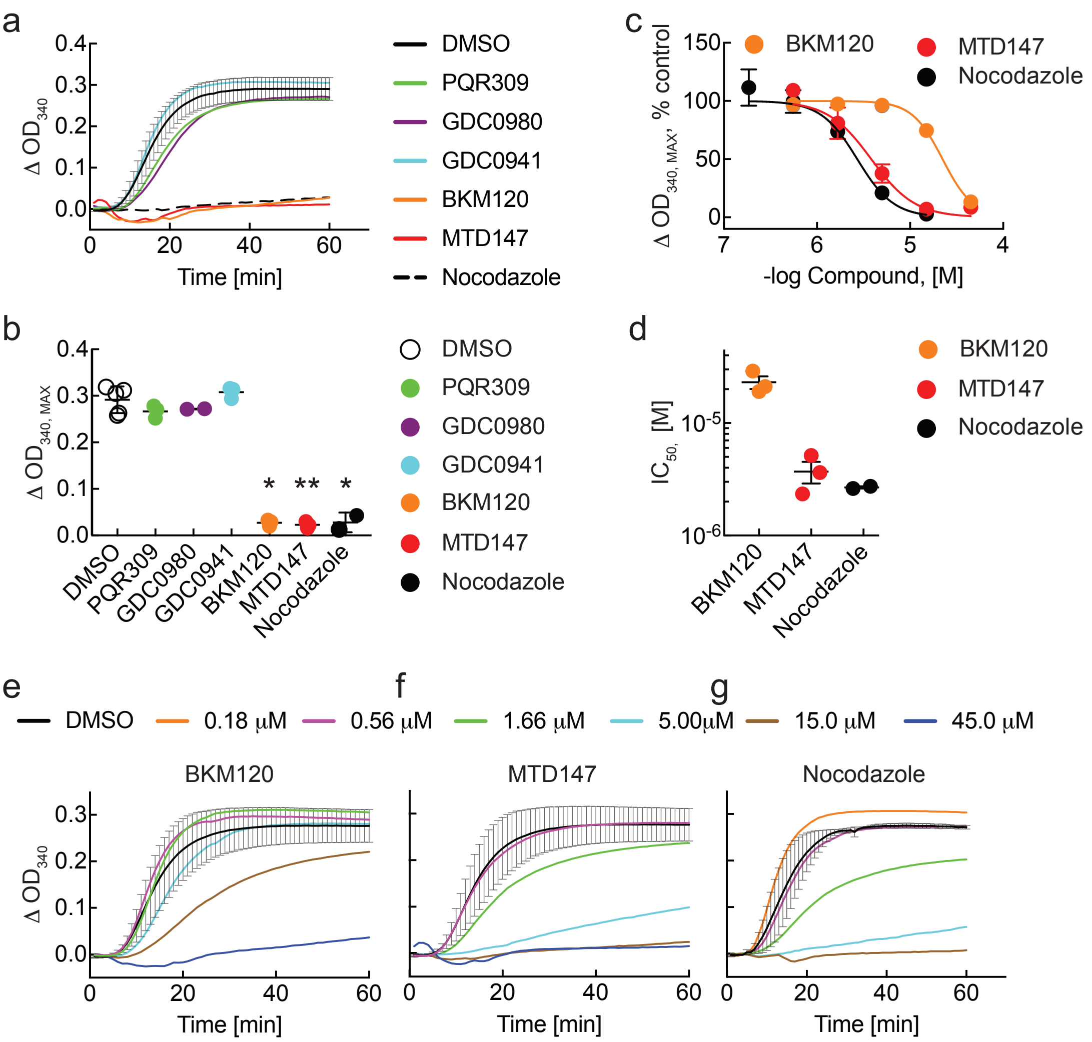

Supplementary Fig. S2 Inhibition of *in vitro* tubulin polymerization. (**a**) Tubulin polymerization was monitored for 60 min. in presence of indicated compounds (45 µM; for nocodazole 10 µM) as change of absorbance at 340 nM (DMSO n= 5 mean± SD; PQR309, BKM120, MTD147, GDC0941 n=3 (mean), GDC0980, Nocodazole n=2 (mean); standard errors for compounds have been omitted for readability). (**b**) Quantification of the maximal tubulin polymerization ($\Delta OD_{340,\ MAX}$) of experiments in (a) (* $p<0.05$, Kruskal-Wallis test). (**c**) Dose response curves of maximal tubulin polymerization (normalized to DMSO controls) in presence of indicated compounds (mean± SEM, BKM120 and MTD147 n=2-4 per concentration from 6 or 7 independent experiments, nocodazole n=2). (**d**) Quantification of half maximal concentrations for inhibition (IC_{50}) of tubulin polymerization of indicated compounds (n=3 for BKM120 and MTD147, n=2 for nocodazole; error bars SEM). Values of more IC_{50}s are summarized in Supplementary Table 4 (**e-g**) Time dependent tubulin polymerization curves in presence of indicated compound concentrations for BKM120 (**e**, DMSO n=8, mean± SD, n= 2-4 for each BKM120 concentration), (**f**) for MTD147 (DMSO n=8 (identical as in (e), mean± SD, n= 2-4 for each MTD147 concentration) and (**g**)

Nocodazole (control and nocodazole concentrations n=2). Error bars for drugs have been omitted for readability.

Supplementary Fig. 3

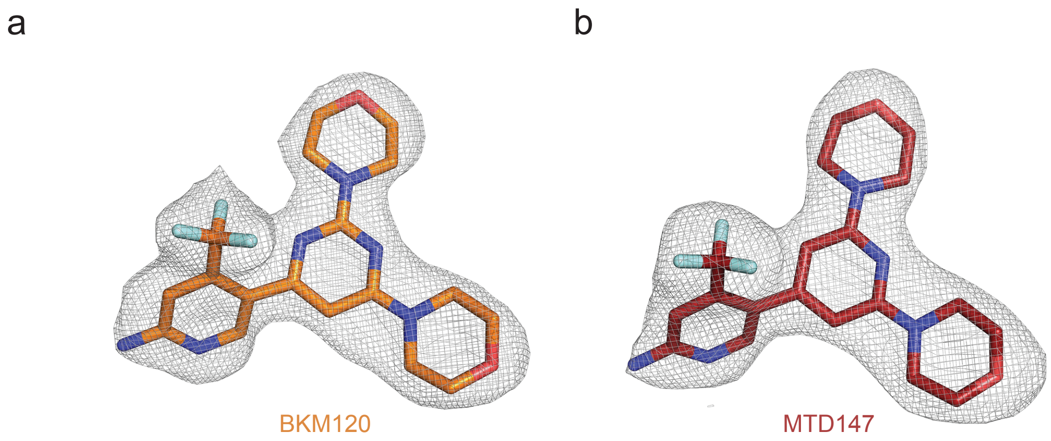

Supplementary Fig. 3 SigmaA-weighted mFo-DFc omit maps (grey mesh) for tubulin-bound BKM120 (**a**) and MTD147 (**b**) are contoured at + 3.0σ.

Supplementary Fig. 4

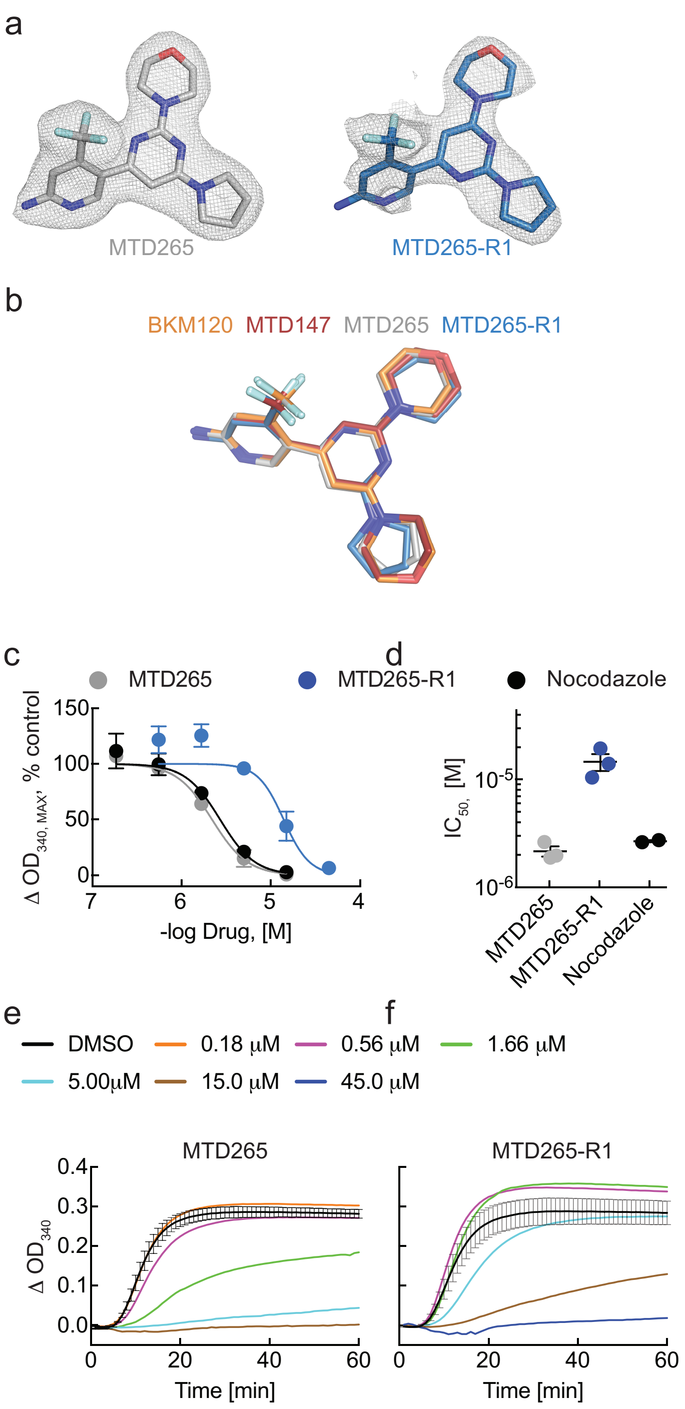

Supplementary Fig. 4

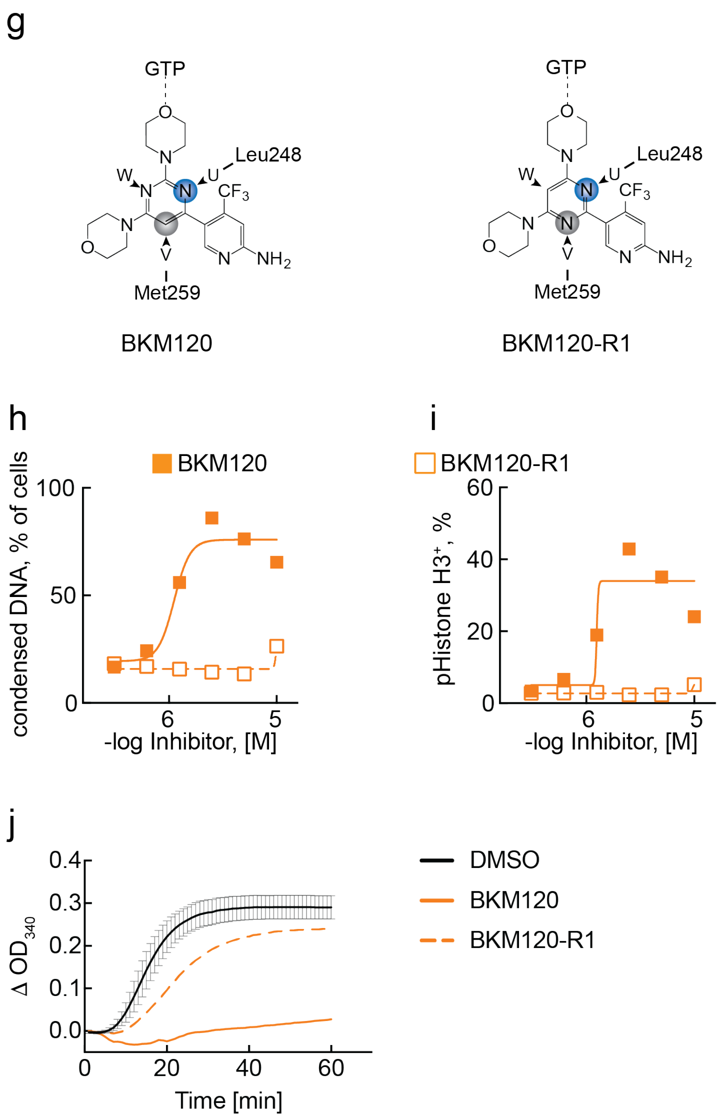

Supplementary Fig. 4 (**a**) SigmaA-weighted mFo-DFc omit maps (grey mesh) for tubulin-bound MTD265 and MTD265-R1 are contoured at + 3.0σ. (**b**) Overlay of BKM120, MTD147, MTD265 and MTD265-R1 orientations bound to the colchicine-binding pocket. (**c**) Dose response of maximal tubulin polymerization (normalized to DMSO controls) in presence of indicated compounds (n=3; mean± SEM, for MTD265 and MTD265-R1; nocodazole n=2, mean ± SEM. Nocodazole curves as shown in Supplementary Figure 2). (**d**) IC_{50}s for tubulin polymerization of indicated compounds (n=3 for MTD265 and MTD265-R1, n=2 for nocodazole; mean ± SEM). (**e-f**) Time

dependent tubulin polymerization curves in presence of indicated compound concentrations for MTD265 (**e,** n=3 [15 μM: n=2]; mean (± SD shown for DMSO only)) or MTD265-R1 (**f**, n=3; mean (± SD shown for DMSO only)). (**g**) Chemical structure of BKM120 and of its regioisomer BKM120-R1. GTP, Met259 and Leu248 indicate the orientation of BKM120 and a putative placement of BKM120-R1 in tubulin. (**h, i**) Effect of BKM120 and BKM120-R1 on cells positive for DNA condensation, or phospho-Histone H3 (both as percentage of total cells; n=2x2, mean ±SEM). (**j**) Time-dependent tubulin polymerization curves in presence of BKM120 and its regioisomer BKM120-R1 at 45 μM (DMSO n=5; mean±SD, BKM120 n=3; mean, BKM120-R1 n=2; mean; DMSO and BKM120 curves as in Supplementary Figure 2a, added here for comparison with BKM120-R1).

Supplementary Fig. 5

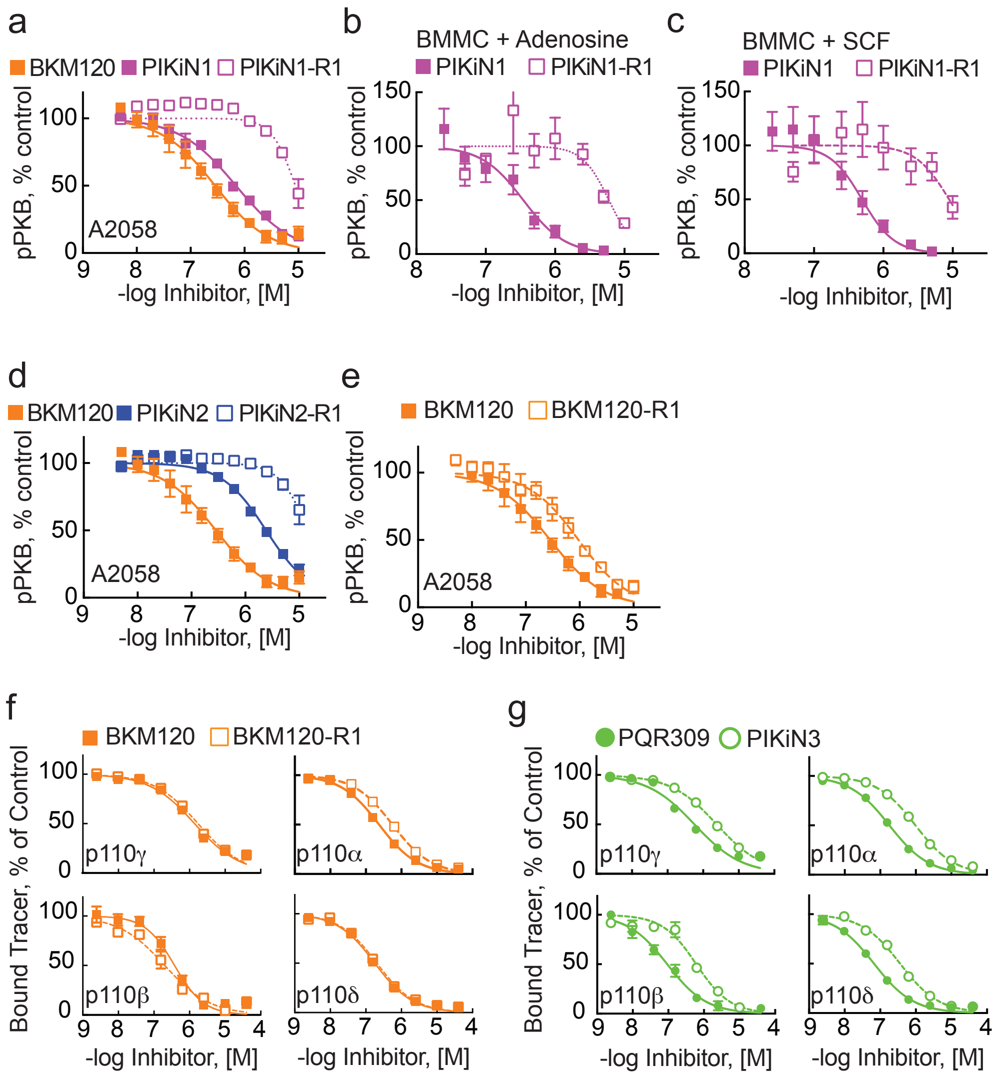

Supplementary Fig. 5 (**a**) Inhibition of PKB phosphorylation in A2058 cells was determined and is given as % of DMSO control. Cells were exposed for 1h to the indicated compounds and concentrations, followed by detection of pSer473 PKB using in cell western technology (n= 4, mean ± SEM). (**b, c**) Inhibition of bone marrow derived mast cell (BMMC) signaling by PIKiN1 and PIKiN1-R1. Post-starvation and inhibitor treatment BMMC were stimulated with 2 µM Adenosine (PI3Kγ specific signaling, **b**) or 10 ng/ml SCF (PI3Kδ driven, **c**). Inhibition of PI3K signaling was monitored measuring the phosphorylation of PKB by western blot analysis. Signals were quantified using ImageJ and plotted with GraphPad Prism including non-linear regression fitting (n=4 in **b**, n=5 in **c**), mean ± SEM, given as % of DMSO control). (**d, e**) Inhibition of Ser473 phosphorylation of PKB in A2058 cells upon 1h exposure to the indicated compounds (n= 4, mean ± SEM). BKM120 in (a, d, e) are from identical

experiments. (**f** and **g**) *In vitro* binding of BKM120 and its regioisomer BKM120-R1 (**h**) and PQR309 and its derivative PIKiN3 (**i**) to the catalytic pocket of recombinant p110γ, p110α/p85α, p110β/p85α or p110δ/p85α complexes as described in Fig. 3b and d (n=2x2; for p110α n=3x2; mean ± SEM). For p110β binding, unspecific background signals were subtracted. BKM120 values for p110γ, p110β and p110δ are from identical experiments as in Fig. 3b and d. PIKiN3 values are from experiments depicted in Fig. 3d.

Supplementary Fig. 6

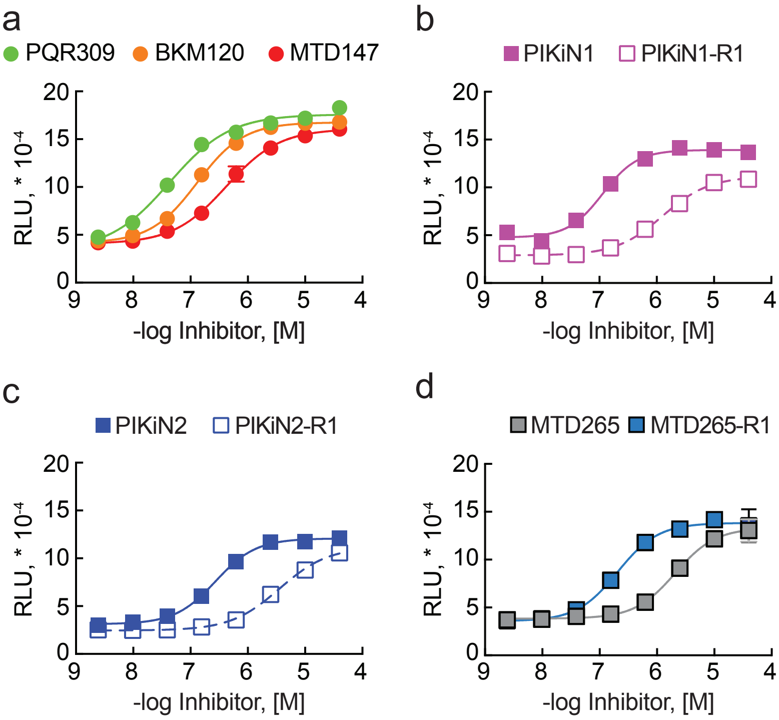

Supplementary Figure 6. Inhibition of PI3Kα activity. PI3Kα (p85α/p110α)-mediated conversion of diC$_8$ PtdIns(4,5)P_2 to diC$_8$ PtdIns(3,4,5)P_3 was measured in presence of ATP and increasing concentrations of the indicated compounds. PI3Kα activity was subsequently determined using AlphaScreen technology. Here, high relative luminescent units (RLU) reflect low PtdIns(3,4,5)P_3 output and low PI3Kα activity. (**a**) Comparison of PQR309, BKM120 and MTD147 (n=2; mean ± SEM; error bars omitted when smaller than symbols). (**b-d**) Comparison of indicated BKM120-derived regioisomer compound pairs (n=2; mean ± SEM, error bars omitted when smaller than symbols). IC$_{50}$ values of all compounds are listed in Supplementary Table 6.

Supplementary Table 1. Drug induced growth inhibition

	Half Maximal Inhibitory Concentration (IC_{50}, [µM])				
**Inhibitor →**<br><br>**Cell Line**	**PQR309**	**GDC0980**	**GDC0941**	**BKM120**	**MTD147**
SHP-77	11.803	2.574	2.879	2.373	0.535
K-562	3.024	0.592	1.529	1.057	0.235
SW480	2.686	0.775	1.274	1.919	0.627
786-0	2.338	0.778	2.108	1.779	0.537
SW620	1.901	0.448	1.204	1.183	0.298
CAL 27	1.586	0.520	1.559	1.198	0.238
Jurkat E6.1	1.526	0.579	1.586	1.028	0.156
BT-549	1.496	0.500	1.201	1.433	0.579
FaDu	1.487	0.457	0.976	2.044	0.553
A-172	1.472	0.344	0.696	1.590	0.554
U-87 MG	1.416	0.629	0.807	1.964	0.756
DLD-1	1.345	0.487	1.347	1.448	0.487
CCRF-CEM	1.333	0.296	1.586	1.392	0.438
A375	1.203	0.426	1.194	1.041	0.213
Hs 578T	1.196	0.301	0.967	1.290	0.414
769-P	1.151	0.368	5.870	1.453	0.368
HCT-116	1.141	0.320	1.105	1.433	0.435
A-427	1.112	0.335	0.765	0.878	0.355
C-33 A	1.102	0.322	0.681	1.843	0.614
NCI-H82	1.086	0.651	0.799	0.822	0.193
SK-N-AS	1.073	0.316	1.442	0.640	0.176
RPMI-7951	1.029	0.465	1.023	1.325	0.444
SJCRH30	1.021	0.299	0.828	1.269	0.600
LS 174T	0.976	0.335	1.311	1.354	0.657
J82	0.966	0.315	0.910	1.419	0.438
PA-1	0.905	0.547	1.520	1.243	0.316
BT-20	0.815	0.354	2.120	1.643	0.675
MeWo	0.784	0.338	1.559	1.423	0.579
BxPC-3	0.695	0.276	0.799	1.064	0.293
MG-63	0.695	0.217	1.107	0.685	0.224
HCT-15	0.674	0.220	1.075	1.015	0.338
A-549	0.635	0.188	0.725	1.260	0.444
U-2 OS	0.615	0.175	0.516	1.207	0.543
A-498	0.588	0.185	1.244	1.097	0.295
NCI-H460	0.494	0.225	0.625	0.982	0.412
MOLT-4	0.486	0.182	0.391	1.034	0.404
SR	0.442	0.249	1.270	0.404	0.150
SW48	0.408	0.172	0.337	1.023	0.342
ACHN	0.400	0.143	0.396	0.861	0.439
AU-565	0.400	0.127	0.392	0.643	0.136
AN3 CA	0.384	0.080	0.353	0.500	0.253
OVCAR-3	0.378	0.105	0.509	0.796	0.323
LoVo	0.337	0.173	1.087	0.877	0.221
A-204	0.122	0.047	0.113	0.539	0.411
Mean of 44	1.289	0.396	1.177	1.215	0.402

Cells were exposed for 72 h to serial dilutions of indicated drugs. Cell viability was subsequently determined by ATPlite 1Step™ detection (PerkinElmer). IC_{50}s were calculated by non-linear regression using IDBS XLfit 5. Cell lines are sorted from low to high sensitivity to PQR309.

Supplementary Table 2. Cell line sensitivity related to cell panel sensitivity

	Log $IC_{50\ cell\ line}$ - Log $IC_{50\ average}$				
**Inhibitor →**<br><br>**Cell Line**	**PQR309**	**GDC0980**	**GDC0941**	**BKM120**	**MTD147**
SHP-77	0.962	0.813	0.389	0.291	0.124
K-562	0.370	0.174	0.114	-0.060	-0.233
SW480	0.319	0.291	0.034	0.198	0.193
786-0	0.259	0.293	0.253	0.166	0.126
SW620	0.169	0.053	0.010	-0.012	-0.130
CAL 27	0.090	0.118	0.122	-0.006	-0.229
Jurkat E6.1	0.073	0.165	0.130	-0.073	-0.412
BT-549	0.065	0.101	0.009	0.072	0.158
FaDu	0.062	0.062	-0.081	0.226	0.138
A-172	0.058	-0.061	-0.228	0.117	0.139
U-87 MG	0.041	0.201	-0.164	0.209	0.274
DLD-1	0.018	0.090	0.059	0.076	0.084
CCRF-CEM	0.015	-0.127	0.130	0.059	0.037
A375	-0.030	0.031	0.006	-0.067	-0.277
Hs 578T	-0.033	-0.119	-0.085	0.026	0.012
769-P	-0.049	-0.032	0.698	0.078	-0.039
HCT-116	-0.053	-0.093	-0.027	0.072	0.034
A-427	-0.064	-0.073	-0.187	-0.141	-0.054
C-33 A	-0.068	-0.090	-0.238	0.181	0.184
NCI-H82	-0.074	0.216	-0.168	-0.170	-0.319
SK-N-AS	-0.080	-0.098	0.088	-0.279	-0.359
RPMI-7951	-0.098	0.069	-0.061	0.037	0.043
SJCRH30	-0.101	-0.122	-0.153	0.019	0.174
LS 174T	-0.121	-0.073	0.047	0.047	0.213
J82	-0.125	-0.100	-0.111	0.067	0.037
PA-1	-0.154	0.140	0.111	0.010	-0.105
BT-20	-0.199	-0.049	0.256	0.131	0.225
MeWo	-0.216	-0.069	0.122	0.069	0.158
BxPC-3	-0.268	-0.157	-0.168	-0.058	-0.138
MG-63	-0.268	-0.262	-0.027	-0.249	-0.255
HCT-15	-0.282	-0.256	-0.039	-0.078	-0.075
A-549	-0.308	-0.324	-0.210	0.016	0.043
U-2 OS	-0.321	-0.355	-0.358	-0.003	0.130
A-498	-0.341	-0.331	0.024	-0.044	-0.135
NCI-H460	-0.417	-0.246	-0.275	-0.093	0.010
MOLT-4	-0.424	-0.338	-0.479	-0.070	0.002
SR	-0.465	-0.202	0.033	-0.478	-0.428
SW48	-0.500	-0.362	-0.543	-0.075	-0.070
ACHN	-0.508	-0.443	-0.473	-0.149	0.038
AU-565	-0.508	-0.494	-0.478	-0.276	-0.472
AN3 CA	-0.526	-0.695	-0.523	-0.386	-0.202
OVCAR-3	-0.533	-0.577	-0.364	-0.184	-0.095
LoVo	-0.583	-0.360	-0.034	-0.142	-0.259
A-204	-1.024	-0.926	-1.018	-0.353	0.010

IC_{50} values for each cell line and the mean IC_{50} of the cell line panel were log-trans-formed. From the log IC_{50} of each cell the log IC_{50} of the panel was subtracted to determine drug sensitivity distribution within the cell panel for the respective drug (in Figure 1C). Cell lines are sorted from low to high sensitivity to PQR309.

Supplementary Table 3. Inhibitor-dependent Hill slope analysis

	Hill Slope Steepness				
**Inhibitor →**<br><br>**Cell Line**	**PQR309**	**GDC0980**	**GDC0941**	**BKM120**	**MTD147**
SHP-77	-0.708	-1.392	-11.560*	-2.432	-5.672
K-562	-0.547	-1.221	-1.101	-3.076	-3.770
SW480	-0.999	-1.381	-0.940	-2.051	-2.205
786-0	-1.180	-1.548	-1.554	-2.523	-3.720
SW620	-0.812	-0.949	-1.479	-2.131	-12.220*
CAL 27	-1.626	-1.688	-1.451	-3.157	-1.902
Jurkat E6.1	-0.566	-1.076	-1.704	-2.240	-17.800*
BT-549	-1.309	-1.479	-1.312	-2.870	-1.439
FaDu	-1.125	-1.515	-1.010	-1.624	-1.179
A-172	-0.969	-1.106	-0.920	-2.029	-2.518
U-87 MG	-0.996	-1.028	-0.826	-1.600	-2.482
DLD-1	-1.049	-1.221	-0.953	-3.194	-4.504
CCRF-CEM	-0.875	-0.692	-1.310	-3.298	-5.932
A375	-1.191	-1.322	-1.773	-4.317	-2.736
Hs 578T	-1.195	-0.935	-1.176	-3.111	-3.539
769-P	-1.421	-1.210	-1.070	-2.370	-13.430*
HCT-116	-1.467	-1.665	-1.850	-2.248	-2.865
A-427	-0.528	-0.797	-0.826	-1.880	-14.090*
C-33 A	-0.941	-1.220	-0.767	-1.598	-2.960
NCI-H82	-0.793	-0.866	-0.711	-1.787	-1.759
SK-N-AS	-1.189	-1.406	-1.352	-1.825	-2.387
RPMI-7951	-0.874	-1.350	-1.120	-2.881	-4.341
SJCRH30	-0.995	-0.712	-1.030	-1.572	-1.197
LS 174T	-1.516	-1.674	-1.534	-2.510	-3.035
J82	-0.887	-0.804	-1.686	-2.361	-2.811
PA-1	-0.759	-1.839	-1.862	-2.417	-121.100*
BT-20	-1.371	-1.209	-1.928	-2.399	-3.709
MeWo	-1.180	-1.391	-1.271	-2.576	-6.281
BxPC-3	-0.926	-1.011	-1.288	-1.579	-2.223
MG-63	-1.092	-1.391	-1.289	-1.908	-3.275
HCT-15	-1.445	-1.125	-1.056	-1.663	-3.454
A-549	-1.182	-0.972	-0.954	-2.077	-2.238
U-2 OS	-0.965	-1.317	-1.116	-1.785	-2.506
A-498	-1.254	-1.215	-1.623	-2.107	-2.430
NCI-H460	-1.129	-1.107	-0.978	-1.707	-2.105
MOLT-4	-1.184	-0.968	-1.251	-1.846	-5.071
SR	-0.872	-1.247	-1.141	-1.890	-2.729
SW48	-0.745	-1.124	-1.060	-2.039	-11.720*
ACHN	-1.221	-1.331	-0.854	-1.798	-2.024
AU-565	-1.572	-1.381	-1.186	-2.194	-14.020*
AN3 CA	-1.221	-0.916	-1.054	-2.217	-2.433
OVCAR-3	-1.153	-1.254	-1.080	-1.525	-2.579
LoVo	-1.067	-1.377	-1.335	-2.244	-3.034
A-204	-1.007	-0.980	-0.587	-1.058	-2.149

Hill slope values for each cell line. Cell lines are sorted from low to high sensitivity to PQR309. Cell lines showing for at least one drug (*) high uncertainty of slope steepness were excluded from statistical tests.

Supplementary Table 4. Inhibition of tubulin polymerization

**Name**	**pHistone H3** EC_{50} [nM] in A2058	**Condensed nuclei,** EC_{50} [nM] in A2058	**Tubulin polymerization,** IC_{50} [nM] *in vitro***
GDC0941	> 10000	> 10000*	> 45000
GDC0980	> 10000	> 5000*	> 45000
PQR309	> 10000	> 10000*	> 45000
BKM120	1471 ± 185	1434 ± 187	23070 ± 3009
BKM120-R1	> 10000	> 10000	> 45000
MTD147	741 ± 249	545 ± 107	3706 ± 809
MTD265	36.9 ± 4.2	32.4 ± 0.10	2158 ± 234
MTD265-R1	1097 ± 232	809 ± 71.0	14630 ± 2615
Colchicine	18.9 ± 5.1	18.2 ± 6.4	Not determined
Nocodazole	97.2 ± 27.7	58.1 ± 16.3	2676 ± 59.0

Cellular EC_{50} (pHistone H3 and condensed nuclei) for indicated compounds were determined as described in materials and methods section “High content/high throughput microscopy” (n=3 for GDC0941, GDC0980, PQR309, MTD147, MTD265, MTD265-R1; n= 4 (from 2 individual experiments) for BKM120-R1; n=6 for colchicine and nocodazole and n=13 for BKM120; mean ± SEM). Determination of IC_{50}s for tubulin polymerization is described in material and methods “In vitro tubulin polymerization assay”, number of experiments are given in figure legends of Supplementary Figures 2 and 4. *Incomplete curve up to > 5 µM - compound toxicity independent of mitotic arrest (compare with pHistone H3 EC_{50}); **The dynamic range of the turbidity-based microtubule polymerization assay is limited by the tubulin vs. compound stoichiometry, which sets the lowest IC_{50} detection limit to around 2-3 µM.

Supplementary Table 5. Inhibitor dissociation constants (K_d) for class I PI3Ks

	K_d, [nM] and *fold change inhibitor vs. regioisomer*			
**PI3K isoform→ Compound**	p110γ	p110α	p110β	p110δ
PIKiN1	799 ± 114	40 ± 6.5	73 ± 7.4	109 ± 4.4
PIKiN1-R1	7128 ± 1351	1239 ± 299	1192 ± 307	1158 ± 8.6
Fold change	*9 ± 2.1*	*31 ± 9.0*	*16 ± 4.5*	*10.6 ± 0.44*
PIKiN2	1264 ± 157	58 ± 11	123 ± 6.2	169 ± 3.2
PIKiN2-R1	8045 ± 756	1862 ± 321	1146 ± 143	1257 ± 11
Fold change	*6.4 ± 0.99*	*32 ± 8.2*	*9 ± 1.3*	*7.4 ± 0.16*
MTD265-R1	777 ± 74	76 ± 13	96 ± 3.5	125 ± 13
MTD265	5819 ± 263	1783 ± 319	913 ± 152	1232 ± 6.7
Fold change	*7.5 ± 0.79*	*23 ± 5.8*	*10 ± 1.6*	*10 ± 1.0*
PQR309	207 ± 33	18 ± 2.4	12 ± 3.9	19 ± 0.3
BKM120	494 ± 18	22 ± 4.5	48 ± 12	52 ± 1.4
MTD147	1549 ± 66	236 ± 28	331 ± 0.1	224 ± 9.5
BKM120-R1	621 ± 61	71 ± 11	25 ± 5.4	59 ± 1.5
PIKiN3	873 ± 23	93 ± 33	72 ± 11	105 ± 3.1

Dissociation constants (K_d) of indicated inhibitors were determined for recombinant p110γ, and p85α–p110α, –p110β and –p110δ complexes using LanthaScreen technology and the formula

$$K_{d\ inhibitor} = IC_{50} / (1 + ([Tracer]/K_{d\ Tracer}))$$

Tracer concentrations and $K_{d\ Tracer}$ values for each PI3K are listed in materials and methods. For each experiment n ≥4, performed in at least two independent experiments (mean ± SEM).

Supplementary Table 6. Inhibition of PI3K - overview

Compound	p110α-binding K_i [nM]	p110α-binding IC_{50} [nM]	p110α activity IC_{50} [nM]	pPKB IC_{50} [nM] in A2058
GDC0941	3.1 ± 0.32	30.7 ± 3.2	27.1 ± 3.4	19.0 ± 3.2
GDC0980	6.8 ± 0.38	67.4 ± 3.7	20.8 ± 0.29	41.5 ± 7.3
PQR309	17.6 ± 2.4	173.9 ± 23.5	59.3 ± 1.0	76.1 ± 6.6
BKM120	22.3 ± 4.5	219.4 ± 43.8	127.9 ± 27.2	258.2 ± 39.2
BKM120-R1	71.2 ± 10.6	701.6 ± 104.8	191.0 ± 4.8	983.3 ± 124.6
MTD147	236.3 ± 28.3	2327.3 ± 278.6	436.9 ± 101.4	788.1 ± 108.5
MTD265	1782.8 ± 319.3	17560.0 ± 3157.2	2586.0 ± 308.0	[1056.0 ± 256.6]*
MTD265-R1	75.5 ± 12.9	746.0 ± 126.7	215.1 ± 56.5	860.8 ± 79.8
PIKiN1	39.5 ± 6.5	389.3 ± 63.6	103.3 ± 5.5	678.8 ± 75.3
PIKiN1-R1	1238.5 ± 299.1	12198.0 ± 2945.9	1617.0 ± 282.2	10250.0 ± 2014.0
PIKiN2	58.3 ± 10.9	574.1 ± 107.3	265.9 ± 3.2	1874.0 ± 307.8
PIKiN2-R1	1861.5 ± 320.5	18335.0 ± 3157.2	4317.0 ± 786.5	> 10000
PIKiN3	92.7 ± 32.7	913.4 ± 321.8	360.8 ± 131.5	2545.0 ± 455.8
Colchicine	> 10000	> 10000	>10000	[1539.0 ± 459.1]*
Nocodazole	> 10000	> 10000	>10000	> 10000

Determination of p110α-binding parameters (K_i and IC_{50}) is described in material and methods "*In vitro* PI3K inhibitor binding assay"; $n \geq 3$, mean ± SEM. Some K_i of p110α values already shown in Supplementary Table 5 are included here for clarity. Determination of the IC_{50} of PI3Kα activity is described under "PtdIns(3,4,5)P_3 production/ PI3Kα activity". Dose response curves are duplicates, for MTD265, PIKiN1-R1 and PIKiN2-R1 in quadruplicate; mean ± SEM. Inhibition of cellular phosphorylation of PKB/Akt in A2058 cells is described under "In-cell western detection of phosphorylated PKB/Akt", $n \geq 4$, mean ± SEM. *Low value due to effect in cell viability, evaluation impaired due to biphasic dose response curve).

Supplementary Table 7. Data collection and refinement statistics – tubulin

	**Tubulin-BKM120 (PDB ID 5M7E)**	**Tubulin-MTD147 (PDB ID 5M7G)**	**Tubulin-MTD265-R1 (PDB ID 5M8D)**	**Tubulin-MTD265 (PDB ID 5M8G)**
**Data collection**[a]				
Space group	$P2_12_12_1$	$P2_12_12_1$	$P2_12_12_1$	$P2_12_12_1$
Cell dimensions				
a, b, c (Å)	104.6, 158.0, 179.7	104.1, 156.9, 179.7	104.2, 157.0, 180.0	104.8, 157.5, 179.2
Resolution (Å)	44.9 - 2.05 (2.10 – 2.05)	46.7 – 2.25 (2.38 – 2.25)	68.1 - 2.25 (2.31 – 2.25)	49.3 – 2.15 (2.21 – 2.15)
R_{meas} (%)	11.8 (196.6)	11.4 (196.0)	12.1 (192.7)	13.3 (268.0)
$CC_{1/2}$[b]	99.9 (35.9)	99.9 (33.5)	99.9 (53.9)	99.9 (41.4)
I/σI	12.5 (1.0)	15.8 (1.0)	17.0 (1.4)	17.6 (1.1)
Completeness (%)	99.8 (98.7)	99.1 (96.5)	99.5 (96.0)	100.0 (99.9)
Redundancy	7.0 (7.0)	6.7 (6.5)	13.4 (11.6)	13.5 (12.8)
**Refinement**				
Resolution (Å)	44.9 – 2.05	46.7 – 2.25	68.1 - 2.25	49.3 – 2.15
No. unique reflections	187079	138844	139438	161400
R_{work}/R_{free} (%)	17.3 /20.3	17.2 / 21.7	17.0 / 21.0	17.1 / 20.8
Average B-factors ($Å^2$)				
protein	57.6	66.2	63.3	64.2
solvent	54.3	60.3	56.3	56.2
ligands (chain B/D)	55.2 / -	50.1 / 52.0	59.1 / -	44.1 / 51.8
Wilson B-factor	41.3	48.8	45.1	45.5
Root mean square deviation from ideality				
Bond length (Å)	0.007	0.008	0.007	0.007
Bond angles (°)	0.880	0.910	0.896	0.893
Ramachandran statistics[c]				
Favored regions (%)	98.7	98.0	97.9	98.1
Allowed regions (%)	1.3	1.9	2.0	1.8
Outliers (%)	0.0	0.1	0.1	0.1

[a]Highest shell statistics are in parentheses. [b]$CC_{1/2}$= percentage of correlation between intensities from random half-datasets. [c]As defined by MolProbity.

Supplementary Table 8. Data collection and refinement statistics – PI3Kγ

	**PI3Kγ-PIKiN2 (PDB ID 5JHA)**	**PI3Kγ-PIKiN3 (PDB ID 5JHB)**
**Data collection**[a]		
Space group	C 1 2 1	C 1 2 1
Cell dimensions		
a, b, c (Å)	144.56, 68.04, 106.73	142.53, 67.6, 106.55
α, β, γ (°)	90, 95.4, 90	90, 96.2, 90
Resolution (Å)	61.51 - 2.51 (2.60 - 2.51)	62.08 - 2.48 (2.57 - 2.48)
R_{meas} (%)	5.9 (65.1)	3.5 (52.9)
$CC_{1/2}$[b]	99.8 (72.8)	99.8 (78.8)
I/σI	17.7 (2.20)	17.6 (2.45)
Completeness (%)	97.4 (98.2)	98.0 (99.6)
Redundancy	3.4 (3.5)	3.2 (3.3)
**Refinement**		
Resolution (Å)	61.51 - 2.51	62.08 - 2.48
No. unique reflections	34660 (3462)	35273 (3540)
R_{work}/R_{free} (%)	20.2 / 24.5	23.1 / 27.7
Average B-factors ($Å^2$)		
protein	100.9	102.1
solvent	74.3	67.8
ligands	105.4	87.0
Wilson B-factor	73.5	70.3
Root mean square deviation from ideality		
Bond length (Å)	0.003	0.003
Bond angles (°)	0.53	0.58
Ramachandran statistics[c]		
Favored regions (%)	97	97
Allowed regions (%)	3	2.8
Outliers (%)	0.0	0.13

[a]Highest shell statistics are in parentheses. [b]$CC_{1/2}$= percentage of correlation between intensities from random half-datasets. [c]As defined by MolProbity.

SUPPLEMENTARY METHODS

Synthetic Chemistry

General Information and Analytical Methods

Reagents were purchased at the highest commercial quality from Acros or Sigma-Aldrich and used without further purification, unless otherwise stated. Solvents were purchased from Acros Organics in AcroSeal® bottles over molecular sieves. Column chromatographic purifications were performed on Merck KGaA silica gel (pore size 60 Å, 230-400 mesh particle size).

Cross coupling reactions were carried out under a positive pressure of nitrogen in anhydrous solvents, and the reaction flasks were typically fitted with rubber septa for the introduction of substrates and reagents via syringe. Glassware was oven dried prior to use.

Thin Layer Chromatography (TLC)

TLC plates were obtained from Merck KGaA (Polygram SIL/UV254, 0.2 mm silica with fluorescence indicator). UV light (254 nm) or basic permanganate solution were used to visualize the respective compounds.

Nuclear magnetic resonance spectroscopy (NMR)

^{1}H NMR spectra were recorded on a Bruker Avance 400 spectrometer operating at 400 MHz. ^{1}H and ^{13}C NMR spectra were obtained for solutions in various deuterated solvents such as $CDCl_3$ or $(CD_3)_2SO$. The chemical shift (δ values) are reported in ppm and corrected to the signal of the deuterated solvents (7.26 ppm (^{1}H NMR) and 77.16 ppm (^{13}C NMR) for $CDCl_3$; and 2.50 ppm (^{1}H NMR) and 39.52 ppm (^{13}C NMR) for $(CD_3)_2SO$). ^{19}F NMR spectra were calibrated relative to $CFCl_3$ (δ = 0 ppm) as external standard. ^{19}F NMR spectra were recorded ^{1}H-decoupled. When peak multiplicities are reported, the following abbreviations are used: s (singlet), d (doublet), t (triplet), m (multiplet), br (broadened). Coupling constants, when given, are reported in Hertz (Hz).

High Resolution Mass Spectrometry (HRMS)

High resolution mass spectra were recorded on a Thermo Fisher Scientific LTQ Orbitrap XL (ESI-MS) spectrometer.

High Performance Liquid Chromatography (HPLC)

HPLC analyses were performed on an Ultimate 3000SD System from ThermoFisher with LPG-3400SD pump system, ACC-3000 autosampler and column oven, and DAD-3000 diode array detector. An Acclaim-120 C18 reversed-phase column from ThermoFisher was used as stationary phase.

General Procedures:

General Procedure 1: Under nitrogen atmosphere, the respective chloropyrimidine compound (1.0 eq.), boronic ester **3** (1.2 eq.), potassium phosphate tribasic (2.0 eq.) and choloro(2-dicyclohexylphosphino-2',4',6'-triisopropyl-1,1'-biphenyl)(2-(2'-amino-1,1'-biphenyl))-palladium(II) (0.05 eq.) were dissolved in a mixture of 1,4-dioxane (~ 30 volumes) and deionized H_2O (~ 10 volumes). The reaction mixture was then placed into a pre-heated oil bath at 95 °C for 16 hours. Upon completion of the reaction, the mixture was allowed to cool down to room temperature, quenched with an aqueous 2 M NaOH-solution and extracted with dichloromethane (3 x). The combined organic layer was dried over anhydrous Na_2SO_4, filtered and concentrated to dryness under reduced pressure. The crude product was purified by column chromatography on silica gel.

General Procedure 2: To a solution of *N*,*N*-dimethyl-*N'*-(5-(4,4,5,5-tetramethyl-1,3,2-dioxaborolan-2-yl)-4-(trifluoromethyl)pyridin-2-yl)formimidamide (**3**, 1.1 eq.) in tetrahydrofuran (~ 0.3 volumes) were added successively, Pd(dppf)Cl_2 (0.05 eq.), the respective chloropyrimidine / -triazine compound (1.0 eq.) and a 2.4 M aqueous K_2CO_3-solution (3.0 eq.). The resulting mixture was heated at 80 °C for 16 hours. After cooling down to room temperature, the reaction was diluted with an aqueous saturated NH_4Cl-solution and extracted with ethyl acetate (3 x). The combined organic layer was washed with deionized H_2O, dried over anhydrous Na_2SO_4, filtered and concentrated to dryness under reduced pressure. The above crude residue was then dissolved in methanol (~ 20 volumes) and a 4 M solution of HCl in dioxane (> 5.0 eq.) was slowly added. The resulting solution was then heated at 90 °C for 6 hours. Upon completion of the reaction, the mixture was allowed to cool down to room temperature, quenched with a 2 M aqueous NaOH-solution and extracted with ethyl acetate (3 x). The combined organic layer was dried over anhydrous Na_2SO_4, filtered and then concentrated to dryness under reduced pressure. The residue was purified by column chromatography on silica gel.

General Procedure 3: The respective dichloropyridine compound (1.0 eq.), the desired secondary amine (1.1 - 1.5 eq.) and *N*,*N*-diisopropylethylamine (1.3 eq.) were mixed in ethanol (~ 3 volumes) and heated at reflux for 16 hours. After cooling down to room temperature, the mixture was diluted with ethyl acetate and washed with an aqueous saturated $NaHSO_4$-solution (2 x). The organic layer was dried over anhydrous Na_2SO_4, filtered and then concentrated to dryness under reduced pressure. The residue was purified by column chromatography on silica gel.

Synthesis of Intermediates and Reagents

*N'-(5-Bromo-4-(trifluoromethyl)pyridin-2-yl)-N,N-dimethylformimidamide (**2**):*

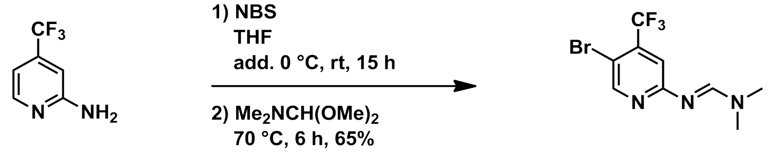

1 2

At 0 °C in an ice-bath, *N*-bromosuccinimide (29.2 g, 164 mmol, 1.1 eq.) was slowly added to a solution of 2-amino-6-(trifluoromethyl)pyridine (**1**, 25.0 g, 156 mmol, 1.0 eq.) in tetrahydrofuran (250 ml). The resulting mixture was then stirred at room temperature for 15 hours. Then, *N*,*N*-dimethylformamide dimethyl acetal (25.1 ml, 188 mmol, 1.2 eq.) was added and the mixture heated at 70 °C for 6 hours. The solvent was evaporated under reduced pressure and the residue was recrystallized from hexane to yield product **2** as an off-white solid (30.0 g, 101 mmol, 65% yield).

Compound **2**: ^{1}**H NMR** (400 MHz, $CDCl_3$): δ 8.46 (s, 1 H), 8.38 (s, 1 H), 7.18 (s, 1 H), 3.11 (s, 3 H), 3.08 (s, 3 H); ^{19}**F{**^{1}**H} NMR** (376 MHz, $CDCl_3$): δ − 65.0 (s, 3 F); ^{13}**C{**^{1}**H} NMR** (101 MHz, $CDCl_3$): δ 162.0 (s, 1 C), 156.0 (s, 1 C), 152.1 (s, 1 C), 138.5 (q, $^{2}J_{C,F}$ = 32 Hz, 1 C), 122.0 (q, $^{1}J_{C,F}$ = 274 Hz, 1 C), 116.7 (q, $^{3}J_{C,F}$ = 5.2 Hz, 1 C), 108.1-107.9 (m, 1 C), 41.1 (s, 1 C), 34.9 (s, 1 C); **HRMS** (*m/z*): calc. for $C_9H_{10}BrF_3N_3$ ($[M + H]^+$): 296.0005; found, 295.9998.

*N,N-Dimethyl-N'-(5-(4,4,5,5-tetramethyl-1,3,2-dioxaborolan-2-yl)-4-(trifluoromethyl)pyridin-2-yl)formimidamide (**3**):*

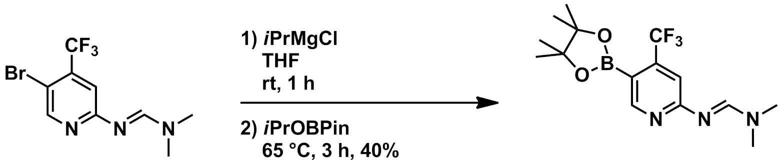

2 3

At 0 °C in an ice-bath, a 2 M solution of isopropylmagnesium chloride in tetrahydrofuran (38.9 ml, 77.8 mmol, 1.2 eq.) was slowly added to a solution of *N'*-(5-bromo-4-(trifluoromethyl)pyridin-2-yl)-*N*,*N*-dimethylformimidamide (**2**, 20.0 g, 67.6 mmol, 1.0 eq.) in tetrahydrofuran (140 ml). The resulting yellowish suspension was stirred at room temperature for 1 hour. Then, 2-isopropoxy-4,4,5,5-tetramethyl-1,3,2-dioxaborolane (19.1 ml, 94.6 mmol, 1.4 eq.) was added and the reaction mixture was placed in a preheated oil bath at 65 °C. After 3 hours, the reaction mixture was cooled down to room temperature and poured onto an aqueous saturated NH_4Cl-solution. The organic layer was separated and reduced to dryness under reduced pressure. The residue was then dissolved in hexanes and washed with an aqueous saturated $NaHCO_3$-solution (2 x). The organic layer was dried over anhydrous Na_2SO_4, filtered and the solvent was evaporated under reduced pressure.

The residue was recrystallized from hexanes to afford the desired compound **3** as a yellowish solid (9.28 g, 27.0 mmol, 40% yield).

Compound **3**: **^{1}H NMR** (400 MHz, $CDCl_3$): δ 8.58 (s, 1 H), 8.51 (s, 1 H), 7.14 (s, 1 H), 3.09 (s, 3 H), 3.08 (s, 3 H), 1.32 (s, 12 H); **^{19}F{^{1}H} NMR** (376 MHz, $CDCl_3$): δ − 62.7 (s, 3 F); **^{13}C{^{1}H} NMR** (101 MHz, $CDCl_3$): δ 164.4 (s, 1 C), 156.2 (s, 1 C), 155.8 (s, 1 C), 143.5 (q, $^{2}J_{C,F}$ = 33 Hz, 1 C), 123.2 (q, $^{1}J_{C,F}$ = 274 Hz, 1 C), 115.4-113.0 (br s, 1 C), 114.4 (q, $^{3}J_{C,F}$ = 5.1 Hz, 1 C), 84.3 (s, 2 C), 41.0 (s, 1 C), 34.9 (s, 1 C), 24.7 (s, 4 C); **HRMS** (*m/z*): calc. for $C_{15}H_{22}O_2N_3BF_3$ ($[M + H]^+$): 344.1752; found, 344.1742.

*tert-Butyl (5-bromo-4-(trifluoromethyl)pyridin-2-yl)carbamate (**4**):*

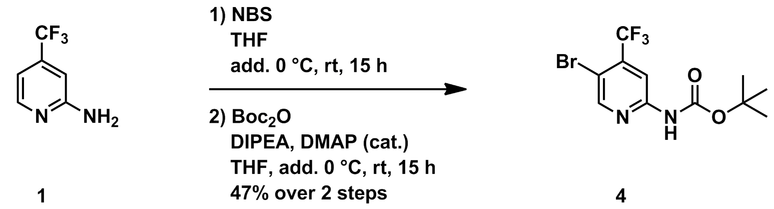

At 0 °C in an ice-bath, *N*-bromosuccinimide (29.2 g, 164 mmol, 1.1 eq.) was slowly added to a solution of 2-amino-6-(trifluoromethyl)pyridine (**1**, 25.0 g, 156 mmol, 1.0 eq.) in tetrahydrofuran (250 ml) and the resulting reaction mixture was then stirred at room temperature for 15 hours. Upon completion of the reaction, the mixture was diluted with ethyl acetate and the organic layer was separated, washed with an aqueous saturated Na_2CO_3-solution, dried over anhydrous Na_2SO_4, filtered and reduced to dryness under reduced pressure. The above crude residue was dissolved in tetrahydrofuran (250 ml) and 4-(dimethylamino)pyridine (1.90 g, 15.6 mmol, 0.10 eq.) was added, followed by *N*,*N*-diisopropylethylamine (27.2 ml, 156 mmol, 1.0 eq.). The resulting mixture was cooled down to 0 °C in an ice-bath and a solution of di-*tert*-butyl dicarbonate (34.0 g, 156 mmol, 1.0 eq.) in tetrahydrofuran (350 ml) was added dropwise. After the addition was complete, the ice-bath was removed and the reaction mixture was allowed to stir at room temperature for 15 hours. After this time, the solvent was evaporated under reduced pressure and the residue was purified by column chromatography on silica gel (cyclohexane / ethyl acetate 9:2 → 3:1) to yield product **4** as a colorless semisolid (25.0 g, 73.3 mmol, 47% yield).

Compound **4**: **^{1}H NMR** (400 MHz, $CDCl_3$): δ 9.47 (br s, 1 H), 8.60 (s, 1 H), 8.43 (s, 1 H), 1.59 (s, 9 H); **^{19}F{^{1}H} NMR** (376 MHz, $CDCl_3$): δ − 64.9 (s, 3 F); **^{13}C{^{1}H} NMR** (101 MHz, $CDCl_3$): δ 152.6 (s, 1 C), 152.3 (s, 1 C), 151.8 (s, 1 C), 139.6 (q, $^{2}J_{C,F}$ = 33 Hz, 1 C), 121.8 (q, $^{1}J_{C,F}$ = 275 Hz, 1 C), 111.1 (q, $^{3}J_{C,F}$ = 5.5 Hz, 1 C), 109.0 (br s, 1 C), 82.4 (s, 1 C), 28.5 (s, 3 C); **HRMS** (*m/z*): calc. for $C_{11}H_{13}BrF_3N_2O_2$ ($[M + H]^+$): 341.0107; found, 341.0107. Recently an optimized procedure for the preparation of compound **4** has been reported[1].

Synthesis of Intermediates and Final Compounds with Pyridine Core

*tert-Butyl (2',6'-dichloro-4-(trifluoromethyl)-(3,4'-bipyridin)-6-yl)carbamate (**6**):*

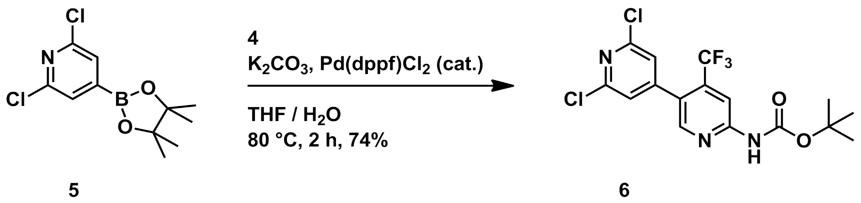

To a solution of 2,6-dichloropyridyl-4-boronic acid pinacol ester (**5**, 1.00 g, 3.65 mmol, 1.0 eq.) in tetrahydrofuran (20 ml) were added successively, Pd(dppf)Cl_2 (134 mg, 0.183 mmol, 0.05 eq.), *tert*-butyl (5-bromo-4-(trifluoromethyl)pyridin-2-yl)carbamate (**4**, 1.24 g, 3.65 mmol, 1.0 eq.) and a 2.4 M aqueous K_2CO_3-solution (4.60 ml, 11.0 mmol, 3.0 eq.). The resulting solution was then heated at 80 °C for 2 hours. Upon completion of the reaction, the mixture was allowed to cool down to room temperature, quenched with aqueous saturated NH_4Cl-solution and extracted with ethyl acetate (3 x). The combined organic layer was washed with deionized H_2O, dried over anhydrous Na_2SO_4, filtered and concentrated under reduced pressure. The residue was purified by column chromatography on silica gel (cyclohexane / ethyl acetate 9:1 → 3:1). The desired product **6** was obtained as an off-white solid (1.10 g, 2.69 mmol, 74% yield).

Compound **6**: **^{1}H NMR** (400 MHz, $CDCl_3$): δ 9.46 (s, 1 H), 8.50 (s, 1 H), 8.32 (s, 1 H), 7.25 (s, 2 H), 1.53 (s, 9 H); **^{19}F{^{1}H} NMR** (376 MHz, $CDCl_3$): δ − 59.3 (s, 3 F); **^{13}C{^{1}H} NMR** (101 MHz, $CDCl_3$): δ 154.3 (s, 1 C), 152.2 (s, 1 C), 150.8 (s, 2 C), 149.4 (s, 1 C), 149.3 (s, 1 C), 138.2 (q, $^2J_{C,F}$ = 32 Hz, 1 C), 124.5 (br s, 1 C), 123.5 (s, 2 C), 122.4 (q, $^1J_{C,F}$ = 275 Hz, 1 C), 109.3 (q, $^3J_{C,F}$ = 5.3 Hz, 1 C), 82.6 (s, 1 C), 28.4 (s, 3 C); **HRMS** (*m/z*): calc. for $C_{16}H_{15}Cl_2F_3N_3O_2$ ($[M + H]^+$): 408.0488; found, 408.0485.

*2',6'-Dimorpholino-4-(trifluoromethyl)-(3,4'-bipyridin)-6-amine (**MTD147**):*

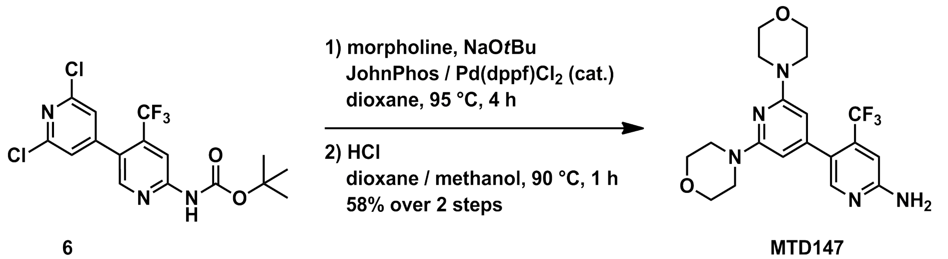

To a solution of compound **6** (1.20 g, 2.94 mmol, 1.0 eq.) in 1,4-dioxane (20 ml) were added successively, JohnPhos (175 mg, 588 µmol, 0.20 eq.), morpholine (2.57 ml, 29.4 mmol, 10 eq.) and NaO*t*Bu (2.82 g, 29.4 mmol, 10 eq.). Then, Pd(dppf)Cl_2 (215 mg, 294 µmol, 0.1 eq.) was added and the resulting mixture was placed into a pre-heated oil bath at 95 °C for 4 hours. Upon completion of the reaction, the mixture was allowed to cool down to room temperature, quenched with aqueous saturated NH_4Cl-solution and

extracted with ethyl acetate (3 x). The combined organic layer was washed with deionized H_2O, dried over anhydrous Na_2SO_4, filtered and then concentrated to dryness under reduced pressure. The above crude residue was then dissolved in methanol (5 ml) and a 4 M solution of HCl in dioxane (20 ml) was slowly added. The resulting solution was heated at 90 °C for 1 hour. After this time, the reaction mixture was allowed to cool down to room temperature, poured onto a 2 M aqueous NaOH-solution and the aqueous layer was extracted with ethyl acetate (3 x). The combined organic layer was washed with deionized H_2O, dried over anhydrous Na_2SO_4, filtered and then concentrated to dryness under reduced pressure. The residue was purified by column chromatography on silica gel (cyclohexane / ethyl acetate 1:3 → 0:1) to yield the desired product **MTD147** as a colorless solid (699 mmol, 1.71 mmol, 58% yield).

Compound **MTD147**: **^{1}H NMR** (600 MHz, $(CD_3)_2SO$): δ 7.95 (s, 1 H), 6.81 (s, 1 H), 6.60 (s, 2 H), 6.00 (br s, 2 H), 3.70-3.64 (m, 8 H), 3.41-3.36 (m, 8 H); **^{19}F{^{1}H} NMR** (376 MHz, $CDCl_3$): δ − 59.9 (s, 3 F); **^{13}C{^{1}H} NMR** (151 MHz, $(CD_3)_2SO$): δ 159.8 (s, 1 C), 157.7 (s, 2 C), 150.2 (s, 1 C), 147.8 (s, 1 C), 135.2 (q, $^2J_{C,F}$ = 30 Hz, 1 C), 123.2 (q, $^1J_{C,F}$ = 275 Hz, 1 C), 121.5 (br s, 1 C), 103.6 (q, $^3J_{C,F}$ = 5.2 Hz, 1 C), 97.2 (s, 2 C), 66.0 (s, 4 C), 45.1 (s, 4 C); **HRMS** (*m/z*): calc. for $C_{19}H_{23}F_3N_5O_2$ ($[M + H]^+$): 410.1798; found, 410.1787; **HPLC** (Acclaim-120 C18, $CH_3CN:TFA_{(99.9:0.1)}$ / $MeOH:H_2O_{(10:90)}$ 5:95 for 0.2 min, 5:95 → 100:0 over 10 min, 100:0 for 3 min, 0.5 ml / min, 40 °C, 254 nm): t_R = 6.31 min.

Synthesis of Intermediates and Final Compounds with Pyrimidine Core

*4,4'-(6-Chloropyrimidine-2,4-diyl)dimorpholine (**8**) and 4,4'-(2-chloropyrimidine-4,6-diyl)dimorpholine (**9**):*

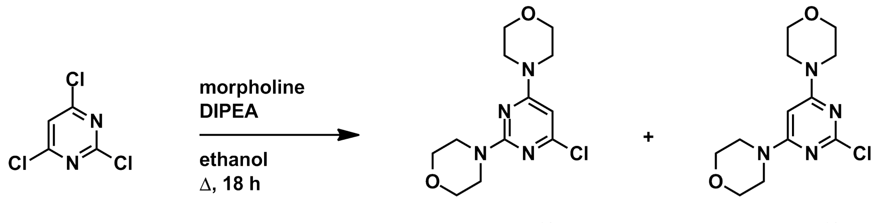

7

8, 80%

9, 13%

2,4,6-Trichloropyrimidine (**7**, 7.00 ml, 60.9 mmol, 1.0 eq.), morpholine (11.7 ml, 134 mmol, 2.2 eq.) and *N*,*N*-diisopropylethylamine (23.3 ml, 134 mmol, 2.2 eq.) were mixed in ethanol (120 ml) and heated at reflux for 18 hours. Then, the solvent was evaporated and the resulting residue was dissolved in dichloromethane, washed with an aqueous saturated $NaHSO_4$-solution, dried over anhydrous Na_2SO_4 and concentrated under reduced pressure. The products were separated by column chromatography on silica gel (cyclohexane / ethyl acetate 3:1 → 1:1). Compounds **8** (13.9 g, 48.8 mmol, 80% yield) and **9** (2.25 g, 7.90 mmol, 13% yield) were both isolated as colorless solids.

Compound **8**: **^{1}H NMR** (400 MHz, $CDCl_3$): δ 5.86 (s, 1 H), 3.76-3.69 (m, 12 H), 3.56-3.51 (m, 4 H); **^{13}C{^{1}H} NMR** (101 MHz, $CDCl_3$): δ 163.5 (s, 1 C), 160.9 (s, 1 C), 160.6 (s, 1 C), 91.2 (s, 1 C), 66.9 (s, 2 C), 66.5 (s, 2 C), 44.5 (s, 2 C), 44.4 (s, 2 C). The spectroscopic data are consistent with previous literature reports[2].

Compound **9**: **^{1}H NMR** (400 MHz, $CDCl_3$): δ 5.38 (s, 1 H), 3.76-3.72 (m, 8 H), 3.55-3.50 (m, 8 H); **^{13}C{^{1}H} NMR** (101 MHz, $CDCl_3$): δ 164.5 (s, 2 C), 160.0 (s, 1 C), 79.1 (s, 1 C), 66.5 (s, 4 C), 44.7 (s, 4 C).

*5-(2,6-Dimorpholinopyrimidin-4-yl)-4-(trifluoromethyl)pyridin-2-amine (**BKM120**):*

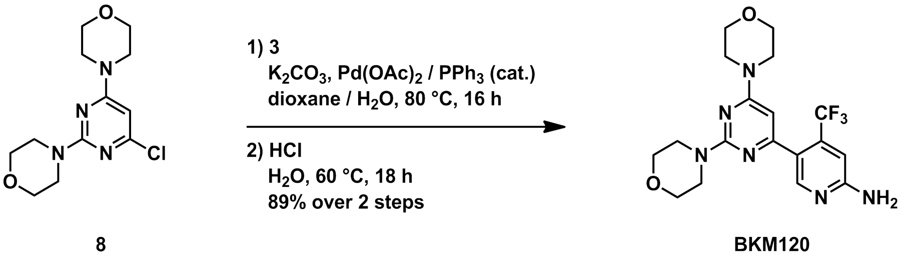

$Pd(OAc)_2$ (53.0 mg, 236 µmol, 0.04 eq.) and PPh_3 (182 mg, 694 µmol, 0.12 eq.) were dissolved in 1,4-dioxane (10 ml) and stirred at room temperature for 1 hour. This solution was then added to a mixture of compound **8** (1.70 g, 5.97 mmol, 1.0 eq.), *N*,*N*-dimethyl-*N'*-(5-(4,4,5,5-tetramethyl-1,3,2-dioxaborolan-2-yl)-4-(trifluoromethyl)pyridin-2-yl)formimidamide (**3**, 2.05 g, 5.97 mmol, 1.0 eq.) and aqueous K_2CO_3 (2.4 M, 8.00 ml, 19.2 mmol, 3.2 eq.) in 1,4-dioxane (20 ml). The resulting reaction mixture was then heated at 80 °C for 16 hours. After this time, the reaction mixture was allowed to cool down to room temperature and a 5 M aqueous HCl-solution (12.0 ml, 60.0 mmol, 10 eq.) was slowly added. Then, the mixture was again heated at 60 °C for 18 hours. Upon completion of the reaction, the mixture was allowed to cool down to room temperature, quenched with 2 M aqueous NaOH-solution and extracted with ethyl acetate (3 x). The combined organic layer was dried over anhydrous Na_2SO_4, filtered and then concentrated to dryness under reduced pressure. The residue was purified by column chromatography on silica gel (cyclohexane / ethyl acetate 1:3 → 0:1) to afford the desired product **BKM120** as a colorless solid (2.19 g, 5.34 mmol, 89% yield).

Compound **BKM120**: **^{1}H NMR** (500 MHz, $CDCl_3$): δ 8.25 (s, 1 H), 6.76 (s, 1 H), 5.96 (s, 1 H), 4.87 (br s, 2 H), 3.79-3.71 (m, 12 H), 3.61-3.57 (m, 4 H); **^{19}F{^{1}H} NMR** (376 MHz, $CDCl_3$): δ − 59.7 (s, 3 F); **^{13}C{^{1}H} NMR** (151 MHz, $CDCl_3$): δ 163.3 (s, 1 C), 163.0 (s, 1 C), 161.5 (s, 1 C), 158.9 (s, 1 C), 151.0 (s, 1 C), 137.9 (q, $^{2}J_{C,F}$ = 32 Hz, 1 C), 124.4 (s, 1 C), 123.0 (q, $^{1}J_{C,F}$ = 275 Hz, 1 C), 105.3 (q, $^{3}J_{C,F}$ = 5.3 Hz, 1 C), 92.5 (s, 1 C), 67.1 (s, 2 C), 66.7 (s, 2 C), 44.5 (s, 2 C), 44.4 (s, 2 C); **HPLC** (Acclaim-120 C18, CH_3CN:TFA $_{(99.9:0.1)}$ / MeOH:$H_2O_{(10:90)}$ 5:95 for 0.2 min, 5:95 → 100:0 over 10 min, 100:0 for 3 min, 0.5 ml / min, 40 °C, 254 nm): t_R = 5.26 min. The spectroscopic data are consistent with previous literature reports[2].

*5-(4,6-Dimorpholinopyrimidin-2-yl)-4-(trifluoromethyl)pyridin-2-amine (**BKM120-R1**):*

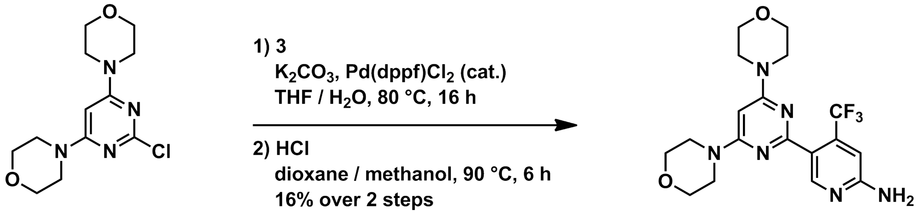

9 **BKM120-R1**

This compound was prepared according to general procedure 2 from *N*,*N*-dimethyl-*N'*-(5-(4,4,5,5-tetramethyl-1,3,2-dioxaborolan-2-yl)-4-(trifluoromethyl)pyridin-2-yl)formimidamide (**3**, 700 mg, 2.04 mmol, 1.1 eq.) and compound **9** (530 mg, 1.86 mmol, 1.0 eq.). After purification by column chromatography on silica gel (cyclohexane / ethyl acetate 1:3 → 0:1), the desired product **BKM120-R1** was obtained as a colorless solid (122 mg, 297 µmol, 16% yield).

Compound **BKM120-R1**: ^{1}**H NMR** (400 MHz, $CDCl_3$): δ 8.66 (s, 1 H), 6.78 (s, 1 H), 5.51 (s, 1 H), 4.75 (br s, 2 H), 3.81-3.75 (m, 8 H), 3.63-3.55 (m, 8 H); ^{19}**F{^{1}H} NMR** (376 MHz, $CDCl_3$): δ − 59.9 (s, 3 F); ^{13}**C{^{1}H} NMR** (101 MHz, $CDCl_3$): δ 163.9 (s, 2 C), 162.1 (s, 1 C), 158.9 (s, 1 C), 152.6 (s, 1 C), 138.0 (q, $^{2}J_{C,F}$ = 31 Hz, 1 C), 124.1 (s, 1 C), 123.1 (q, $^{1}J_{C,F}$ = 274 Hz, 1 C), 105.3-104.9 (m, 1 C), 79.4 (s, 1 C), 66.7 (s, 4 C), 44.7 (s, 4 C); **HRMS** (*m/z*): calc. for $C_{18}H_{22}F_3N_6O_2$ ($[M + H]^+$): 411.1751; found, 411.1740; **HPLC** (Acclaim-120 C18, CH_3CN:TFA $_{(99.9:0.1)}$ / $MeOH:H_2O_{(10:90)}$ 5:95 for 0.2 min, 5:95 → 100:0 over 10 min, 100:0 for 3 min, 0.5 ml / min, 40 °C, 254 nm): t_R = 5.57 min.

*4-(4,6-Dichloropyrimidin-2-yl)morpholine (**10**) and 4-(2,6-dichloropyrimidin-4-yl)morpholine (**11**):*

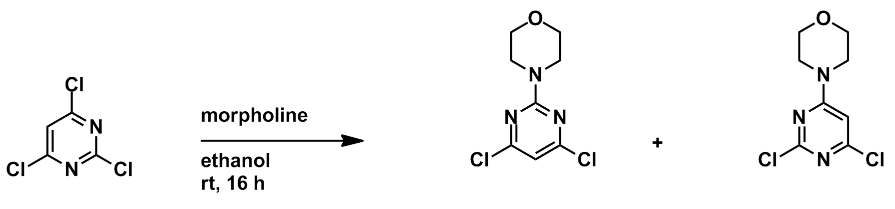

7 **10**, 20% **11**, 65%

2,4,6-Trichloropyrimidine (**7**, 14.0 ml, 122 mmol, 1.0 eq.) and morpholine (22.4 ml, 512 mmol, 4.2 eq.) were mixed in ethanol (250 ml) and then stirred at room temperature for 16 hours. Then, the reaction mixture was poured onto an aqueous saturated $NaHSO_4$-solution. The resulting precipitate was isolated by filtration and the products were separated by column chromatography on silica gel (cyclohexane / ethyl acetate 3:1). Compounds **10** (5.71 g, 24.4 mmol, 20% yield) and **11** (18.6 g, 79.5 mmol, 65% yield) were isolated as colorless solids.

Compound **10**: **^{1}H NMR** (400 MHz, $CDCl_3$): δ 6.53 (s, 1 H), 3.82-3.74 (m, 4 H); 3.74-3.66 (m, 4 H); **^{13}C{^{1}H} NMR** (101 MHz, $CDCl_3$): δ 161.7 (s, 2 C), 160.6 (s, 1 C), 108.3 (s, 1 C), 66.6 (s, 2 C), 44.4 (s, 2 C); **HRMS** (*m/z*): calc. for $C_8H_{10}Cl_2N_3O$ ([M + H]$^+$): 234.0195; found, 234.0194.

Compound **11**: **^{1}H NMR** (400 MHz, $CDCl_3$): δ 6.34 (s, 1 H), 3.73-3.66 (m, 4 H), 3.58 (br s, 4 H); **^{13}C{^{1}H} NMR** (101 MHz, $CDCl_3$): δ 163.1 (s, 1 C), 160.4 (s, 1 C), 159.7 (s, 1 C), 99.8 (s, 1 C), 66.1 (s, 2 C), 44.5 (br s, 2 C); **HRMS** (*m/z*): calc. for $C_8H_{10}Cl_2N_3O$ ([M + H]$^+$): 234.0195; found, 234.0192.

*6-(6-Chloro-2-morpholinopyrimidin-4-yl)-2-oxa-6-azaspiro[3.3]heptane (**12**):*

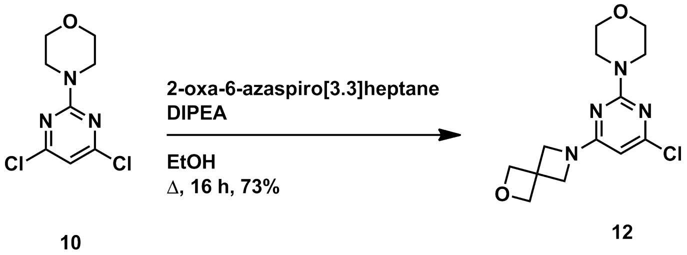

This compound was prepared according to general procedure 3 from 4-(4,6-dichloropyrimidin-2-yl)morpholine (**10**, 1.40 g, 5.98 mmol, 1.0 eq.) and 2-oxa-6-azaspiro[3.3]heptane hemioxalate (2.58 g, 8.95 mmol, 1.5 eq.). After purification by column chromatography on silica gel (cyclohexane / ethyl acetate 2:1), the desired product **12** was obtained as a colorless solid (1.30 g, 4.38 mmol, 73% yield).

Compound **12**: **^{1}H NMR** (400 MHz, $CDCl_3$): δ 5.56 (s, 1 H), 4.82 (br s, 4 H), 4.15 (br s, 4 H), 3.76-3.67 (m, 8 H); **^{13}C{^{1}H} NMR** (101 MHz, $CDCl_3$): δ 164.1 (s, 1 C), 161.0 (s, 1 C), 159.7 (s, 1 C), 90.5 (s, 1 C), 81.0 (s, 2 C), 66.9 (s, 2 C), 59.4 (s, 2 C), 44.3 (s, 2 C), 39.2 (s, 1 C); **HRMS** (*m/z*): calc. for $C_{13}H_{18}ClN_4O_2$ ([M + H]$^+$): 297.1113; found, 297.1119.

*(1-(6-(6-Amino-4-(trifluoromethyl)pyridin-3-yl)-2-morpholinopyrimidin-4-yl)-3-(chloromethyl)azetidin-3-yl}methanol (**PIKiN2-R1**):*

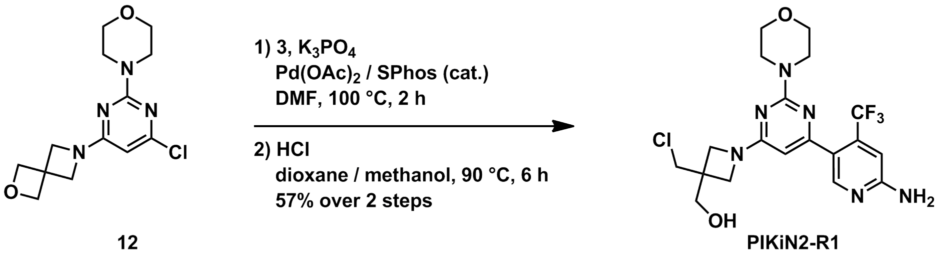

$Pd(OAc)_2$ (4.00 mg, 17.8 µmol, 0.05 eq.) and SPhos (14 mg, 34.1 µmol, 0.1 eq.) were dissolved in DMF (1 ml) and stirred at room temperature for 1 hour. This complex solution was then added to a mixture of *N*,*N*-dimethyl-*N'*-(5-(4,4,5,5-tetramethyl-1,3,2-dioxaborolan-2-yl)-4-(trifluoromethyl)pyridin-2-yl)formimidamide (**3**, 127 mg, 370 µmol, 1.1 eq.), compound **12** (100 mg, 337 µmol, 1.0 eq.) and K_3PO_4 (215 mg, 1.01 mmol, 3.0 eq.) in DMF (1 ml). Then, the resulting reaction mixture was heated at 100 °C for 2 hours. Upon completion of the reaction, the mixture was allowed to cool down to room temperature, quenched with an aqueous saturated NH_4Cl-solution and extracted with ethyl acetate (3 x). The combined organic layer was washed with deionized H_2O, dried over anhydrous Na_2SO_4, filtered and concentrated to dryness under reduced pressure. The above crude residue was then dissolved in methanol (5 ml) and a 4 M solution of HCl in dioxane (> 5.0 eq.) was slowly added. The resulting mixture was then heated at 90 °C for 6 hours. After this time, the reaction mixture was allowed to cool down to room temperature, quenched with a 2 M aqueous NaOH-solution and extracted with ethyl acetate (3 x). The combined organic layer was dried over anhydrous Na_2SO_4, filtered and then concentrated to dryness under reduced pressure. The residue was purified by column chromatography on silica gel (dichloromethane / methanol 15:1) to yield the desired product **PIKiN2-R1** as a colorless solid (88.1 mg, 192 µmol, 57% yield).

Compound **PIKiN2-R1**: **^{1}H NMR** (400 MHz, $(CD_3)_2SO$): δ 8.15 (s, 1 H), 6.82 (s, 1 H), 6.72 (br s, 2 H), 5.83 (s, 1 H), 5.13 (t, $^3J_{H,H}$ = 5.3 Hz, 1 H), 3.93 (s, 2 H), 3.79 (d, $^2J_{H,H}$ = 8.7 Hz, 2 H), 3.74 (d, $^2J_{H,H}$ = 8.7 Hz, 2 H), 3.66-3.62 (m, 6 H), 3.62-3.58 (m, 4 H); **^{19}F{^{1}H} NMR** (376 MHz, $(CD_3)_2SO$): δ − 58.7 (s, 3 F); **^{13}C{^{1}H} NMR** (101 MHz, $CDCl_3$): δ 164.1 (s, 1 C), 162.1 (s, 1 C), 161.5 (s, 1 C), 158.9 (s, 1 C), 151.0 (s, 1 C), 138.0 (q, $^2J_{C,F}$ = 32 Hz, 1 C), 124.3 (s, 1 C), 122.7 (q, $^1J_{C,F}$ = 272 Hz, 1 C), 105.3 (q, $^3J_{C,F}$ = 5.5 Hz, 1 C), 91.6 (s, 1 C), 67.1 (s, 2 C), 64.3 (s, 1 C), 54.8 (s, 2 C), 47.5 (s, 1 C), 44.4 (s, 2 C), 41.6 (s, 1 C); **HRMS** (*m/z*): calc. for $C_{19}H_{23}ClF_3N_6O_2$ ($[M + H]^+$): 459.1518; found, 459.1522; **HPLC** (Acclaim-120 C18, CH_3CN:TFA $_{(99.9:0.1)}$ / MeOH:$H_2O_{(10:90)}$ 5:95 for 0.2 min, 5:95 → 100:0 over 10 min, 100:0 for 3 min, 0.5 ml / min, 40 °C, 254 nm): t_R = 5.59 min.

*6-(4-Chloro-6-morpholinopyrimidin-2-yl)-2-oxa-6-azaspiro[3.3]heptane (**13**):*

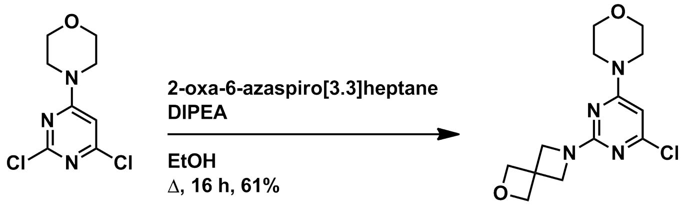

11 **13**

This compound was prepared according to general procedure 3 from 4-(4,6-dichloropyrimidin-2-yl)morpholine (**11**, 1.40 g, 5.98 mmol, 1.0 eq.) and 2-oxa-6-azaspiro[3.3]heptane hemioxalate (2.58 g, 8.95 mmol, 1.5 eq.). After purification by column chromatography on silica gel (cyclohexane / ethyl acetate 1:1), product **13** was obtained as a colorless solid (1.08 g, 3.64 mmol, 61% yield). Crystals suitable for X-ray diffraction were obtained by crystallization from dichloromethane layered with *n*-pentane in an NMR tube.

Compound **13**: ^{1}**H NMR** (400 MHz, $CDCl_3$): δ 5.88 (s, 1 H), 4.82 (s, 4 H), 4.23 (s, 4 H), 3.76-3.71 (m, 4 H), 3.58-3.52 (m, 4 H); ^{13}**C{^{1}H} NMR** (101 MHz, $CDCl_3$): δ 163.5 (s, 1 C), 162.0 (s, 1 C), 160.7 (s, 1 C), 91.7 (s, 1 C), 81.3 (s, 2 C), 66.6 (s, 2 C), 59.7 (s, 2 C), 44.4 (s, 2 C), 38.6 (s, 1 C); **HRMS** (*m/z*): calc. for $C_{13}H_{18}ClN_4O_2$ ($[M + H]^+$): 297.1113; found, 297.1122.

*(1-(4-(6-Amino-4-(trifluoromethyl)pyridin-3-yl)-6-morpholinopyrimidin-2-yl)-3-(chloromethyl)azetidin-3-yl)methanol (**PIKiN2**):*

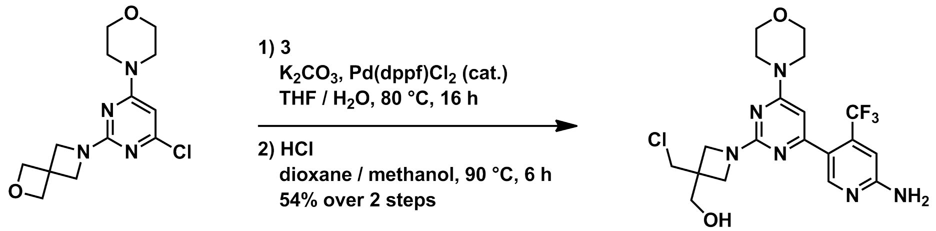

13 **PIKiN2**

This compound was prepared according to general procedure 2 from *N*,*N*-dimethyl-*N'*-(5-(4,4,5,5-tetramethyl-1,3,2-dioxaborolan-2-yl)-4-(trifluoromethyl)pyridin-2-yl)formimidamide (**3**, 138 mg, 402 µmol, 1.1 eq.) and compound **13** (110 mg, 371 µmol, 1.0 eq.). Purification by column chromatography on silica gel (dichloromethane / methanol 15:1) afforded the desired product **PIKiN2** as a colorless solid (92.0 g, 200 µmol, 54% yield).

Compound **PIKiN2**: ^{1}**H NMR** (400 MHz, $CDCl_3$): δ 8.24 (s, 1 H), 6.76 (s, 1 H), 5.97 (s, 1 H), 4.80 (br s, 2 H), 3.93-3.84 (m, 8 H), 3.79-3.74 (m, 4 H), 3.62-3.57 (m, 4 H); ^{19}**F{^{1}H} NMR** (376 MHz, $CDCl_3$): δ − 59.6 (s, 3 F); ^{13}**C{^{1}H} NMR** (101 MHz, $CDCl_3$):

δ 163.0 (s, 1 C), 162.8 (br s, 2 C), 158.7 (s, 1 C), 150.8 (s, 1 C), 137.8 (q, $^2J_{C,F}$ = 32 Hz, 1 C), 124.1 (s, 1 C), 122.8 (q, $^1J_{C,F}$ = 274 Hz, 1 C), 105.0 (q, $^3J_{C,F}$ = 5.3 Hz, 1 C), 93.1 (s, 1 C), 66.6 (s, 2 C), 64.4 (s, 1 C), 55.1 (s, 2 C), 47.7 (s, 1 C), 44.1 (s, 2 C), 40.6 (s, 1 C); **HRMS** (*m/z*): calc. for $C_{19}H_{23}ClF_3N_6O_2$ ([M + H]$^+$): 459.1518; found, 459.1528; **HPLC** (Acclaim-120 C18, CH_3CN:TFA $_{(99.9:0.1)}$ / MeOH:$H_2O_{(10:90)}$ 5:95 for 0.2 min, 5:95 → 100:0 over 10 min, 100:0 for 3 min, 0.5 ml / min, 40 °C, 254 nm): t_R = 5.56 min.

*4-(4-Chloro-6-(piperidin-1-yl)pyrimidin-2-yl)morpholine (**14**):*

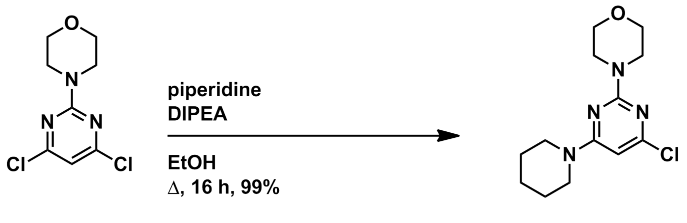

10 14

This compound was prepared according to general procedure 3 from 4-(4,6-dichloropyrimidin-2-yl)morpholine (**10**, 500 mg, 2.14 mmol, 1.0 eq.) and piperidine (240 µL, 2.43 mmol, 1.1 eq.). After purification by column chromatography on silica gel (cyclohexane / ethyl acetate 6:1), compound **14** was obtained as a colorless solid (600 mg, 2.12 mmol, 99% yield).

Compound **14**: **^{1}H NMR** (400 MHz, $CDCl_3$): δ 5.88 (s, 1 H), 3.72 (s, 8 H), 3.57-3.48 (m, 4 H), 1.71-1.62 (m, 2 H), 1.62-1.54 (m, 4 H); **^{13}C{^{1}H} NMR** (101 MHz, $CDCl_3$): δ 163.1 (s, 1 C), 161.2 (s, 1 C), 160.4 (s, 1 C), 91.3 (s, 1 C), 67.0 (s, 4 C), 45.4 (s, 4 C), 44.5 (s, 4 C), 25.7 (s, 4 C), 24.8 (s, 1 C); **HRMS** (*m/z*): calc. for $C_{13}H_{20}N_4OCl$ ([M + H]$^+$): 283.1320; found, 283.1320.

*5-(2-Morpholino-6-(piperidin-1-yl)pyrimidin-4-yl)-4-(trifluoromethyl)pyridin-2-amine (**PIKiN1-R1**):*

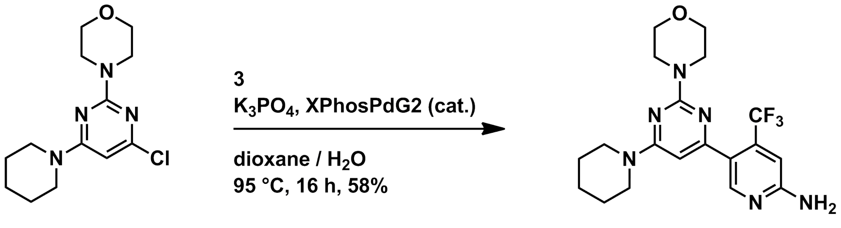

14 PIKiN1-R1

This compound was prepared according to general procedure 1 from 4-(4-chloro-6-(piperidin-1-yl)pyrimidin-2-yl)morpholine (**14**, 40.0 mg, 141 µmol, 1.0 eq.) and *N*,*N*-dimethyl-*N'*-(5-(4,4,5,5-tetramethyl-1,3,2-dioxaborolan-2-yl)-4-(trifluoromethyl)pyridine-2-yl)formimidamide (**3**, 58.0 mg, 169 µmol, 1.2 eq.). Purification by column chromatography on silica gel (dichloromethane / methanol 20:1) gave the desired product **PIKiN1-R1** as a colorless foam (33.3 mg, 81.5 µmol, 58% yield).

Compound **PIKiN1-R1**: **^{1}H NMR** (400 MHz, $CDCl_3$): δ 8.28 (s, 1 H), 6.78 (s, 1 H), 5.99 (s, 1 H), 4.73 (br s, 2H), 3.80-3.71 (m, 8 H), 3.63-3.55 (m, 4 H), 1.71-1.58 (m, 6 H); **^{19}F{^{1}H} NMR** (376 MHz, $CDCl_3$): δ − 59.7 (s, 3 F); **^{13}C{^{1}H} NMR** (101 MHz, $CDCl_3$): δ 162.8 (s, 1 C), 162.4 (s, 1 C), 161.7 (s, 1 C), 158.7 (s, 1 C), 151.0 (s, 1 C), 137.9 (q, $^{2}J_{C,F}$ = 32 Hz, 1 C), 124.8 (s, 1C), 121.5 (q, $^{1}J_{C,F}$ = 275 Hz, 1 C), 105.4 (q, $^{3}J_{C,F}$ = 5.4 Hz, 1 C), 92.8 (s, 1 C), 67.1 (s, 2 C), 45.2 (s, 2 C), 44.5 (s, 2 C), 25.7 (br s, 2 C), 24.9 (s, 1 C); **HRMS** (*m/z*): calc. for $C_{19}H_{24}F_3N_6O$ ([M + H]$^+$): 409.1958; found, 409.1961; **HPLC** (Acclaim-120 C18, CH_3CN:TFA $_{(99.9:0.1)}$ / MeOH:$H_2O_{(10:90)}$ 5:95 for 0.2 min, 5:95 → 100:0 over 10 min, 100:0 for 3 min, 0.5 ml / min, 40 °C, 254 nm): t_R = 6.45 min.

*4-(6-Chloro-2-(piperidin-1-yl)pyrimidin-4-yl)morpholine (**15**):*

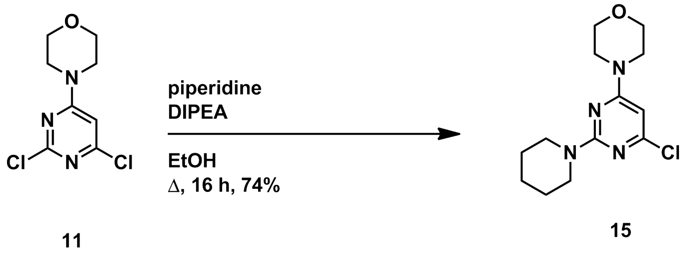

This compound was prepared according to general procedure 3 from 4-(2,6-dichloropyrimidin-4-yl)morpholine (**11**, 750 mg, 3.20 mmol, 1.0 eq.) and piperidine (350 µl, 3.54 mmol, 1.1 eq.). After purification by column chromatography on silica gel (cyclohexane / ethyl acetate 6:1), the desired product **15** was isolated as a colorless solid (670 mg, 2.37 mmol, 74% yield). Crystals suitable for X-ray diffraction were obtained by crystallization from dichloromethane layered with *n*-pentane in an NMR tube.

Compound **15**: **^{1}H NMR** (400 MHz, $CDCl_3$): δ 5.79 (s, 1 H), 3.77-3.68 (m, 8 H), 3.55-3.51 (m, 4 H), 1.68-1.60 (m, 2 H), 1.60-1.52 (m, 4 H); **^{13}C{^{1}H} NMR** (101 MHz, $CDCl_3$): δ 163.8 (s, 1 C), 160.9 (s, 1 C), 160.7 (s, 1 C), 90.2 (s, 1 C), 66.7 (s, 4 C), 45.0 (s, 4 C), 44.5 (s, 4 C), 25.9 (s, 4 C), 25.0 (s, 1 C); **HRMS** (*m/z*): calc. for $C_{13}H_{20}N_4OCl$ ([M + H]$^+$): 283.1320; found, 283.1320.

*5-(6-Morpholino-2-(piperidin-1-yl)pyrimidin-4-yl)-4-(trifluoromethyl)pyridin-2-amine (**PIKiN1**):*

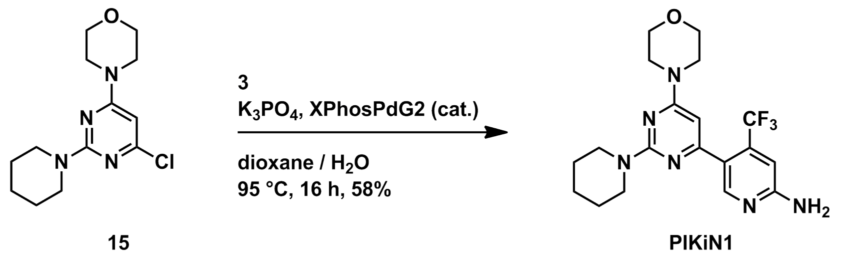

This compound was prepared according to general procedure 1 from 4-(6-chloro-2-(piperidin-1-yl)pyrimidin-4-yl)morpholine (**15**, 40.0 mg, 141 µmol, 1.0 eq.) and *N*,*N*-dimethyl-*N'*-(5-(4,4,5,5-tetramethyl-1,3,2-dioxaborolan-2-yl)-4-(trifluoromethyl)pyridine-2-yl)formimidamide (**3**, 58.0 mg, 169 µmol, 1.2 eq.). Purification by column chromatography on silica gel (dichloromethane / methanol 20:1) gave the desired product **PIKiN1** as a colorless foam (33.2 mg, 81.3 µmol, 58% yield).

Compound **PIKiN1**: **^{1}H NMR** (400 MHz, $CDCl_3$): δ 8.28 (s, 1 H), 6.77 (s, 1 H), 5.89 (s, 1 H), 4.74 (br s, 2H), 3.79-3.74 (m, 8 H), 3.60-3.57 (m, 4 H), 1.64-1.57 (m, 6 H); **^{19}F{^{1}H} NMR** (376 MHz, $CDCl_3$): δ – 59.7 (s, 3 F); **^{13}C{^{1}H} NMR** (101 MHz, $CDCl_3$): δ 163.5 (s, 1 C), 163.0 (s, 1 C), 161.5 (s, 1 C), 158.7 (s, 1 C), 150.9 (s, 1 C), 138.0 (q, $^2J_{C,F}$ = 32 Hz, 1 C), 125.0 (s, 1C), 123.0 (q, $^1J_{C,F}$ = 275 Hz, 1 C), 105.2 (q, $^3J_{C,F}$ = 5.3 Hz, 1 C), 91.5 (s, 1 C), 66.7 (s, 2 C), 44.8 (s, 2 C), 44.3 (s, 2 C), 25.8 (br s, 2 C), 25.0 (s, 1 C); **HRMS** (*m/z*): calc. for $C_{19}H_{24}F_3N_6O$ ($[M + H]^+$): 409.1958; found, 409.1952; **HPLC** (Acclaim-120 C18, CH_3CN:TFA $_{(99.9:0.1)}$ / MeOH:$H_2O_{(10:90)}$ 5:95 for 0.2 min, 5:95 → 100:0 over 10 min, 100:0 for 3 min, 0.5 ml / min, 40 °C, 254 nm): t_R = 6.31 min.

*4-(4-Chloro-6-(pyrrolidin-1-yl)pyrimidin-2-yl)morpholine (**16**):*

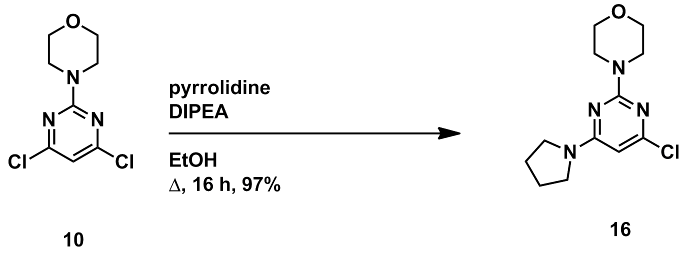

This compound was prepared according to general procedure 3 from dichloropyrimidin-2-yl)morpholine (**10**, 300 mg, 1.28 mmol, 1.0 eq.) and pyrrolidine (120 µl, 1.44 mmol, 1.1 eq.). After purification by column chromatography on silica gel (cyclohexane / ethyl acetate 9:1 → 8:2), compound **16** was obtained as a colorless solid (334 mg, 1.24 mmol, 97% yield).

Compound **16**: **^{1}H NMR** (400 MHz, $CDCl_3$): δ 5.69 (s, 1 H), 3.79-3.68 (m, 8 H), 3.63-3.18 (m, 4 H); 1.96 (br s, 4 H); **^{13}C{^{1}H} NMR** (101 MHz, $CDCl_3$): δ 161.4 (s, 1 C), 161.1 (s, 1 C), 159.3 (s, 1 C), 91.9 (s, 1 C), 67.0 (s, 2 C), 46.4 (br s, 2 C), 44.4 (s, 2 C), 25.3 (s, 2 C); **HRMS** (*m/z*): calc. for $C_{12}H_{18}N_4OCl$ ($[M + H]^+$): 269.1164; found, 269.1163.

*5-(2-Morpholino-6-(pyrrolidin-1-yl)pyrimidin-4-yl)-4-(trifluoromethyl)pyridin-2-amine (**MTD265**):*

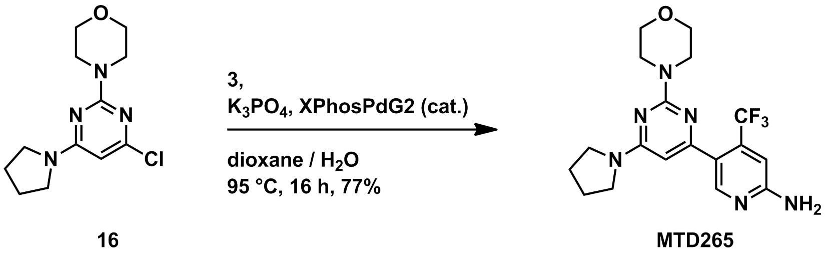

This compound was prepared according to general procedure 1 from 4-(4-chloro-6-(pyrrolidin-1-yl)pyrimidin-2-yl)morpholine (**16**, 98.9 mg, 368 µmol, 1.0 eq.), and *N*,*N*-dimethyl-*N'*-(5-(4,4,5,5-tetramethyl-1,3,2-dioxaborolan-2-yl)-4-(trifluoromethyl)pyridine-2-yl)formimidamide (**3**, 140 mg, 408 µmol, 1.1 eq.). Purification by column chromatography on silica gel (cyclohexane / ethyl acetate 4:6 → 3:7) yielded the desired product **MTD265** as a colorless solid (112 mg, 284 µmol, 77% yield).

Compound **MTD265**: **^{1}H NMR** (400 MHz, $CDCl_3$): δ 8.29 (s, 1 H), 6.78 (s, 1 H), 5.79 (s, 1 H), 4.74 (br s, 2 H), 3.83-3.72 (m, 8 H), 3.68-3.19 (br m, 4 H), 1.98 (br s, 4 H); **^{19}F{^{1}H} NMR** (376 MHz, $CDCl_3$): δ − 59.7 (s, 3 F); **^{13}C{^{1}H} NMR** (101 MHz, $CDCl_3$): δ 161.6 (br s, 2 C), 161.1 (s, 1 C), 158.7 (s, 1 C), 151.1 (s, 1 C), 137.9 (q, $^{2}J_{C,F}$ = 32 Hz, 1 C), 124.8 (s, 1C), 123.0 (q, $^{1}J_{C,F}$ = 275 Hz, 1 C), 105.2 (q, $^{3}J_{C,F}$ = 5.4 Hz, 1 C), 93.6 (s, 1 C), 67.2 (s, 2 C), 46.3 (s, 2 C), 44.5 (s, 2 C), 25.5 (br s, 2 C); **HRMS** (*m/z*): calc. for $C_{18}H_{22}F_3N_6O$ ($[M + H]^+$): 395.1802; found, 395.1797; **HPLC** (Acclaim-120 C18, CH_3CN:TFA$_{(99.9:0.1)}$ / $MeOH:H_2O_{(10:90)}$ 5:95 for 0.2 min, 5:95 → 100:0 over 10 min, 100:0 for 3 min, 0.5 ml / min, 40 °C, 254 nm): t_R = 6.09 min.

*4-(6-Chloro-2-(pyrrolidin-1-yl)pyrimidin-4-yl)morpholine (**17**):*

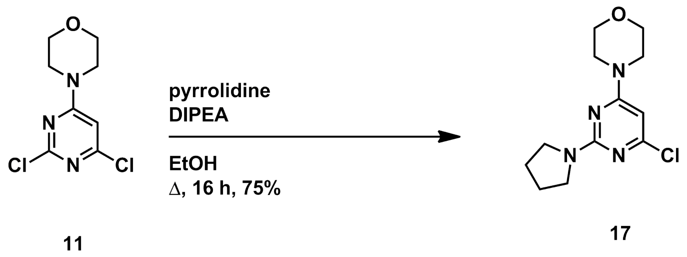

This compound was prepared according to general procedure 3 from 4-(2,6-dichloropyrimidin-4-yl)morpholine (**11**, 300 mg, 1.28 mmol, 1.0 eq.) and pyrrolidine (120 µl, 1.44 mmol, 1.1 eq.). After purification by column chromatography on silica gel (cyclohexane / ethyl acetate 9:1 → 8:2), the desired product **17** was obtained as a colorless solid (257 mg, 956 µmol, 75% yield). Crystals suitable for X-ray diffraction were obtained by crystallization from dichloromethane layered with *n*-pentane in an NMR tube.

Compound **17**: **^{1}H NMR** (400 MHz, $CDCl_3$): δ 5.81 (s, 1 H), 3.77-3.71 (m, 4 H), 3.59-3.47 (m, 8 H); 1.96-1.89 (m, 4 H); **^{13}C{^{1}H} NMR** (101 MHz, $CDCl_3$): δ 163.6 (s, 1 C), 160.4 (s, 1 C), 159.7 (s, 1 C), 90.1 (s, 1 C), 66.7 (s, 2 C), 46.7 (s, 2 C), 44.5 (s, 2 C), 25.6 (s, 2 C); **HRMS** (*m/z*): calc. for $C_{12}H_{18}N_4OCl$ ([M + H]$^+$): 269.1164; found, 269.1165.

*5-(6-Morpholino-2-(pyrrolidin-1-yl)pyrimidin-4-yl)-4-(trifluoromethyl)pyridin-2-amine (**MTD265-R1**):*

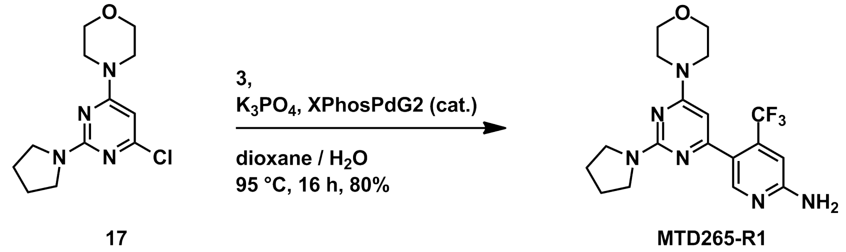

This compound was prepared according to general procedure 1 from 4-(6-chloro-2-(pyrrolidin-1-yl)pyrimidin-4-yl)morpholine (**17**, 100 mg, 372 µmol, 1.0 eq.) and *N*,*N*-dimethyl-*N'*-(5-(4,4,5,5-tetramethyl-1,3,2-dioxaborolan-2-yl)-4-(trifluoromethyl)pyridine-2-yl)formimidamide (**3**, 140 mg, 408 µmol, 1.1 eq.). Purification by column chromatography on silica gel (cyclohexane / ethyl acetate 4:6 → 3:7) afforded the desired product **MTD265-R1** as a colorless solid (117 mg, 297 µmol, 80% yield).

Compound **MTD265-R1**: **^{1}H NMR** (400 MHz, $CDCl_3$): δ 8.28 (s, 1 H), 6.77 (s, 1 H), 5.90 (s, 1 H), 4.74 (br s, 2 H), 3.79-3.75 (m, 4 H), 3.63-3.53 (m, 8 H), 1.97-1.90 (m, 4 H); **^{19}F{^{1}H} NMR** (376 MHz, $CDCl_3$): δ − 59.6 (s, 3 F); **^{13}C{^{1}H} NMR** (101 MHz, $CDCl_3$): δ 163.2 (s, 1 C), 162.9 (s, 1 C), 160.2 (s, 1 C), 158.8 (s, 1 C), 150.9 (s, 1 C), 137.8 (q, $^2J_{C,F}$ = 32 Hz, 1 C), 124.7 (s, 1C), 124.5 (q, $^1J_{C,F}$ = 275 Hz, 1 C), 105.1 (q, $^3J_{C,F}$ = 5.2 Hz, 1 C), 91.6 (s, 1 C), 66.8 (s, 2 C), 46.5 (s, 2 C), 44.3 (s, 2 C), 25.6 (s, 2 C); **HRMS** (*m/z*): calc. for $C_{18}H_{22}F_3N_6O$ ([M + H]$^+$): 395.1802; found, 395.1794; **HPLC** (Acclaim-120 C18, CH_3CN:TFA $_{(99.9:0.1)}$ / MeOH:$H_2O_{(10:90)}$ 5:95 for 0.2 min, 5:95 → 100:0 over 10 min, 100:0 for 3 min, 0.5 ml / min, 40 °C, 254 nm): t_R = 5.87 min.

Synthesis of Intermediates and Final Compounds with Triazine Core:

*4,4'-(6-Chloro-1,3,5-triazine-2,4-diyl)dimorpholine (**19**):*

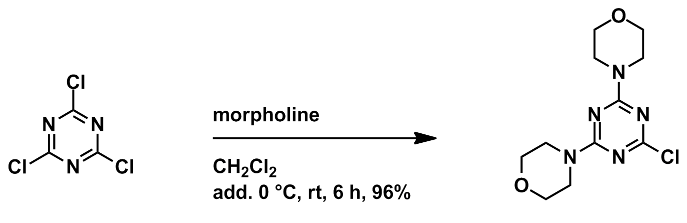

18 **19**

This compound was prepared according to a previously reported literature procedure[3]. Cyanuric chloride (**18**, 20.0 g, 108 mmol, 1.0 eq.) was dissolved in dichloromethane (600 ml) and morpholine (37.9 ml, 434 mmol, 4.0 eq.) was added dropwise at 0 °C in an ice-bath. After the addition was complete, the reaction mixture was further stirred at room temperature for 6 hours. Then, an aqueous saturated $NaHSO_4$-solution was added and the layers were separated. The organic layer was again washed with an aqueous saturated $NaHSO_4$-solution (2 x), dried over anhydrous Na_2SO_4, filtered and then concentrated to dryness under reduced pressure. The desired product **19** was obtained as a colorless solid (29.0 g, 101 mmol, 94%).

Compound **19**: **^{1}H NMR** (400 MHz, $CDCl_3$): δ 3.84-3.73 (br s, 8 H), 3.73-3.66 (m, 8 H); **^{13}C{^{1}H} NMR** (101 MHz, $CDCl_3$): δ 169.8 (s, 1 C), 164.6 (s, 2 C), 66.8 (s, 2 C), 66.7 (s, 2 C), 44.0 (s, 4 C).

*5-(4,6-Dimorpholino-1,3,5-triazin-2-yl)-4-(trifluoromethyl)pyridin-2-amine (**PQR309**):*

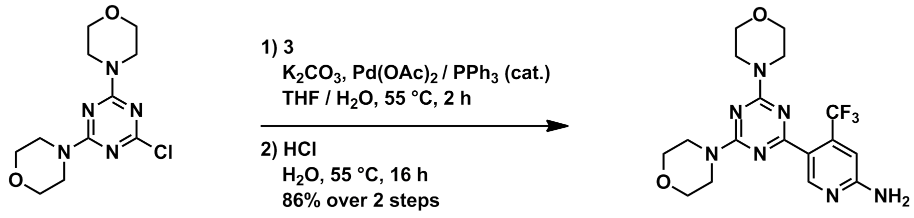

19 **PQR309**

$Pd(OAc)_2$ (18.0 mg, 80.2 µmol, 0.04 eq.) and PPh_3 (63.0 mg, 240 µmol, 0.12 eq.) were dissolved in tetrahydrofuran (6.25 ml) and stirred at room temperature for 1 hour. This solution was then added to a mixture of *N*,*N*-dimethyl-*N'*-(5-(4,4,5,5-tetramethyl-1,3,2-dioxaborolan-2-yl)-4-(trifluoromethyl)pyridin-2-yl)formimidamide (**3**, 823 mg, 2.42 mmol, 1.2 eq.), 4,4'-(6-chloro-1,3,5-triazine-2,4-diyl)dimorpholine (**19**, 572 mg, 2.00 mmol, 1.0 eq.) and aqueous K_2CO_3 (2.4 M, 2.50 ml, 6.00 mmol, 3.0 eq.) in tetrahydrofuran (5 ml). Then, the reaction mixture was heated at 55 °C for 2 hours. After this time, the reaction mixture was allowed to cool down to room temperature and a 5 M aqueous HCl-solution (4.00 ml, 20.0 mmol, 10 eq.) was slowly added. The resulting reaction mixture was then heated again at 55 °C for 16 hours. Upon completion of the

reaction, the mixture was allowed to cool down to room temperature, quenched with a 2 M aqueous NaOH-solution and extracted with ethyl acetate (3 x). The combined organic layer was dried over anhydrous Na_2SO_4, filtered and then concentrated to dryness under reduced pressure. The residue was purified by column chromatography on silica gel (cyclohexane / ethyl acetate 1:3 → 0:1) to afford the desired product **PQR309** (708 mg, 1.72 mmol, 86% yield).

Compound **PQR309**: **^{1}H NMR** (600 MHz, $(CD_3)_2SO$): δ 8.61 (s, 1 H), 6.94 (s, 2 H), 6.83 (s, 1 H), 3.74 (br s, 8 H), 3.66-3.57 (m, 8 H); **^{19}F{^{1}H} NMR** (376 MHz, $(CD_3)_2SO$): δ – 58.8 (s, 3 F); **^{13}C{^{1}H} NMR** (151 MHz, $(CD_3)_2SO$): δ 169.5 (s, 1 C), 164.2 (s, 2 C), 161.2 (s, 1 C), 152.5 (s, 1 C), 136.2 (q, $^2J_{C,F}$ = 32 Hz, 1 C), 123.1 (q, $^1J_{C,F}$ = 274 Hz, 1 C), 118.8 (s, 1 C), 104.7 (q, $^3J_{C,F}$ = 6.0 Hz, 1 C), 66.0 (s, 4 C), 43.2 (s, 4 C); **HRMS** (*m/z*): calc. for $C_{17}H_{21}F_3N_7O_2$ ($[M + H]^+$): 412.1703; found, 412.1699; **HPLC** (Acclaim-120 C18, CH_3CN:TFA $_{(99.9:0.1)}$ / MeOH:$H_2O_{(10:90)}$ 5:95 for 0.2 min, 5:95 → 100:0 over 10 min, 100:0 for 3 min, 0.5 ml / min, 40 °C, 254 nm): t_R = 6.33 min.

*4-(4,6-Dichloro-1,3,5-triazin-2-yl)morpholine (**20**):*

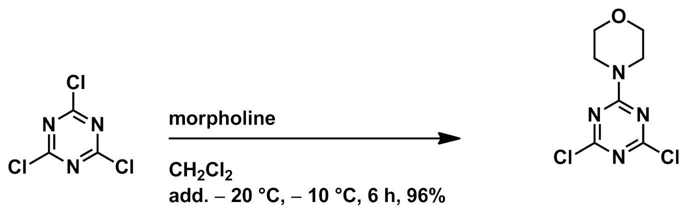

18 20

Cyanuric chloride (**18**, 10.0 g, 54.2 mmol, 1.0 eq.) was dissolved in dichloromethane (200 ml). Morpholine (9.49 ml, 108 mmol, 2.0 eq.) was added dropwise at – 20 °C and the resulting reaction mixture was allowed to stir at – 10 °C for 6 hours. After this time, an aqueous saturated $NaHSO_4$-solution was added and the layers were separated. The organic layer was again washed with an aqueous saturated $NaHSO_4$-solution (2 x), dried over anhydrous Na_2SO_4, filtered and then concentrated to dryness under reduced pressure. Product **20** was obtained as a colorless solid (12.2 g, 51.9 mmol, 96%).

Compound **20**: **^{1}H NMR** (400 MHz, $CDCl_3$): δ 3.90-3.86 (m, 4 H), 3.77-3.72 (m, 4 H); **^{13}C{^{1}H} NMR** (101 MHz, $CDCl_3$): δ 170.5 (s, 2 C), 164.2 (s, 1 C), 66.5 (s, 2 C), 44.6 (s, 2 C). The spectroscopic data are consistent with previous literature reports[4].

*6-(4-Chloro-6-morpholino-1,3,5-triazin-2-yl)-2-oxa-6-azaspiro[3.3]heptane (**21**):*

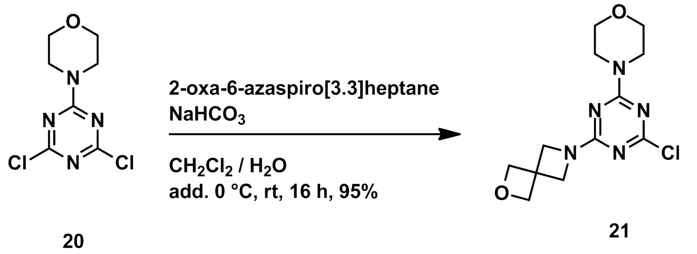

At 0 °C in an ice-bath, was added dropwise an aqueous saturated $NaHCO_3$-solution (150 ml) to a solution of 4-(4,6-dichloro-1,3,5-triazin-2-yl)morpholine (**20**, 4.90 g, 20.8 mmol, 1.0 eq.) and 2-oxa-6-azaspiro[3.3]heptane hemioxalate (6.00 g, 20.8 mmol, 1.5. eq) in dichlororomethane (150 ml). The resulting biphasic mixture was allowed to stir at room temperature for 16 hours. Then, the mixture was diluted with dichloromethane (100 ml) and the layers were separated. The organic layer was washed with an aqueous saturated $NaHSO_4$-solution (2 x), dried over anhydrous Na_2SO_4, filtered and the solvent was evaporated under reduced pressure. The crude product was purified by column chromatography on silica gel (cyclohexane / ethyl acetate 1:3) to afford compound **21** as a colorless solid (5.88 g, 19.7 mmol, 95% yield).

Compound **21**: ^{1}**H NMR** (400 MHz, $CDCl_3$): δ 4.77 (br s, 4 H), 4.30-4.25 (m, 4 H), 3.76-3.69 (m, 4 H), 3.68-3.61 (m, 4 H); ^{13}**C{^{1}H} NMR** (101 MHz, $CDCl_3$): δ 169.4 (s, 1 C), 164.7 (s, 1 C), 164.1 (s, 1 C), 80.8 (s, 1 C), 66.6 (s, 2 C), 59.0 (s, 2 C), 43.8 (s, 2 C), 38.7 (s, 2 C); **HRMS** (*m/z*): calc. for $C_{12}H_{17}ClN_5O_2$ ($[M + H]^+$): 298.1065; found, 298.1074.

*(1-(4-(6-Amino-4-(trifluoromethyl)pyridin-3-yl)-6-morpholino-1,3,5-triazin-2-yl)-3-(chloromethyl)azetidin-3-yl)methanol (**PIKiN3**):*

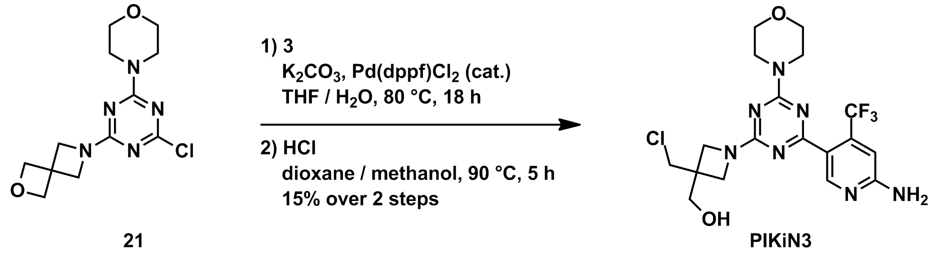

This compound was prepared according to general procedure 2 from *N*,*N*-dimethyl-*N'*-(5-(4,4,5,5-tetramethyl-1,3,2-dioxaborolan-2-yl)-4-(trifluoromethyl)pyridin-2-yl)formimidamide (**3**, 292 mg, 850 µmol, 1.0 eq.) and compound **21** (253 mg, 850 µmol, 1.0 eq.). Purification by column chromatography on silica gel (cyclohexane / ethyl acetate 1:3 → 0:1) afforded product **PIKiN3** as a colorless solid (58.6 mg, 127 µmol, 15% yield).

Compound **PIKiN3**: ^{1}**H NMR** (600 MHz, $(CD_3)_2SO$): δ 8.58 (s, 1 H), 6.93 (br s, 2 H), 6.80 (s, 1 H), 5.11 (t, $^{3}J_{C,F}$ = 5.4 Hz, 1 H), 3.92 (br s, 2 H), 3.87-3.76 (m, 4 H), 3.73 (br s,

4 H), 3.63-3.58 (m, 6 H); **$^{19}F\{^1H\}$ NMR** (376 MHz, $(CD_3)_2SO$): δ − 58.7 (s, 3 F); **$^{13}C\{^1H\}$ NMR** (151 MHz, $(CD_3)_2SO$): δ 169.1 (s, 1 C), 165.1 (s, 1 C), 163.8 (s, 1 C), 161.2 (s, 1 C), 152.4 (s, 1 C), 136.2 (q, $^2J_{C,F}$ = 32 Hz, 1 C), 123.0 (q, $^1J_{C,F}$ = 274 Hz, 1 C), 118.6 (s, 1 C), 104.8-104.5 (m, 1 C), 65.9 (s, 2 C), 62.4 (s, 1 C), 53.8 (s, 1 C), 53.5 (s, 1 C), 48.0 (s, 1 C), 43.1 (s, 2 C), 40.8 (s, 1 C); **HRMS** (*m/z*): calc. for $C_{18}H_{22}ClF_3N_7O_2$ ($[M + H]^+$): 460.1470; found, 460.1475; **HPLC** (Acclaim-120 C18, CH_3CN:TFA $_{(99.9:0.1)}$ / MeOH:$H_2O_{(10:90)}$ 5:95 for 0.2 min, 5:95 → 100:0 over 10 min, 100:0 for 3 min, 0.5 ml / min, 40 °C, 254 nm): t_R = 6.16 min.

^{1}H NMR Spectra

N'-(5-Bromo-4-(trifluoromethyl)pyridin-2-yl)-*N*,*N*-dimethylformimidamide (**2**):

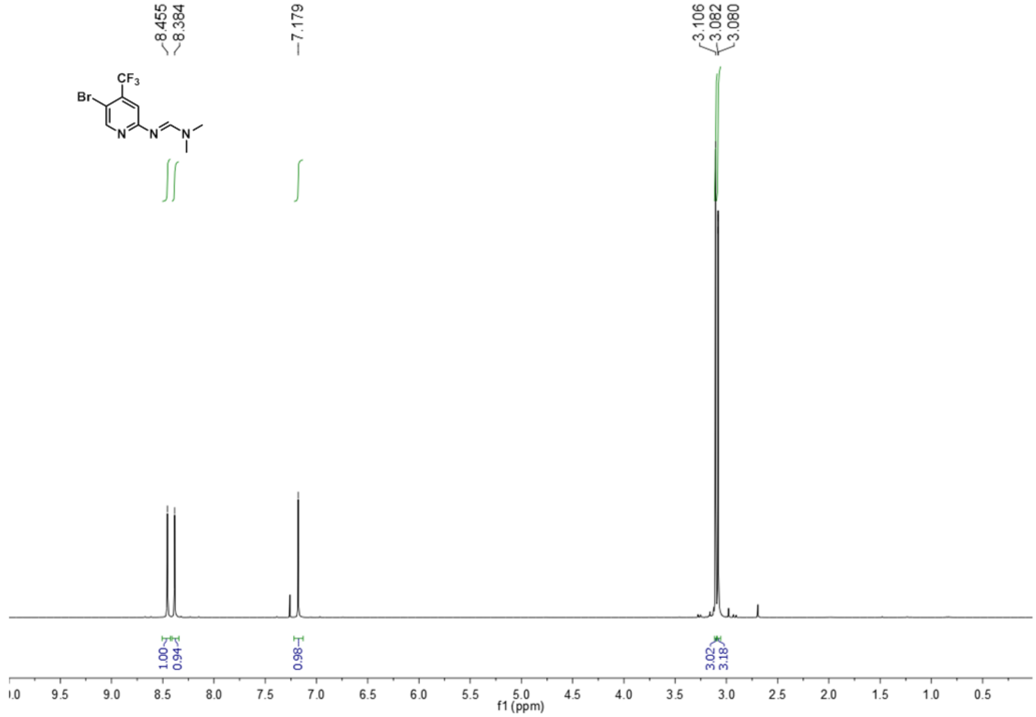

N,*N*-Dimethyl-*N'*-(5-(4,4,5,5-tetramethyl-1,3,2-dioxaborolan-2-yl)-4-(trifluoromethyl)pyridin-2-yl)formimidamide (**3**):

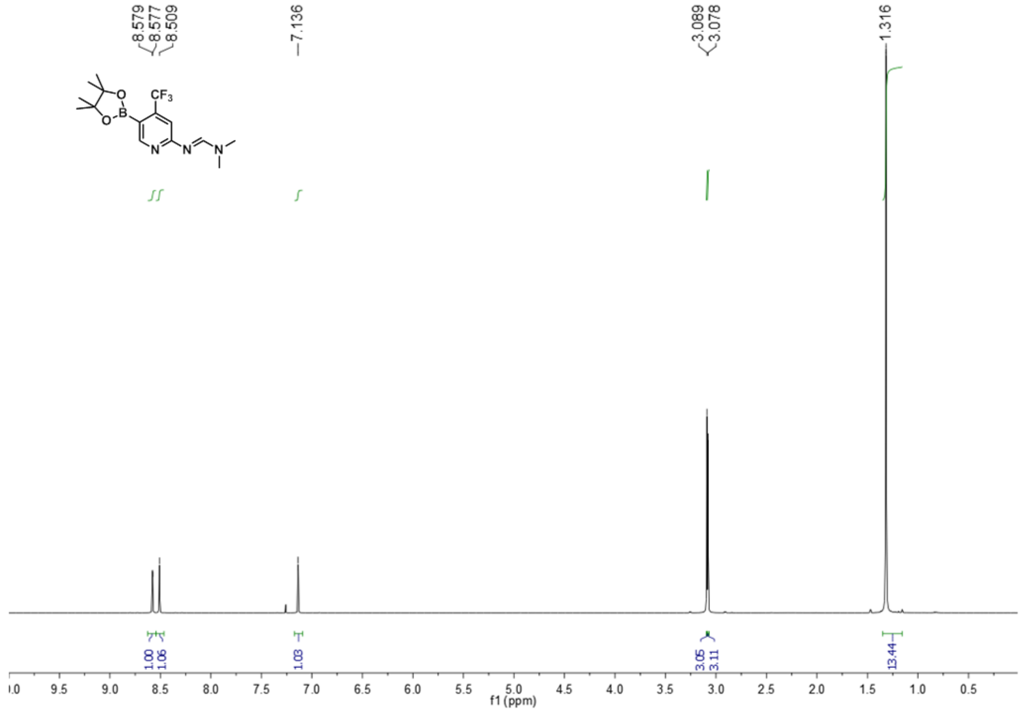

tert-Butyl (5-bromo-4-(trifluoromethyl)pyridin-2-yl)carbamate (**4**):

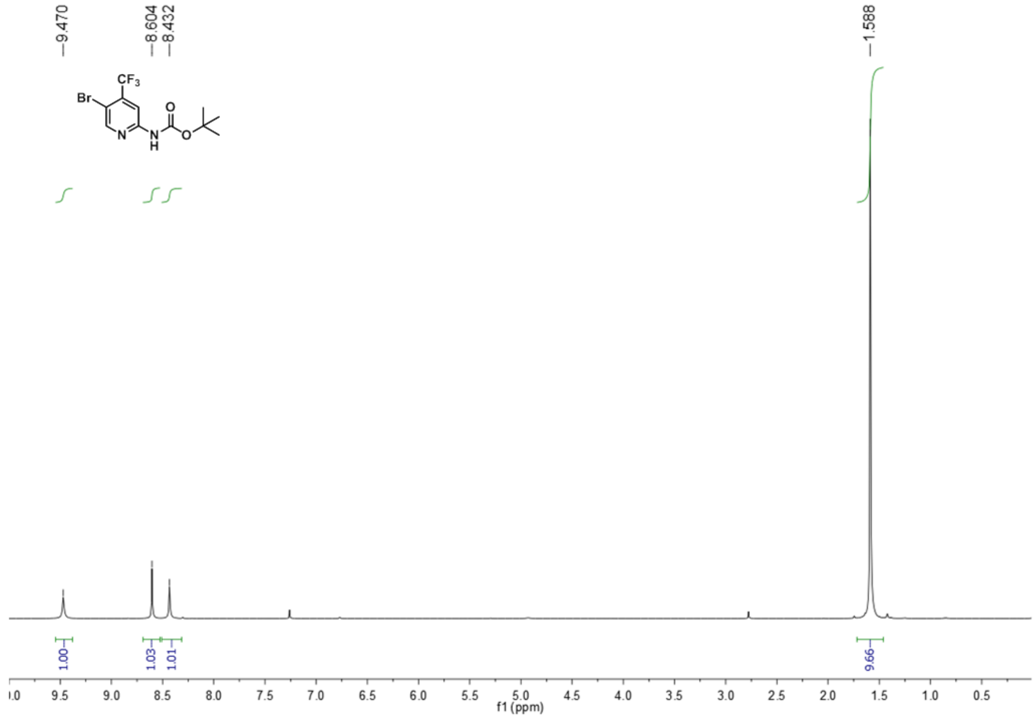

tert-Butyl (2',6'-dichloro-4-(trifluoromethyl)-(3,4'-bipyridin)-6-yl)carbamate (**6**):

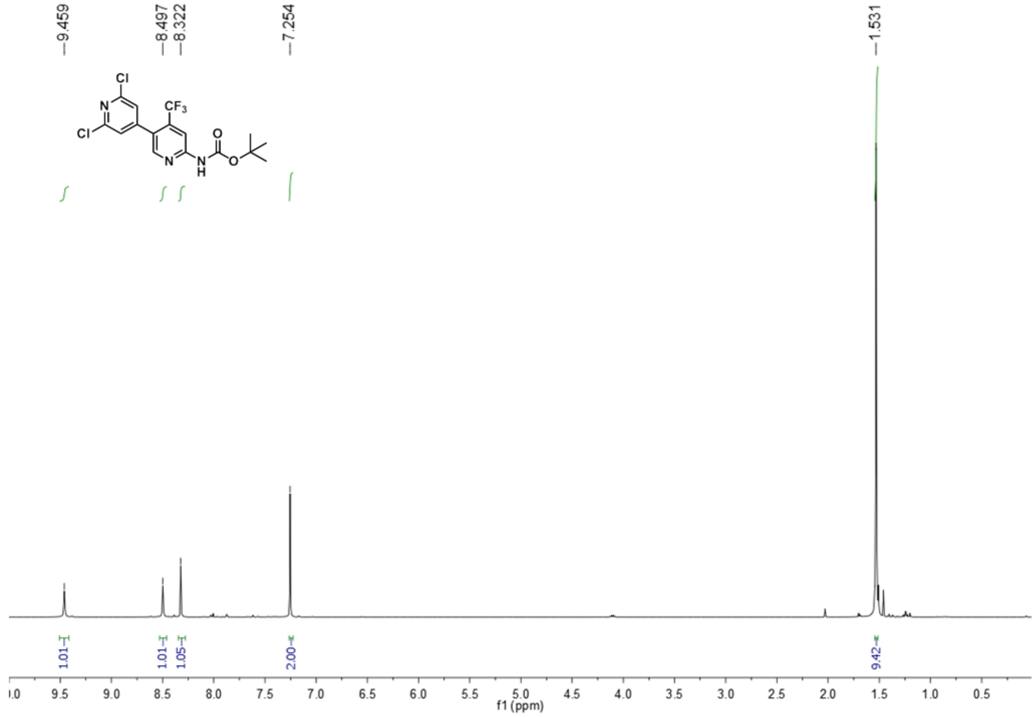

2',6'-Dimorpholino-4-(trifluoromethyl)-(3,4'-bipyridin)-6-amine (**MTD147**):

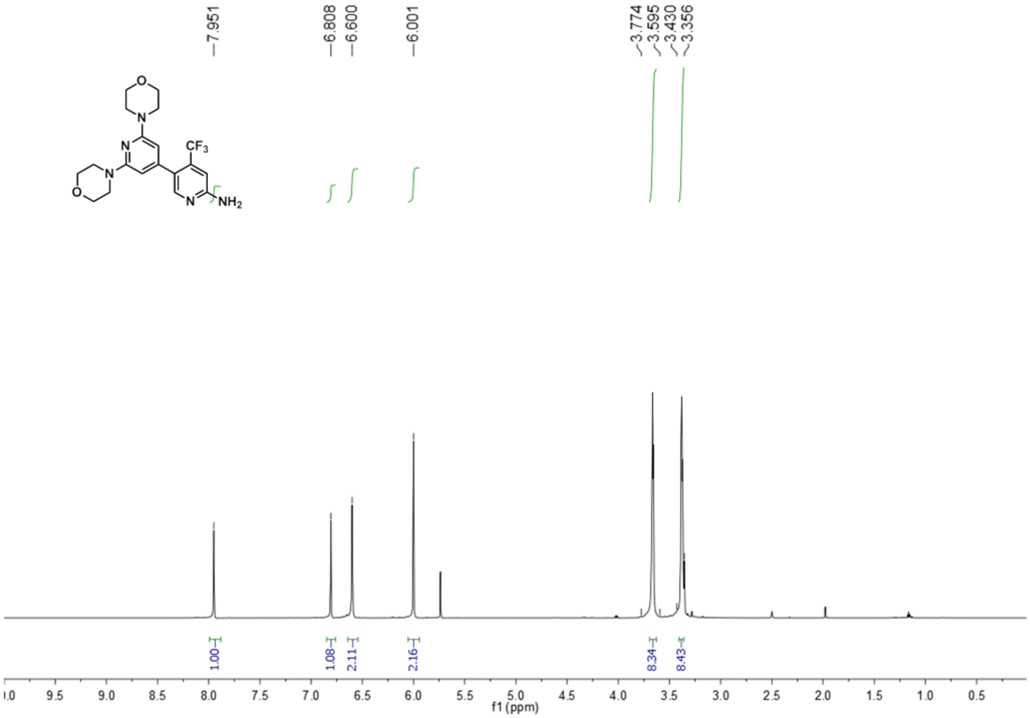

4,4'-(6-Chloropyrimidine-2,4-diyl)dimorpholine (**8**):

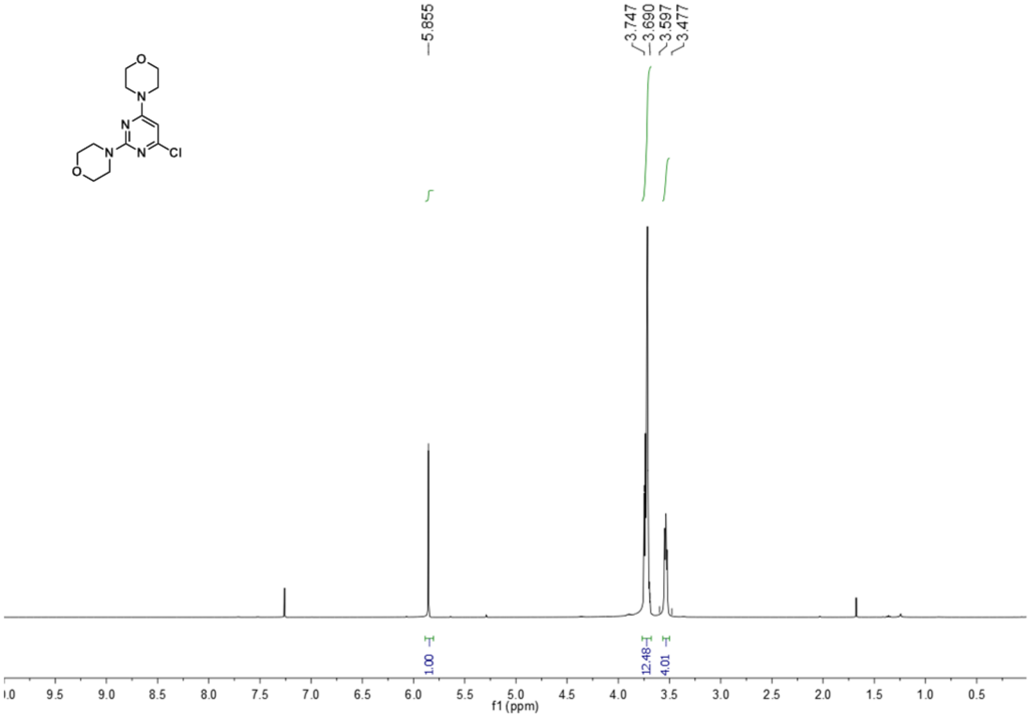

4,4'-(2-Chloropyrimidine-4,6-diyl)dimorpholine (**9**):

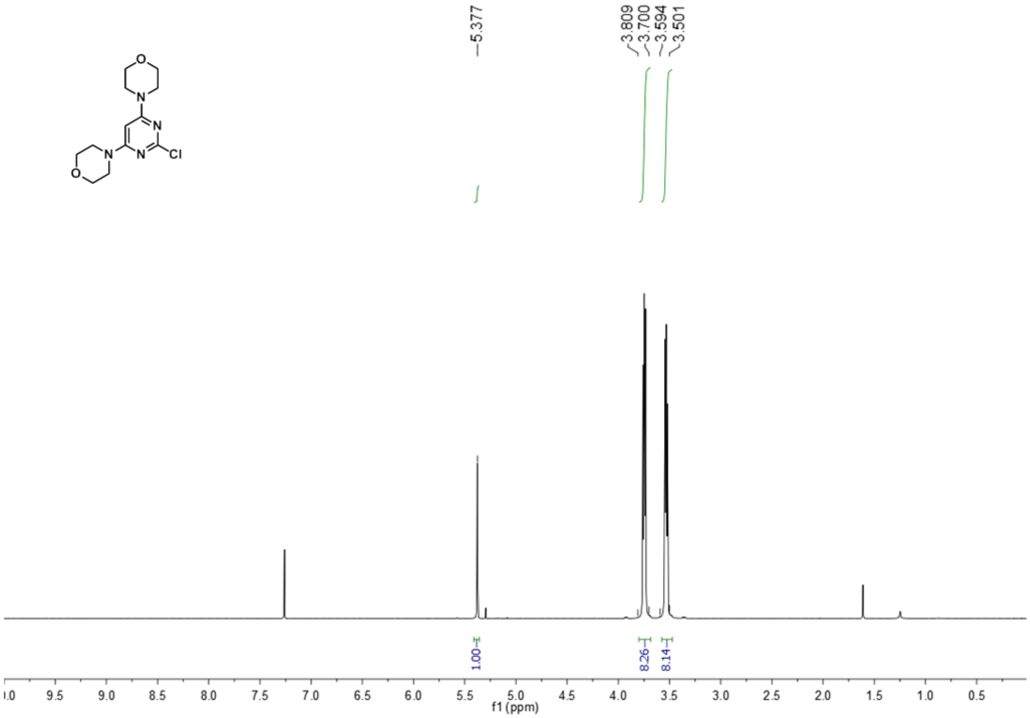

5-(2,6-Dimorpholinopyrimidin-4-yl)-4-(trifluoromethyl)pyridin-2-amine (**BKM120**):

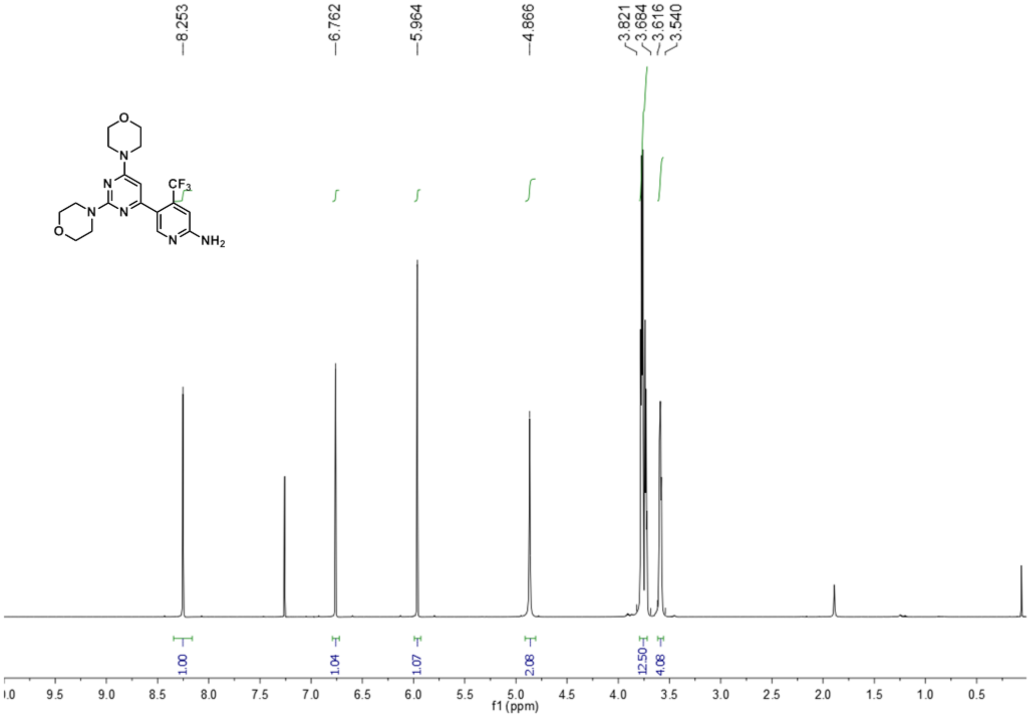

5-(4,6-Dimorpholinopyrimidin-2-yl)-4-(trifluoromethyl)pyridin-2-amine (**BKM120-R1**):

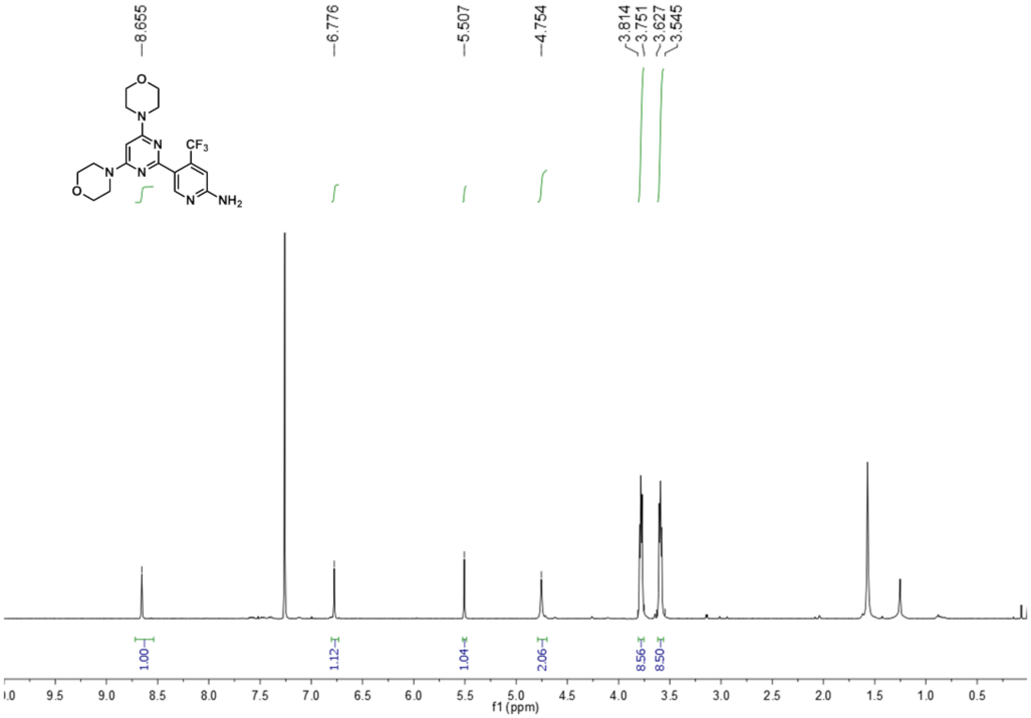

4-(4,6-Dichloropyrimidin-2-yl)morpholine (**10**):

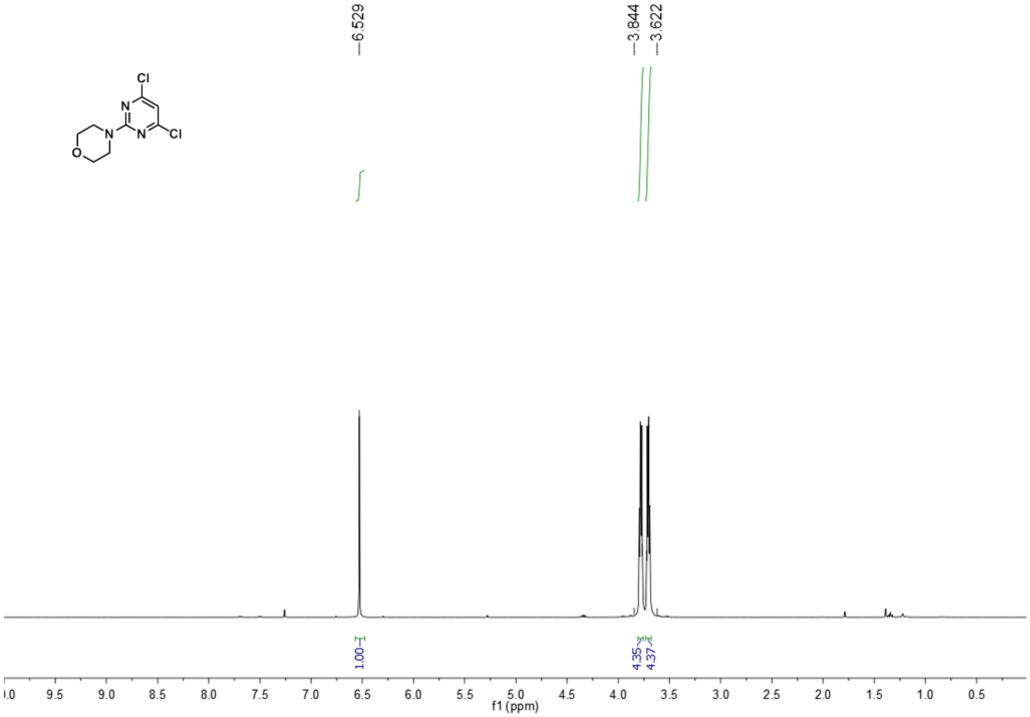

4-(2,6-Dichloropyrimidin-4-yl)morpholine (**11**):

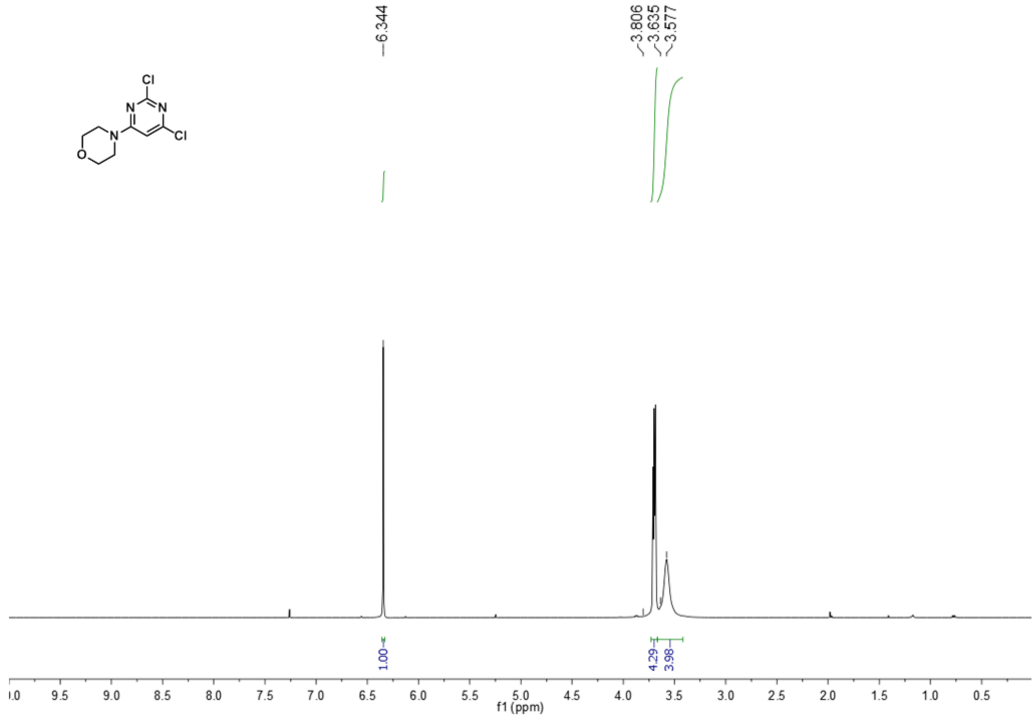

6-(6-Chloro-2-morpholinopyrimidin-4-yl)-2-oxa-6-azaspiro[3.3]heptane (**12**):

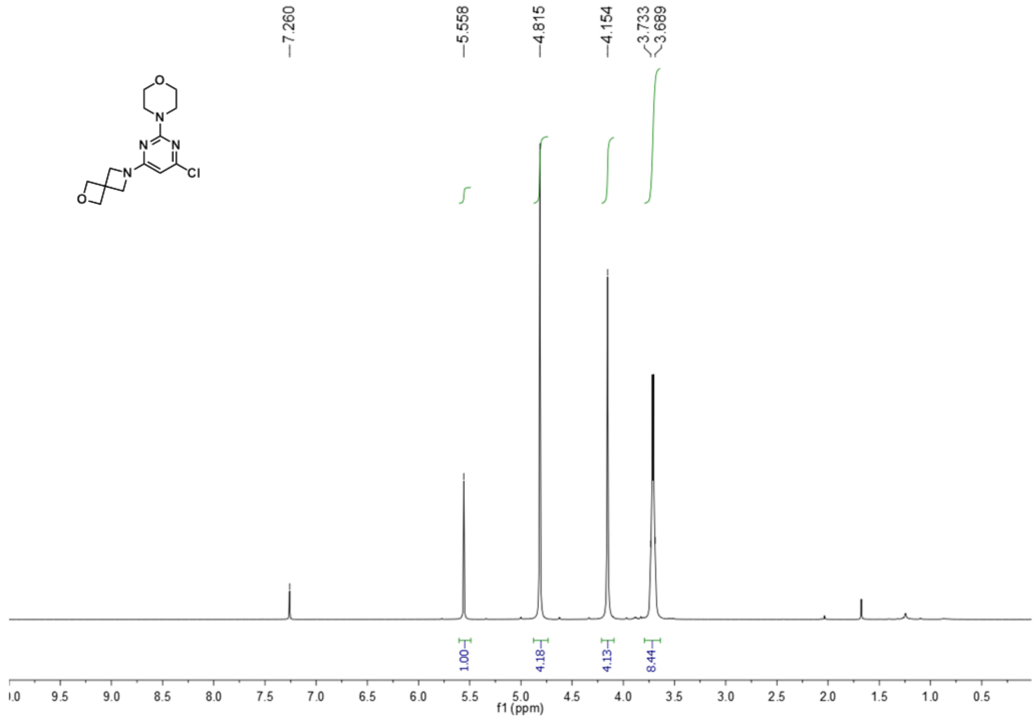

(1-(6-(6-Amino-4-(trifluoromethyl)pyridin-3-yl)-2-morpholinopyrimidin-4-yl)-3-(chloromethyl)azetidin-3-yl)methanol (**PIKiN2-R1**):

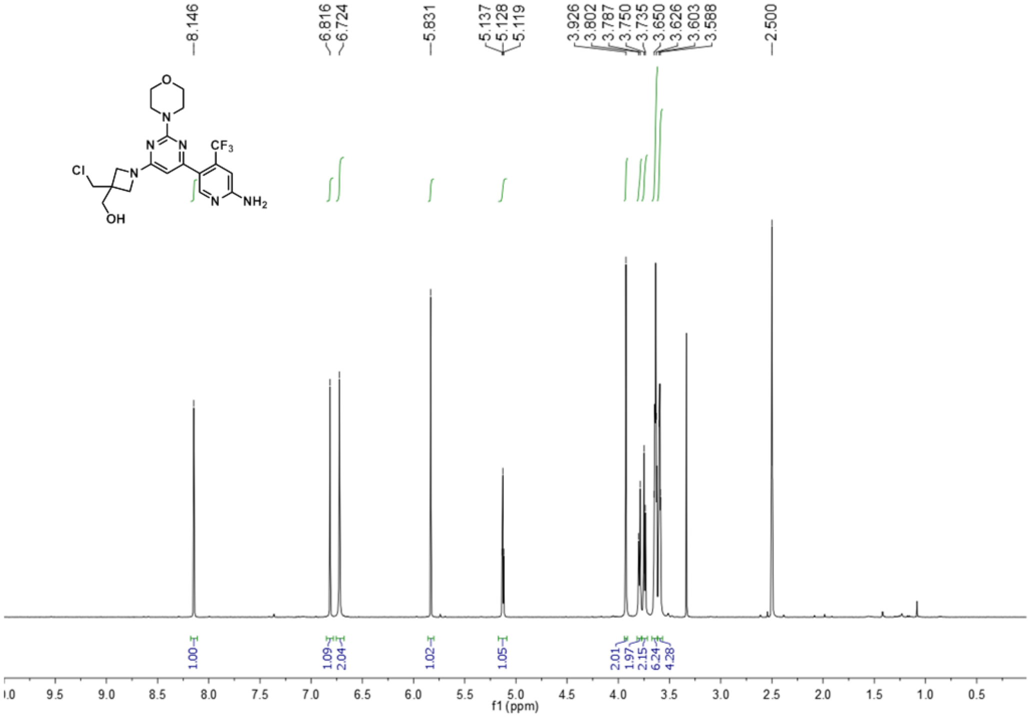

6-(4-Chloro-6-morpholinopyrimidin-2-yl)-2-oxa-6-azaspiro[3.3]heptane (**13**):

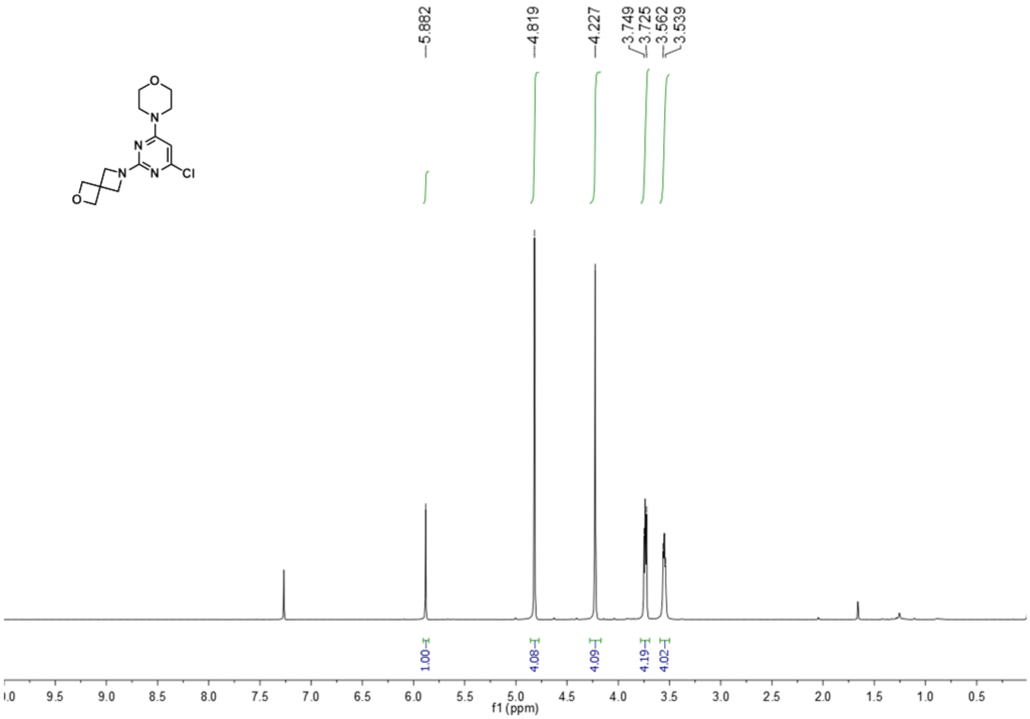

(1-(4-(6-Amino-4-(trifluoromethyl)pyridin-3-yl)-6-morpholinopyrimidin-2-yl)-3-(chloromethyl)azetidin-3-yl)methanol (**PIKiN2**):

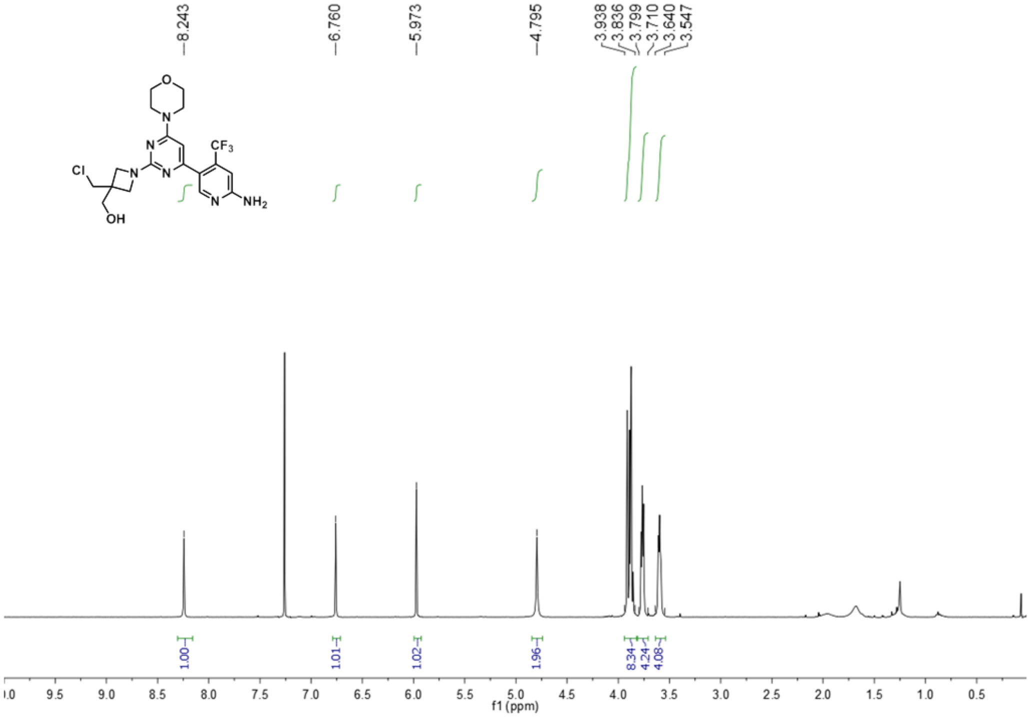

4-(4-Chloro-6-(piperidin-1-yl)pyrimidin-2-yl)morpholine (**14**):

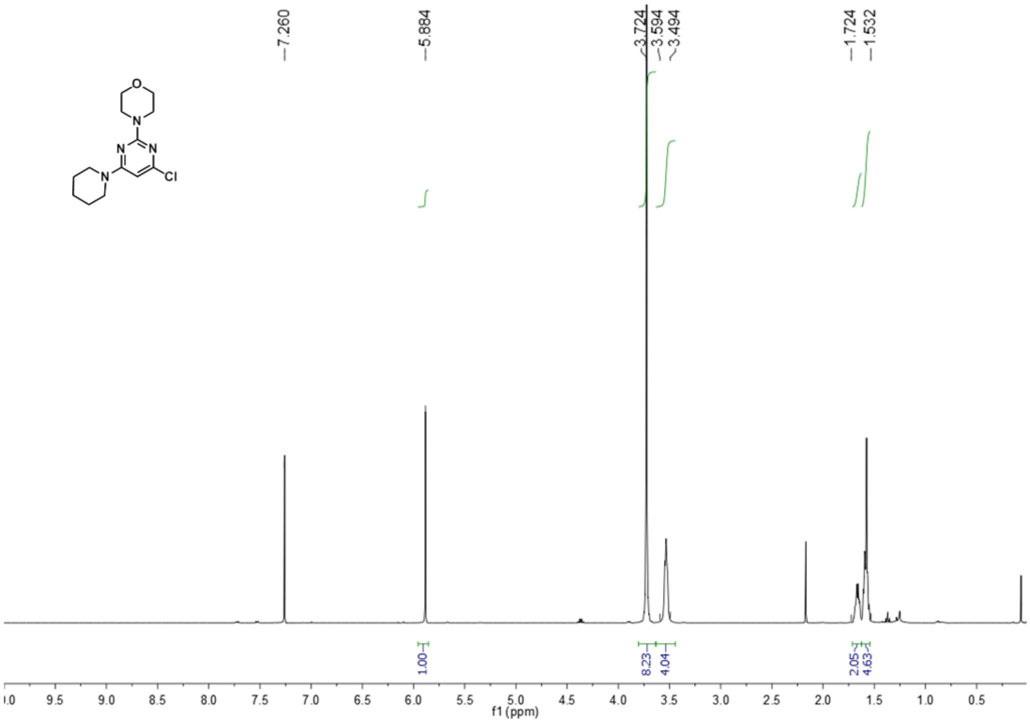

5-(2-Morpholino-6-(piperidin-1-yl)pyrimidin-4-yl)-4-(trifluoromethyl)pyridin-2-amine (**PIKiN1-R1**):

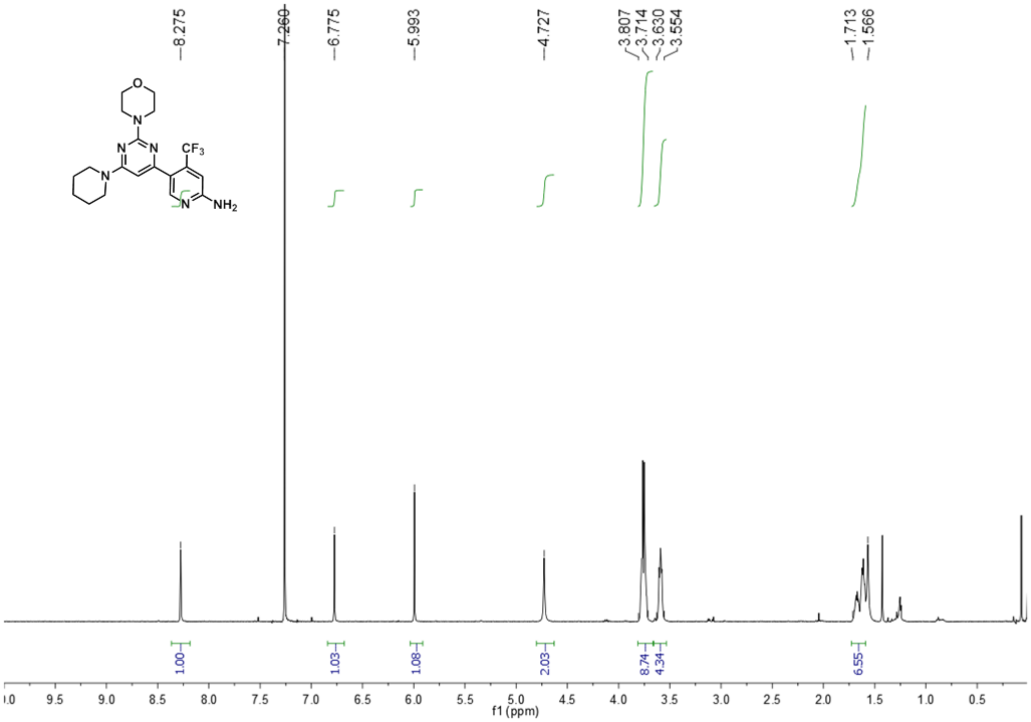

4-(6-Chloro-2-(piperidin-1-yl)pyrimidin-4-yl)morpholine (**15**):

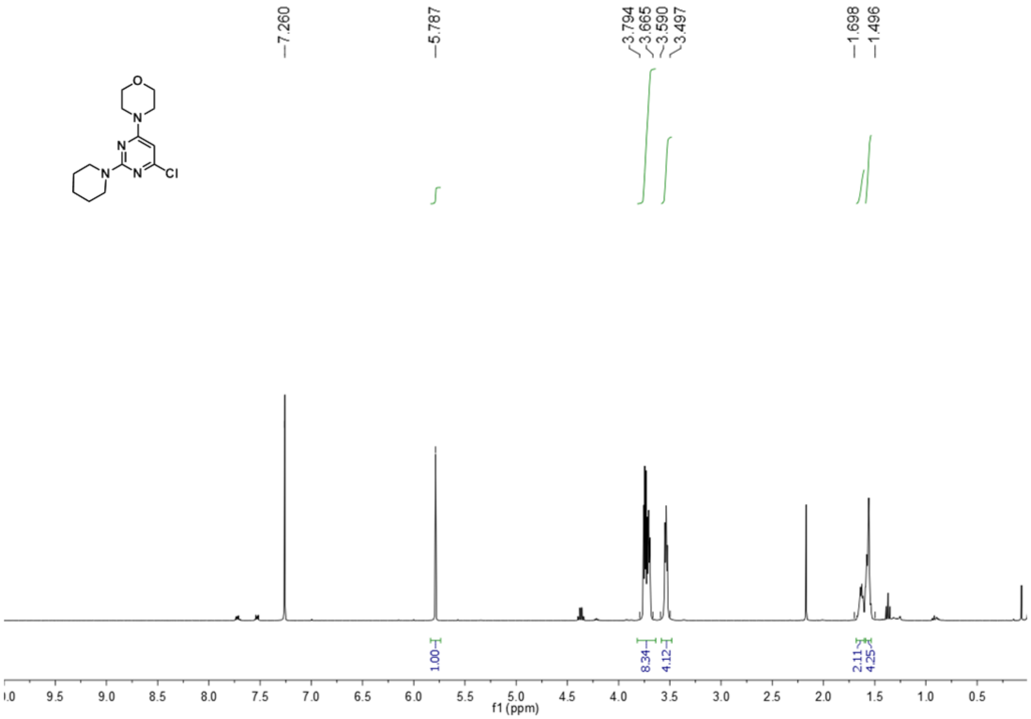

5-(6-Morpholino-2-(piperidin-1-yl)pyrimidin-4-yl)-4-(trifluoromethyl)pyridin-2-amine (**PIKiN1**):

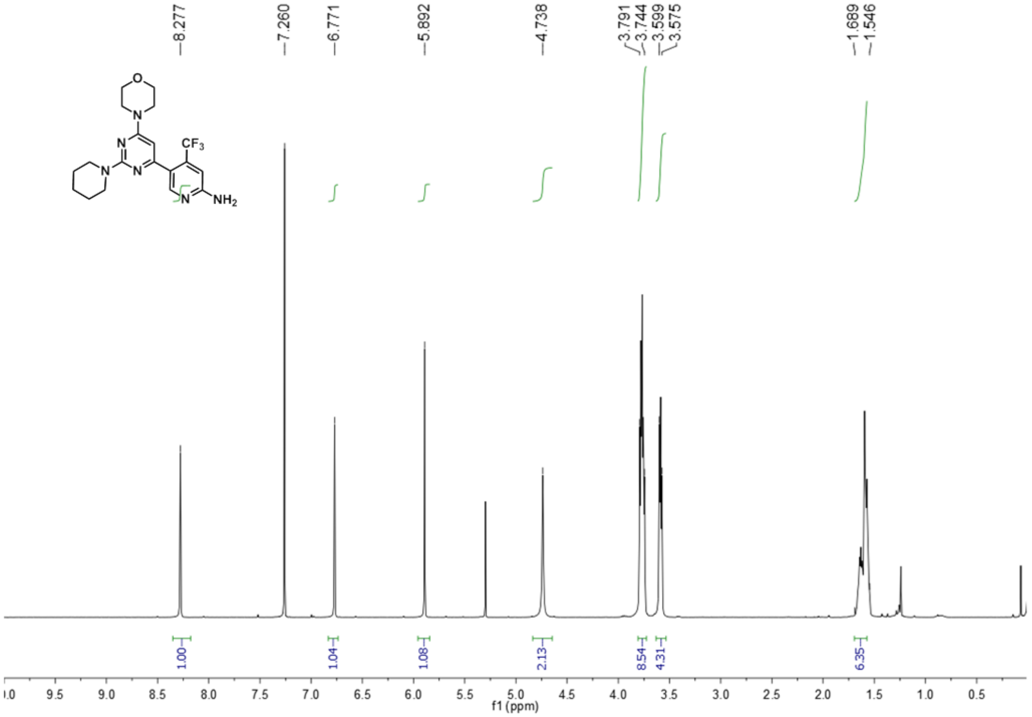

4-(4-Chloro-6-(pyrrolidin-1-yl)pyrimidin-2-yl)morpholine (**16**):

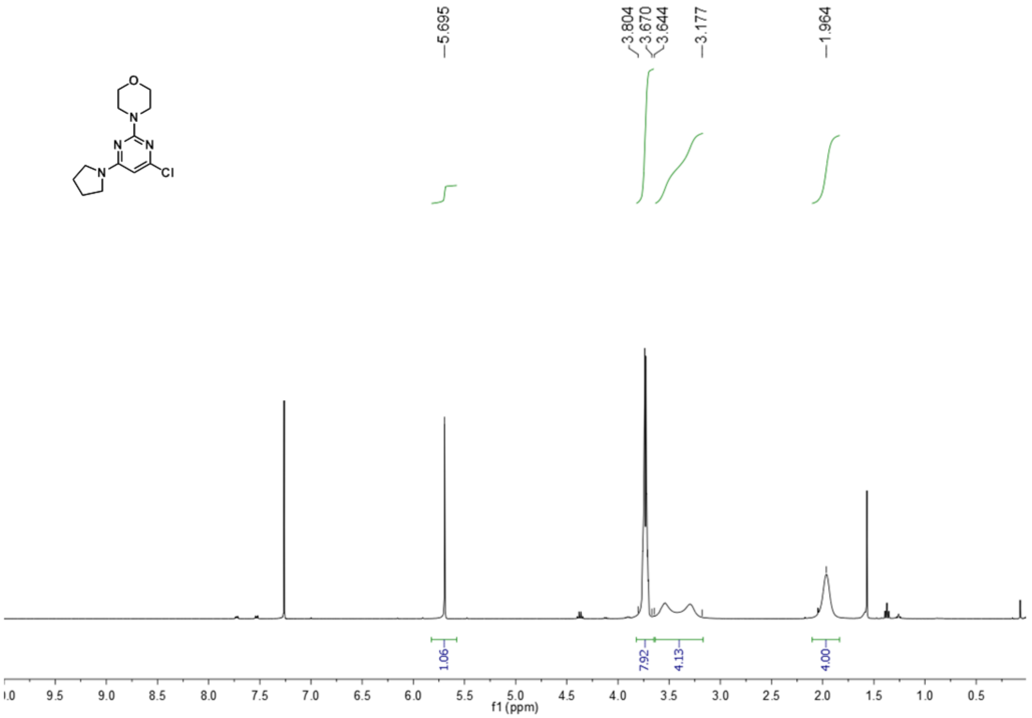

5-(2-Morpholino-6-(pyrrolidin-1-yl)pyrimidin-4-yl)-4-(trifluoromethyl)pyridin-2-amine (**MTD265**):

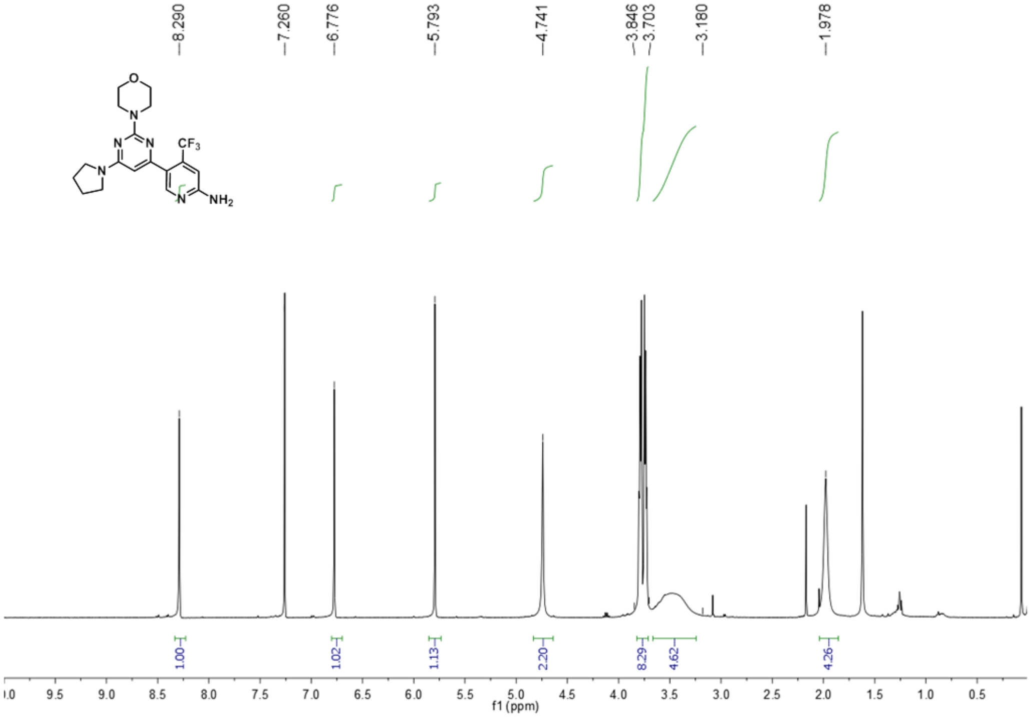

4-(6-Chloro-2-(pyrrolidin-1-yl)pyrimidin-4-yl)morpholine (**17**):

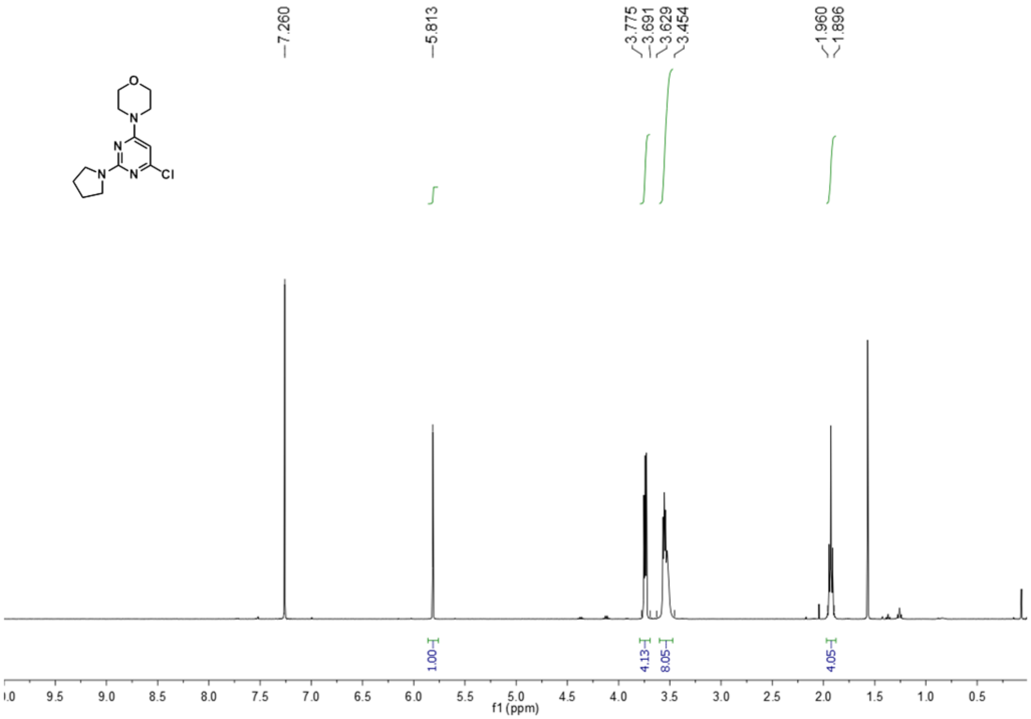

5-(6-Morpholino-2-(pyrrolidin-1-yl)pyrimidin-4-yl)-4-(trifluoromethyl)pyridin-2-amine (**MTD265-R1**):

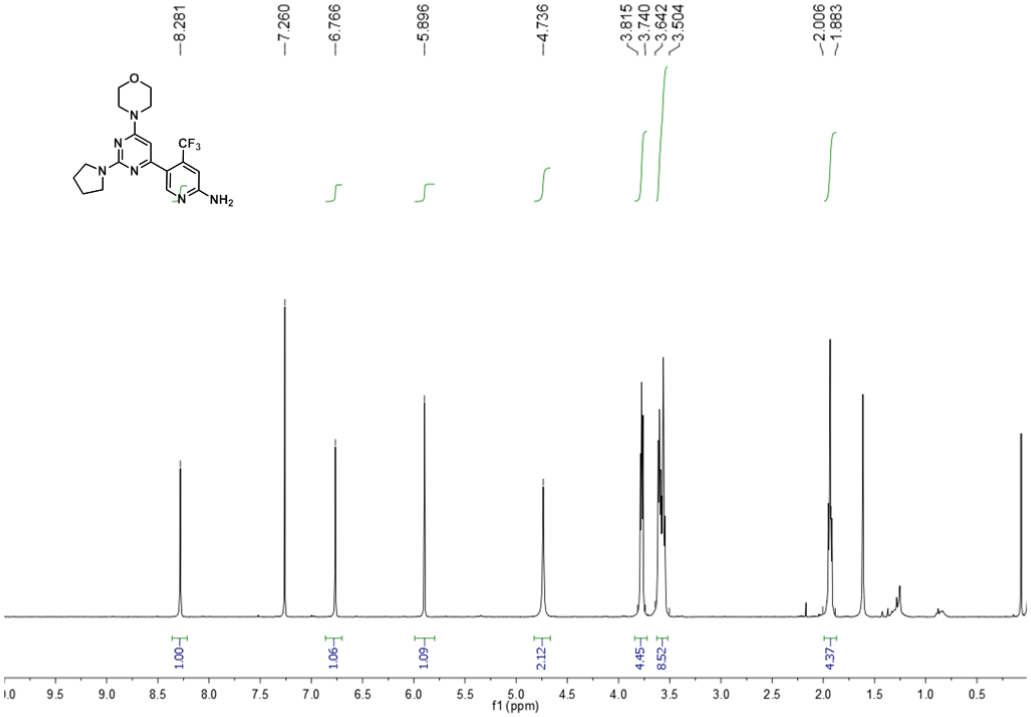

4,4'-(6-Chloro-1,3,5-triazine-2,4-diyl)dimorpholine (**19**):

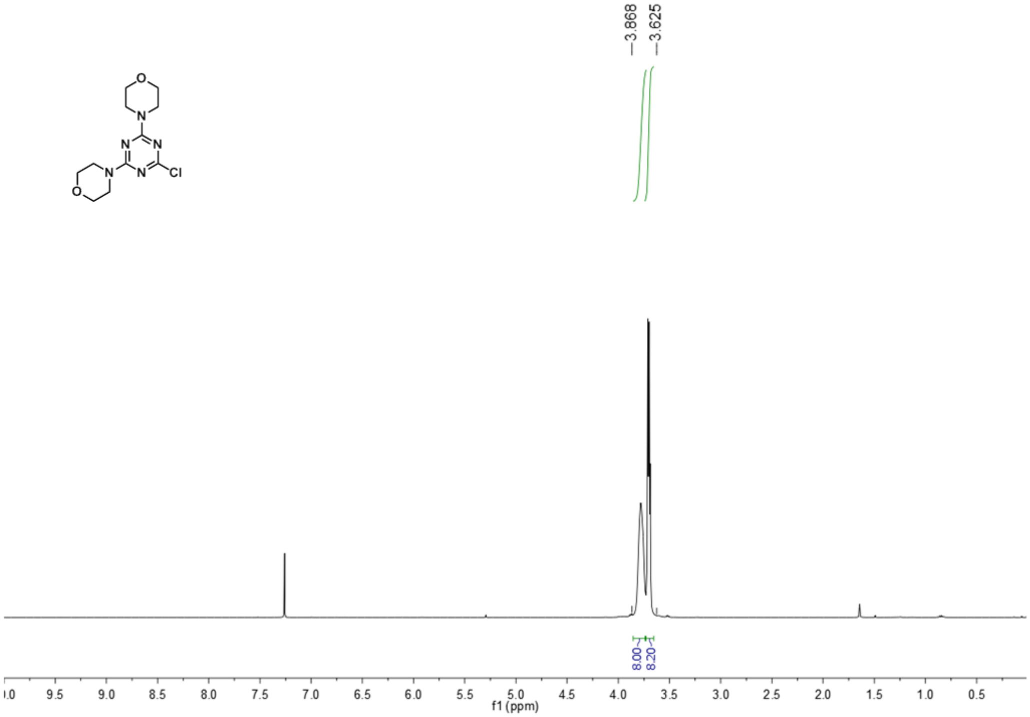

5-(4,6-Dimorpholino-1,3,5-triazin-2-yl)-4-(trifluoromethyl)pyridin-2-amine (**PQR309**):

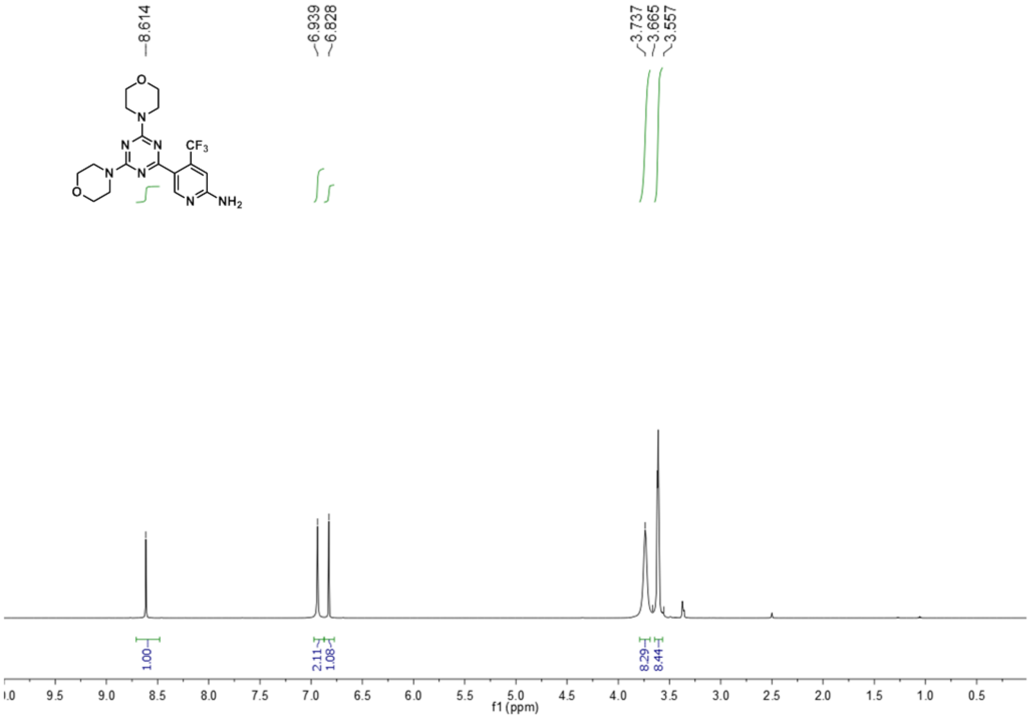

4-(4,6-Dichloro-1,3,5-triazin-2-yl)morpholine (**20**):

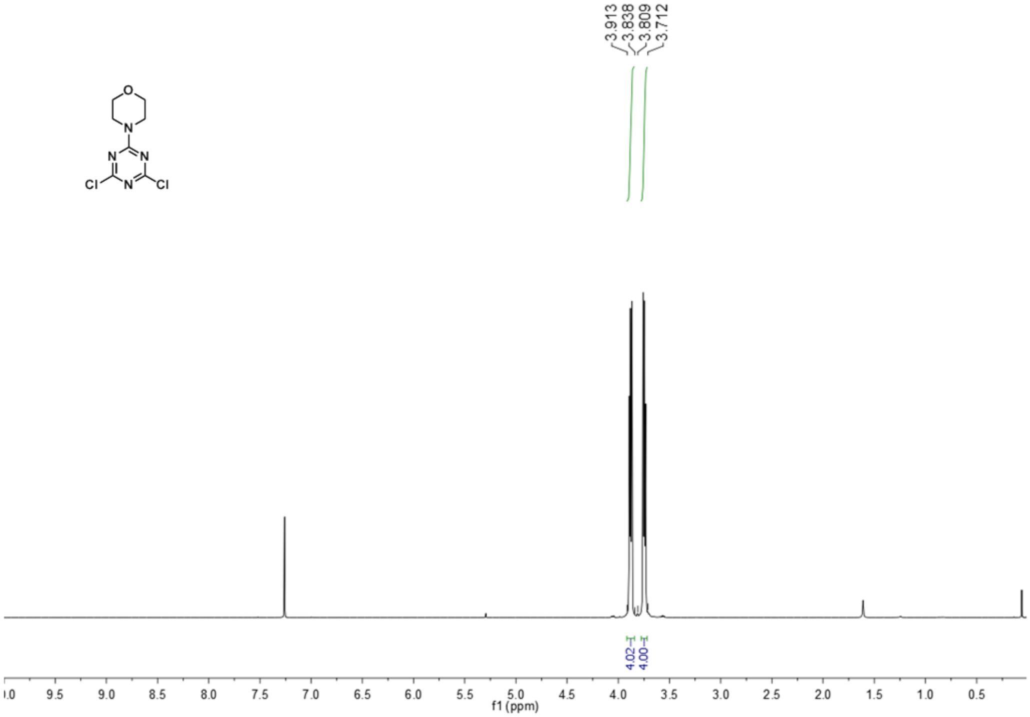

6-(4-Chloro-6-morpholino-1,3,5-triazin-2-yl)-2-oxa-6-azaspiro[3.3]heptane (**21**):

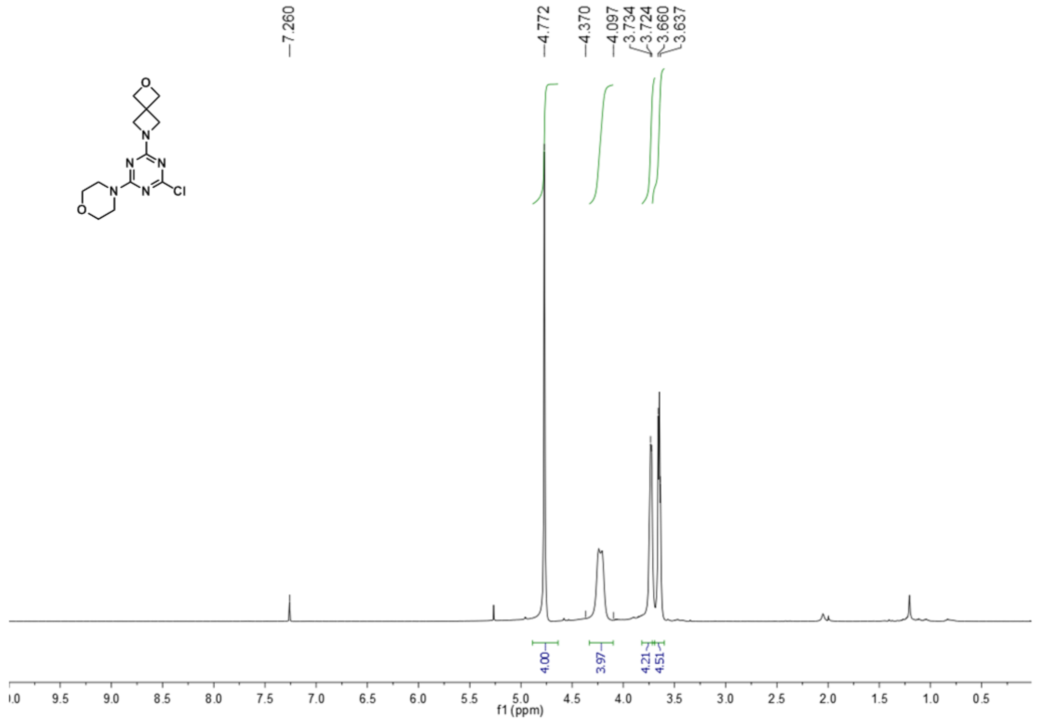

(1-(4-(6-Amino-4-(trifluoromethyl)pyridin-3-yl)-6-morpholino-1,3,5-triazin-2-yl)-3-(chloromethyl)azetidin-3-yl)methanol (**PIKiN3**):

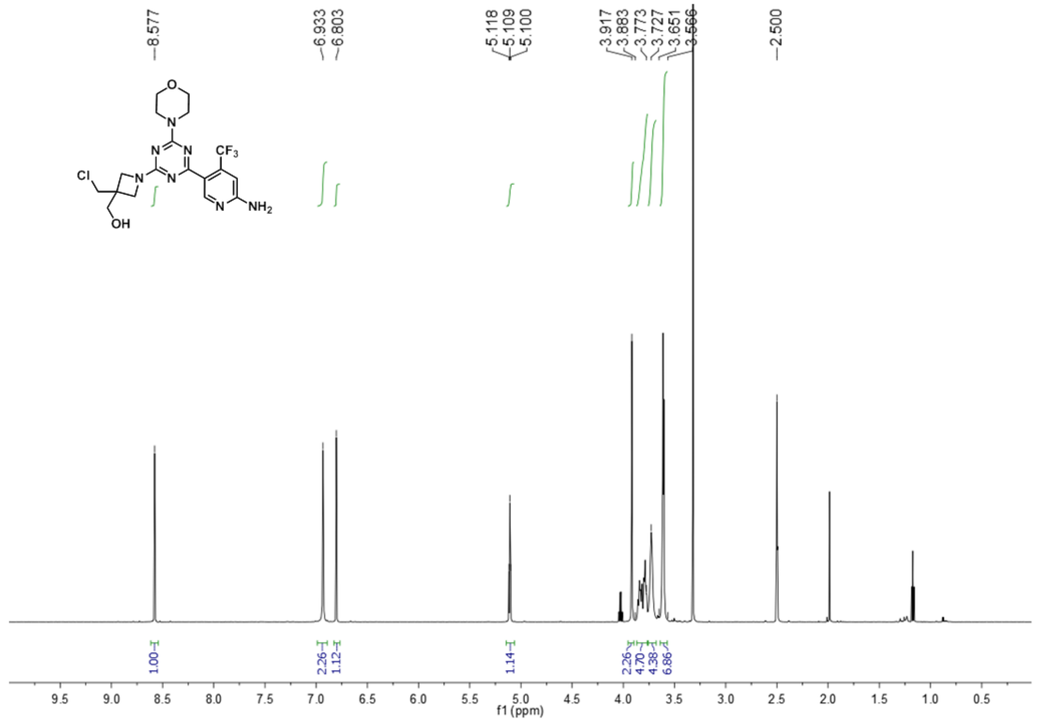

$^{19}F\{^{1}H\}$ NMR Spectra:

N′-(5-Bromo-4-(trifluoromethyl)pyridin-2-yl)-*N*,*N*-dimethylformimidamide (**2**):

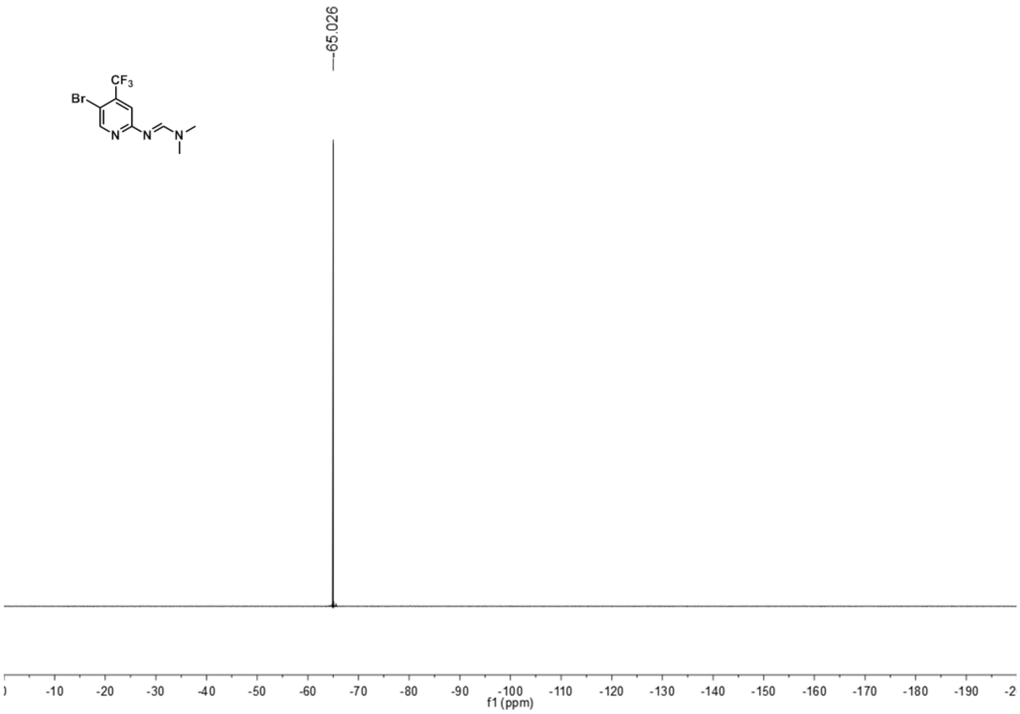

N,*N*-Dimethyl-*N′*-(5-(4,4,5,5-tetramethyl-1,3,2-dioxaborolan-2-yl)-4-(trifluoromethyl)pyridin-2-yl)formimidamide (**3**):

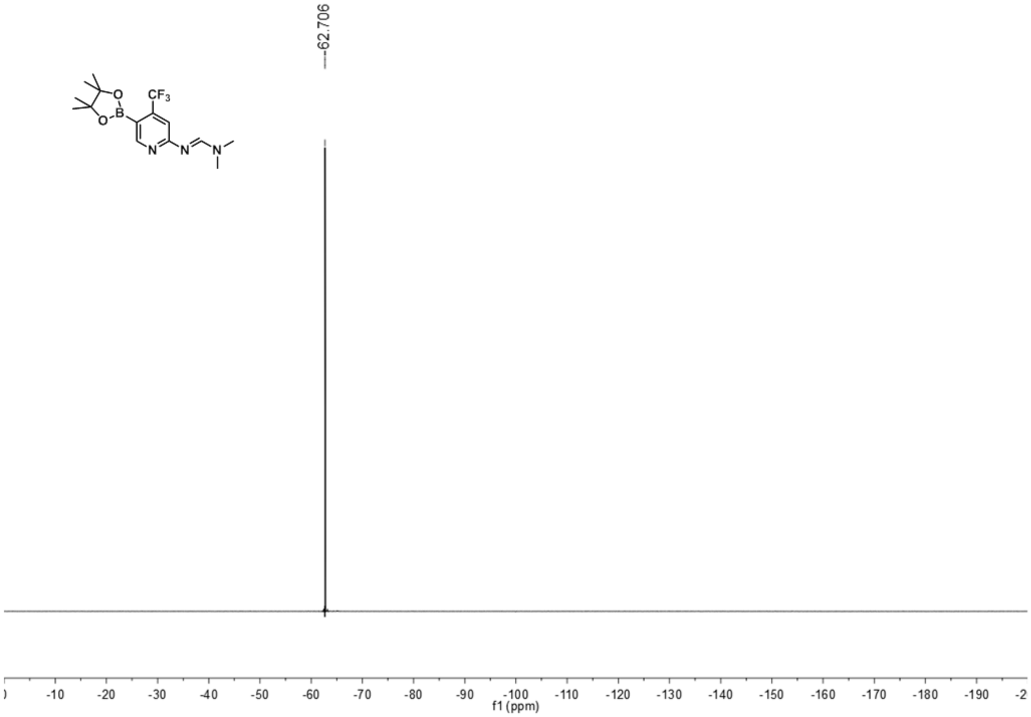

tert-Butyl (5-bromo-4-(trifluoromethyl)pyridin-2-yl)carbamate (**4**):

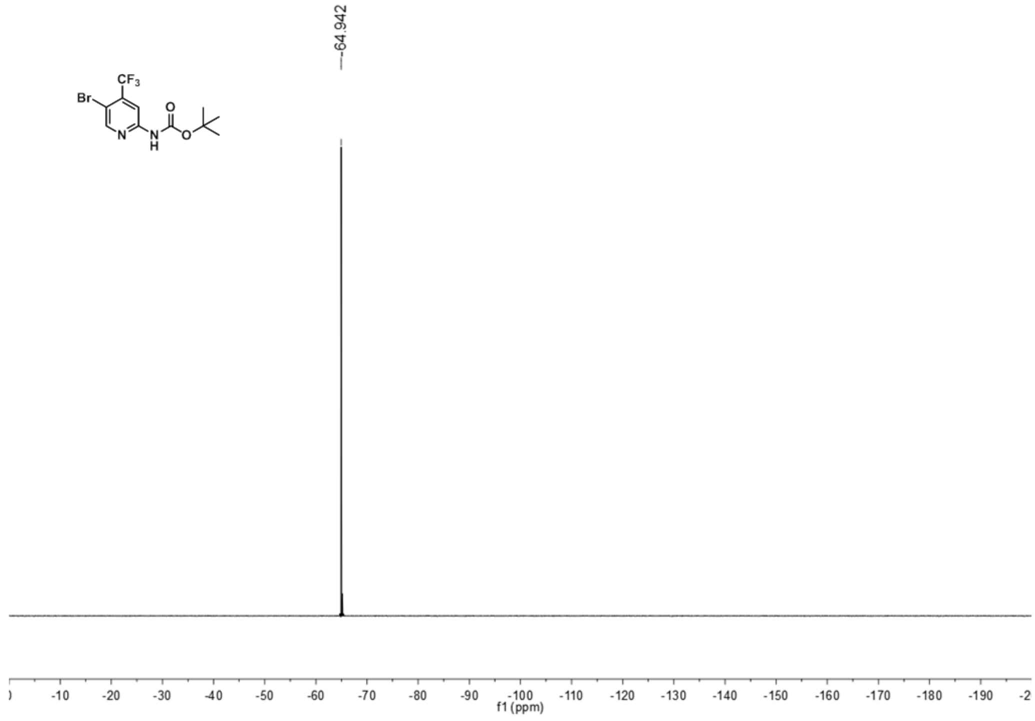

tert-Butyl (2',6'-dichloro-4-(trifluoromethyl)-(3,4'-bipyridin)-6-yl)carbamate (**6**):

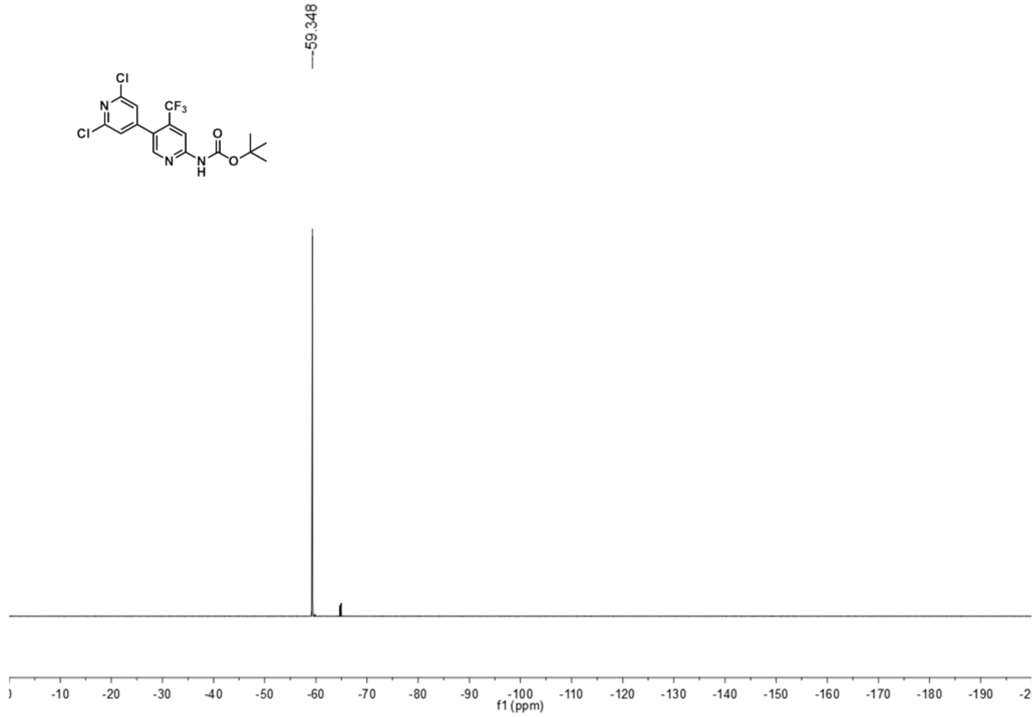

2',6'-Dimorpholino-4-(trifluoromethyl)-(3,4'-bipyridin)-6-amine (**MTD147**):

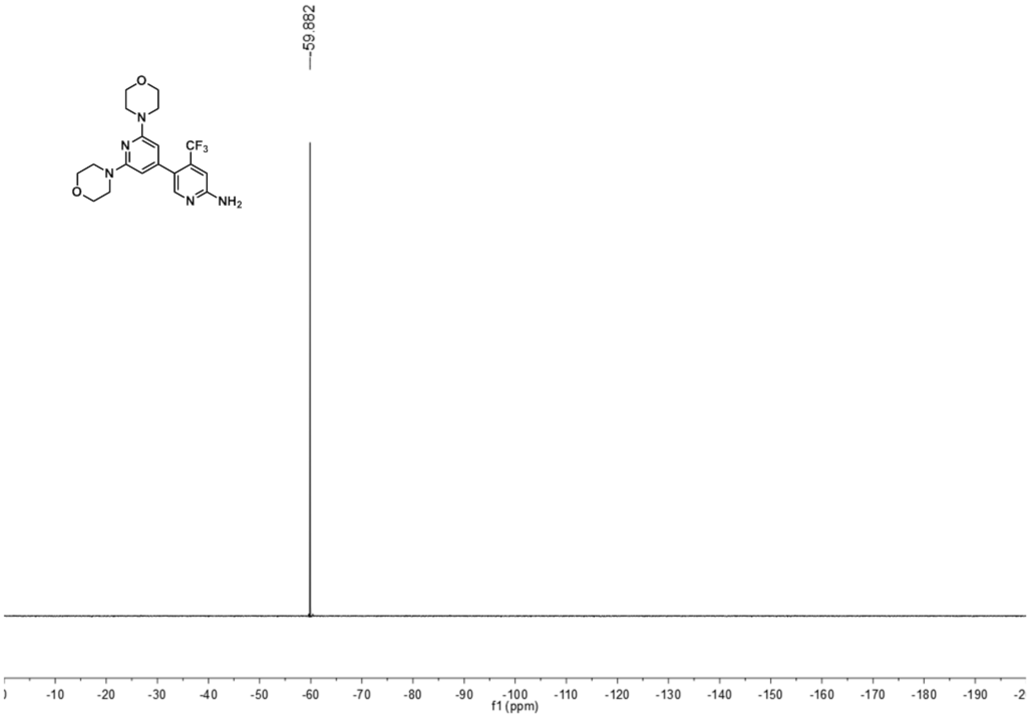

5-(2,6-dimorpholinopyrimidin-4-yl)-4-(trifluoromethyl)pyridin-2-amine (**BKM120**):

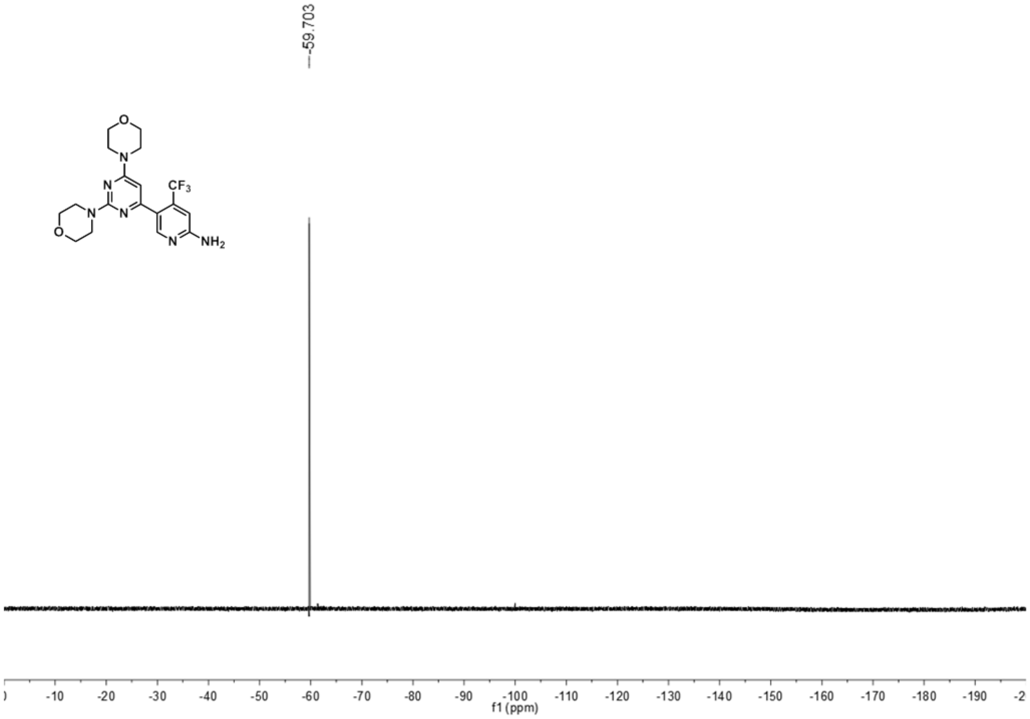

5-(4,6-Dimorpholinopyrimidin-2-yl)-4-(trifluoromethyl)pyridin-2-amine (**BKM120-R1**):

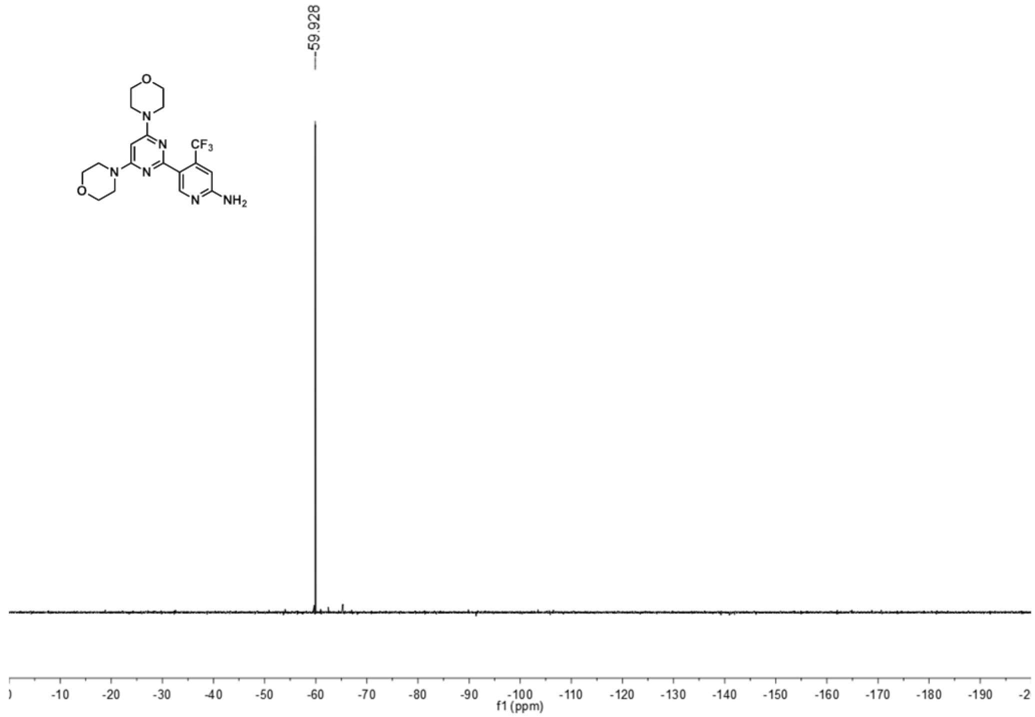

(1-(6-(6-Amino-4-(trifluoromethyl)pyridin-3-yl)-2-morpholinopyrimidin-4-yl)-3-(chloromethyl)azetidin-3-yl)methanol (**PIKiN2-R1**):

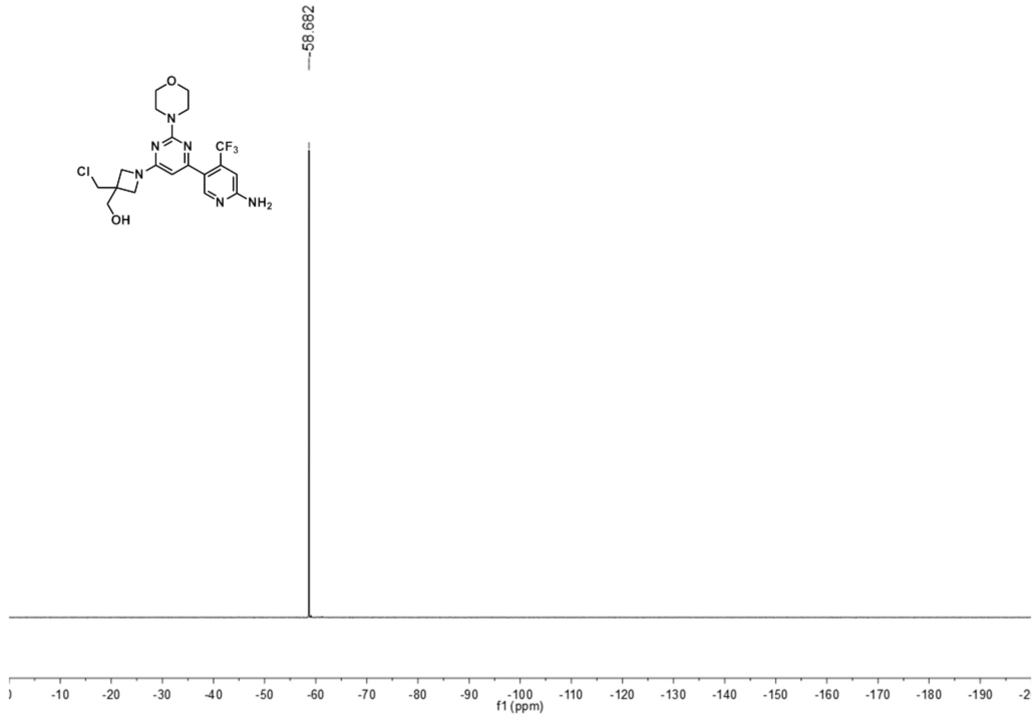

(1-(4-(6-Amino-4-(trifluoromethyl)pyridin-3-yl)-6-morpholinopyrimidin-2-yl)-3-(chloromethyl)azetidin-3-yl)methanol (**PIKiN2**):

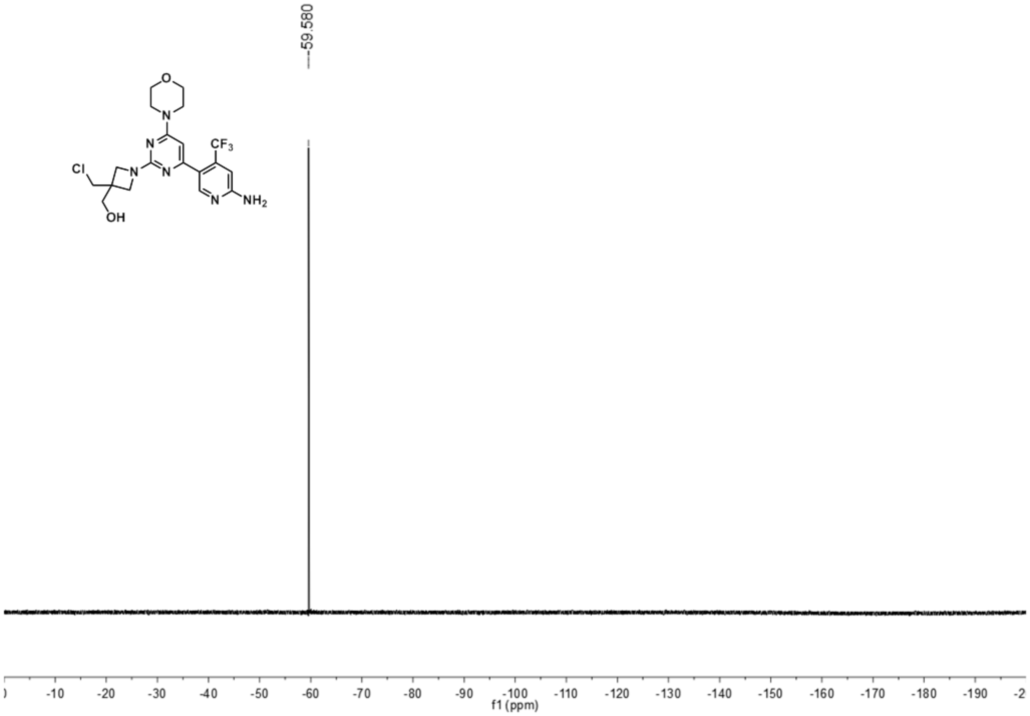

5-(2-Morpholino-6-(piperidin-1-yl)pyrimidin-4-yl)-4-(trifluoromethyl)pyridin-2-amine (**PIKiN1-R1**):

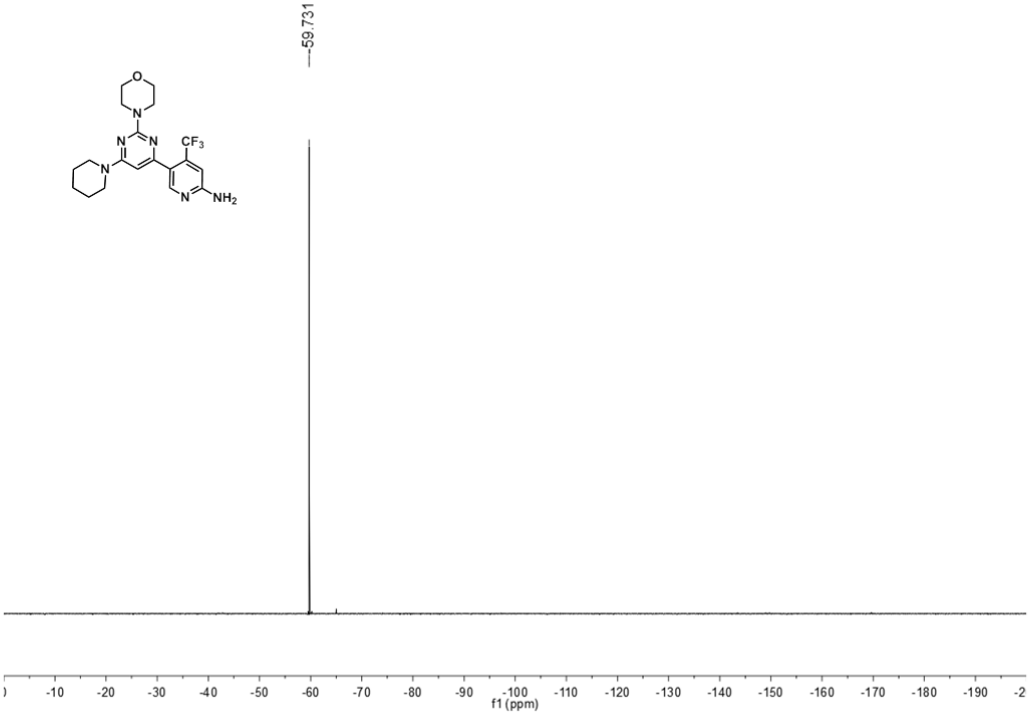

5-(6-Morpholino-2-(piperidin-1-yl)pyrimidin-4-yl)-4-(trifluoromethyl)pyridin-2-amine (**PIKiN1**):

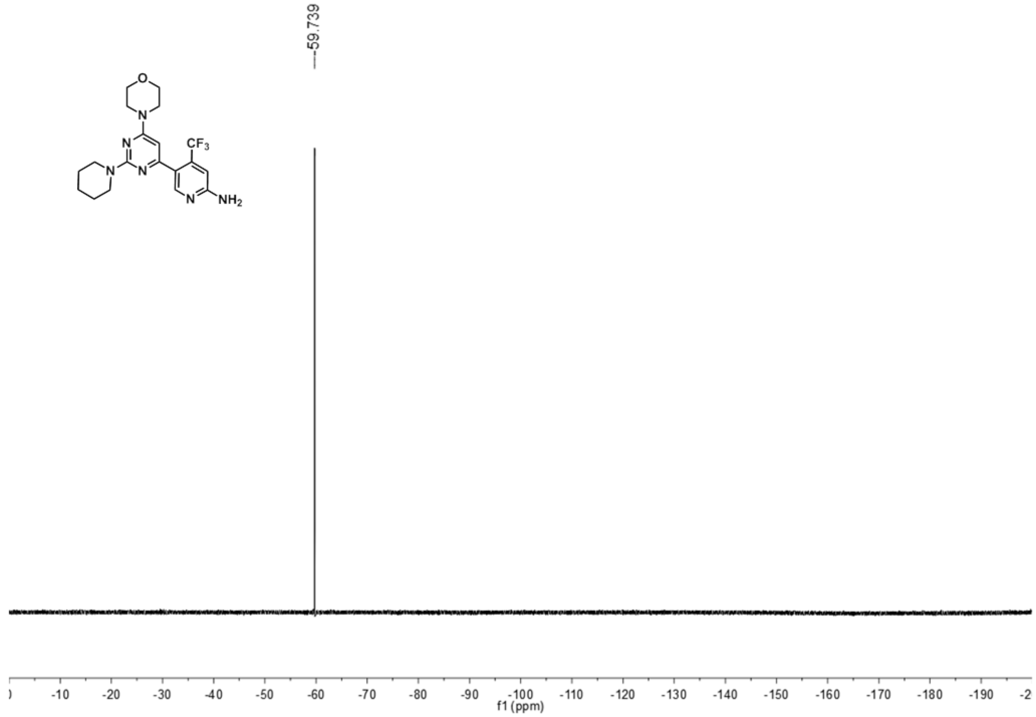

5-(2-Morpholino-6-(pyrrolidin-1-yl)pyrimidin-4-yl)-4-(trifluoromethyl)pyridin-2-amine (**MTD265**):

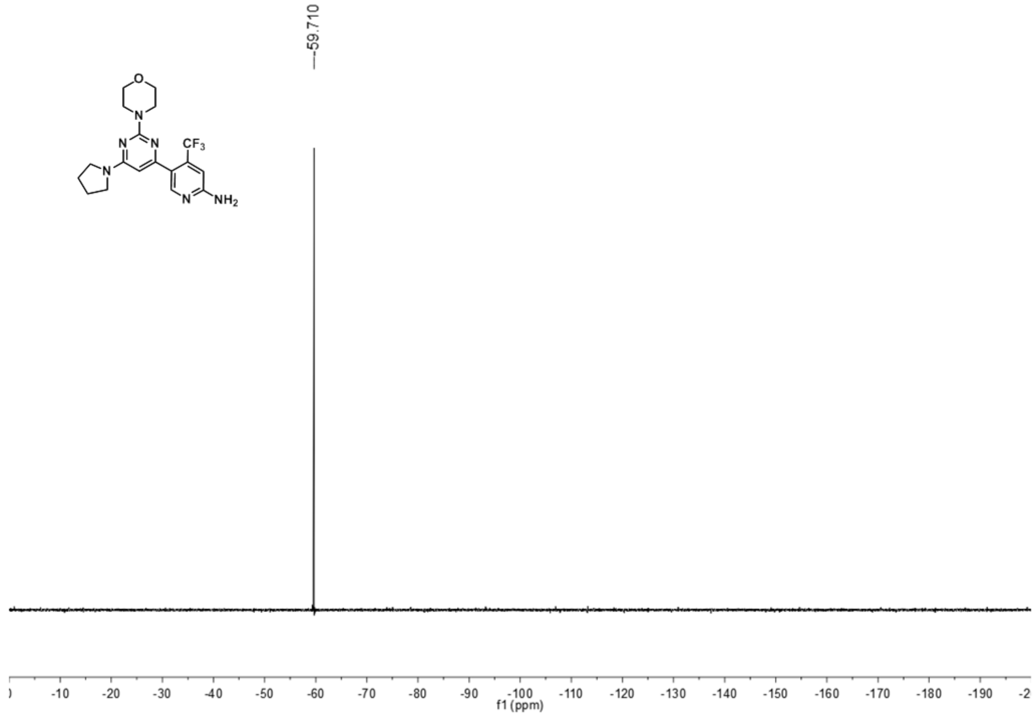

5-(6-Morpholino-2-(pyrrolidin-1-yl)pyrimidin-4-yl)-4-(trifluoromethyl)pyridin-2-amine (**MTD265-R1**):

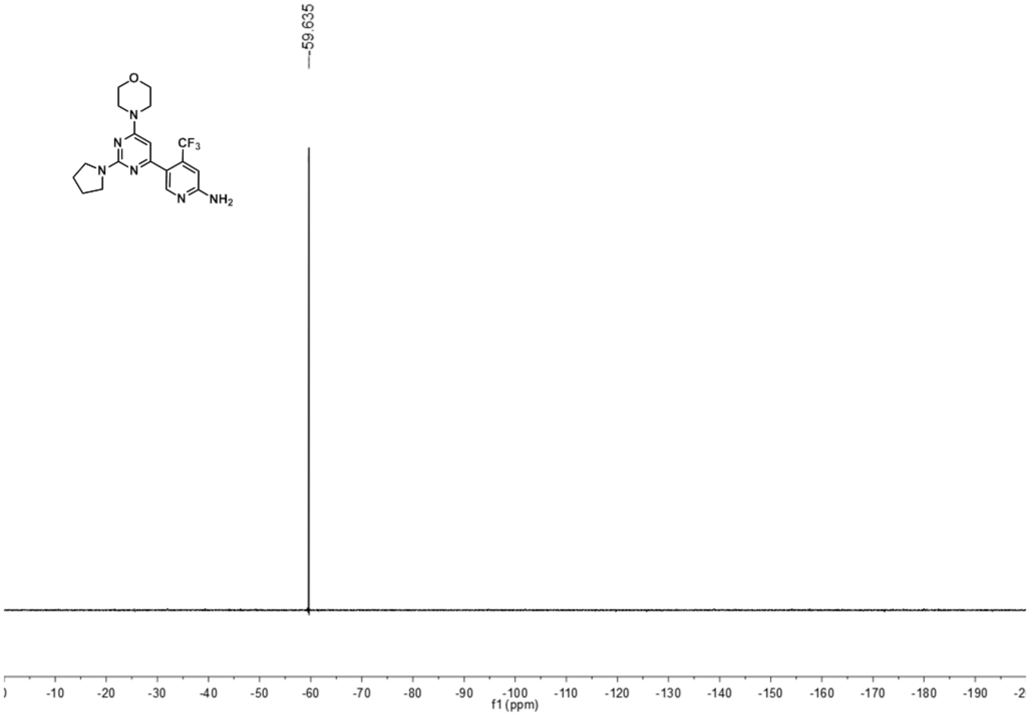

5-(4,6-Dimorpholino-1,3,5-triazin-2-yl)-4-(trifluoromethyl)pyridin-2-amine (**PQR309**):

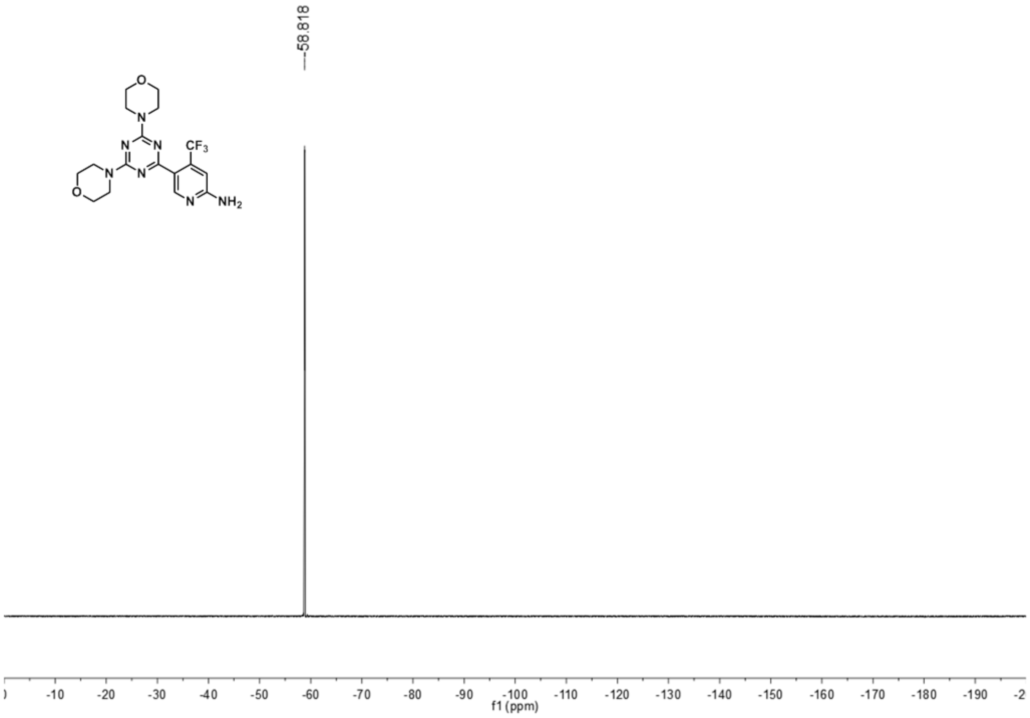

(1-(4-(6-Amino-4-(trifluoromethyl)pyridin-3-yl)-6-morpholino-1,3,5-triazin-2-yl)-3-(chloromethyl)azetidin-3-yl)methanol (**PIKiN3**):

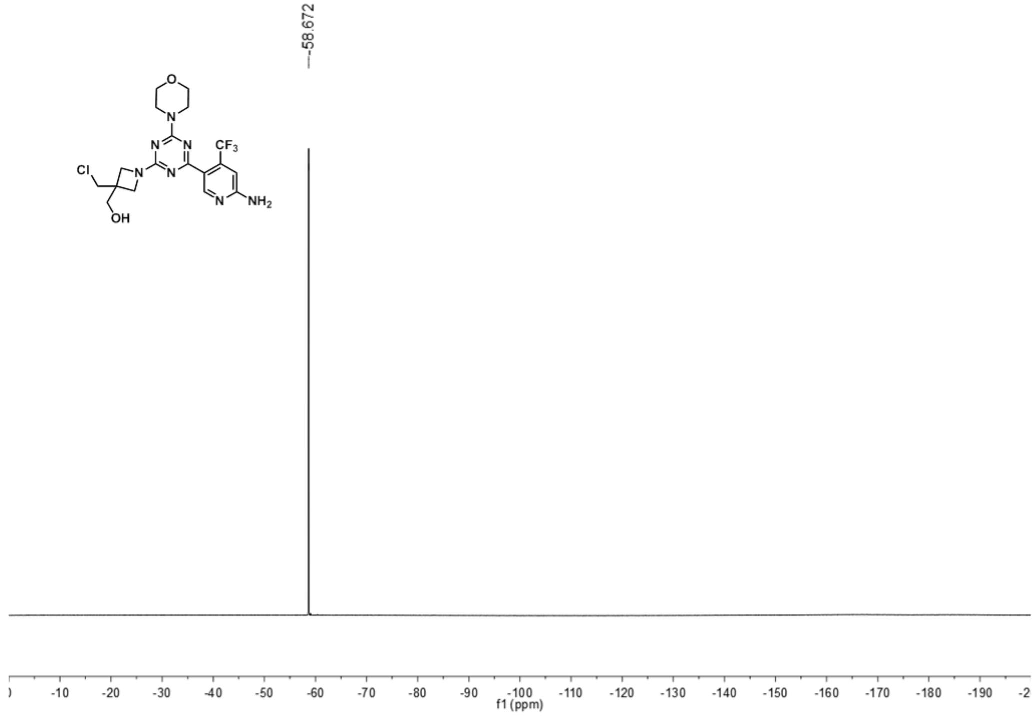

$^{13}C\{^{1}H\}$ NMR Spectra

N′-(5-Bromo-4-(trifluoromethyl)pyridin-2-yl)-*N*,*N*-dimethylformimidamide (**2**):

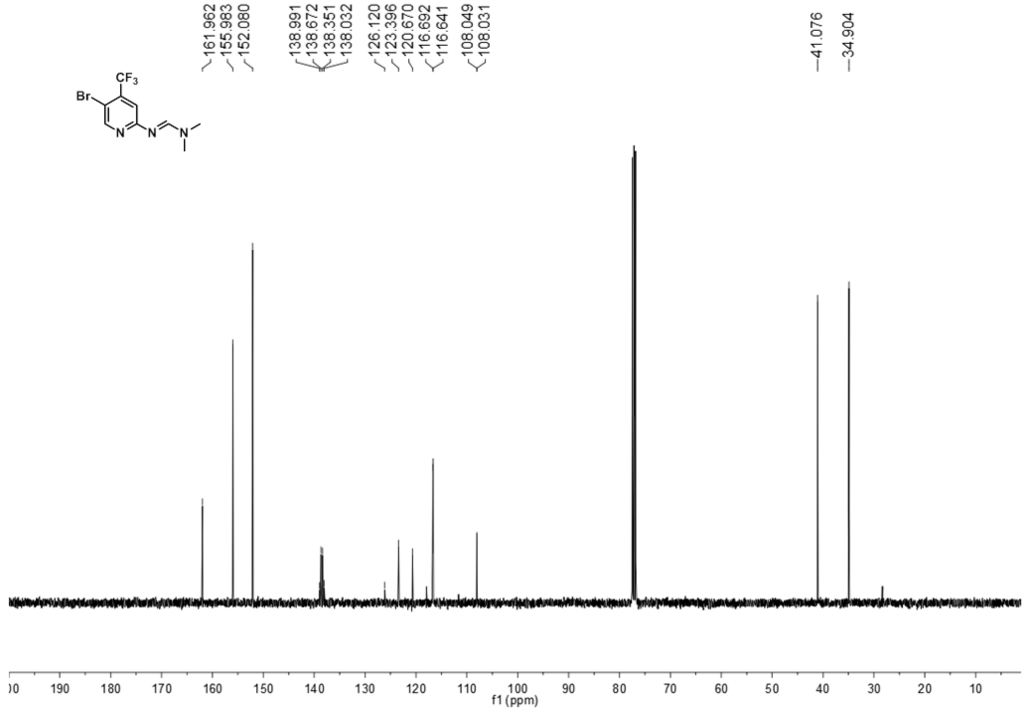

N,*N*-Dimethyl-*N′*-(5-(4,4,5,5-tetramethyl-1,3,2-dioxaborolan-2-yl)-4-(trifluoromethyl)pyridin-2-yl)formimidamide (**3**):

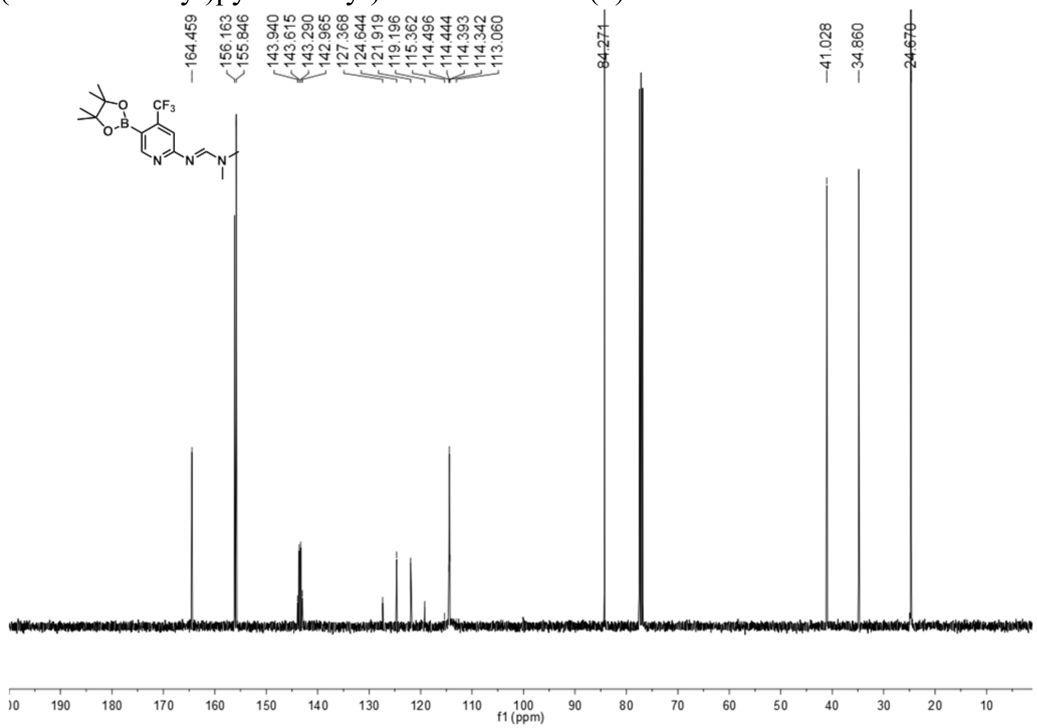

tert-Butyl (5-bromo-4-(trifluoromethyl)pyridin-2-yl)carbamate (**4**):

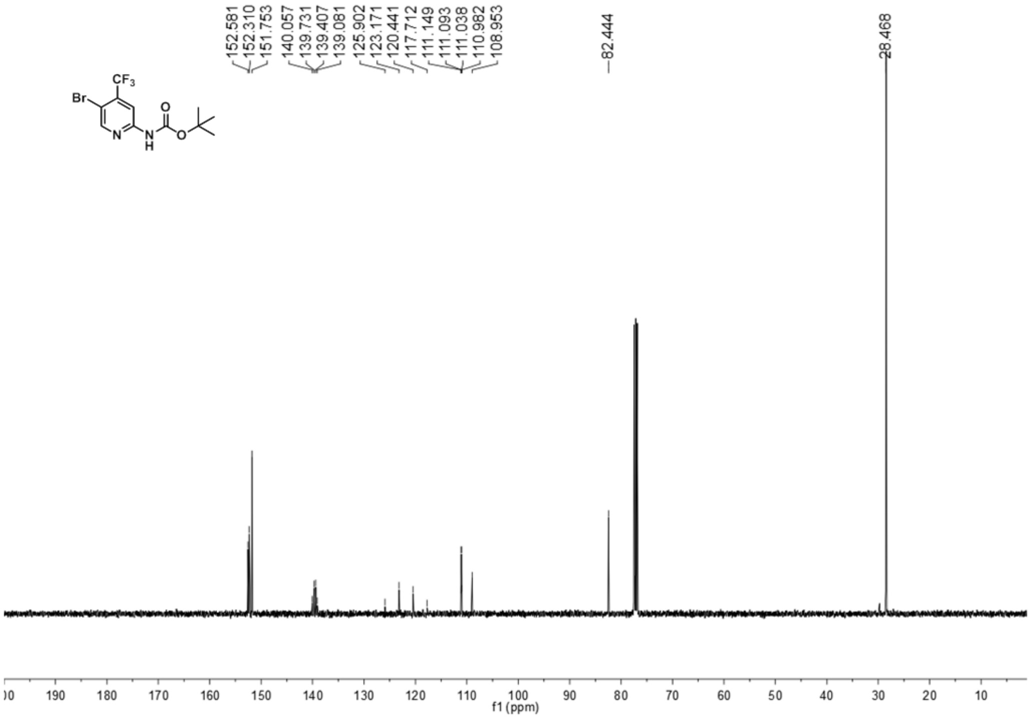

tert-Butyl (2',6'-dichloro-4-(trifluoromethyl)-(3,4'-bipyridin)-6-yl)carbamate (**6**):

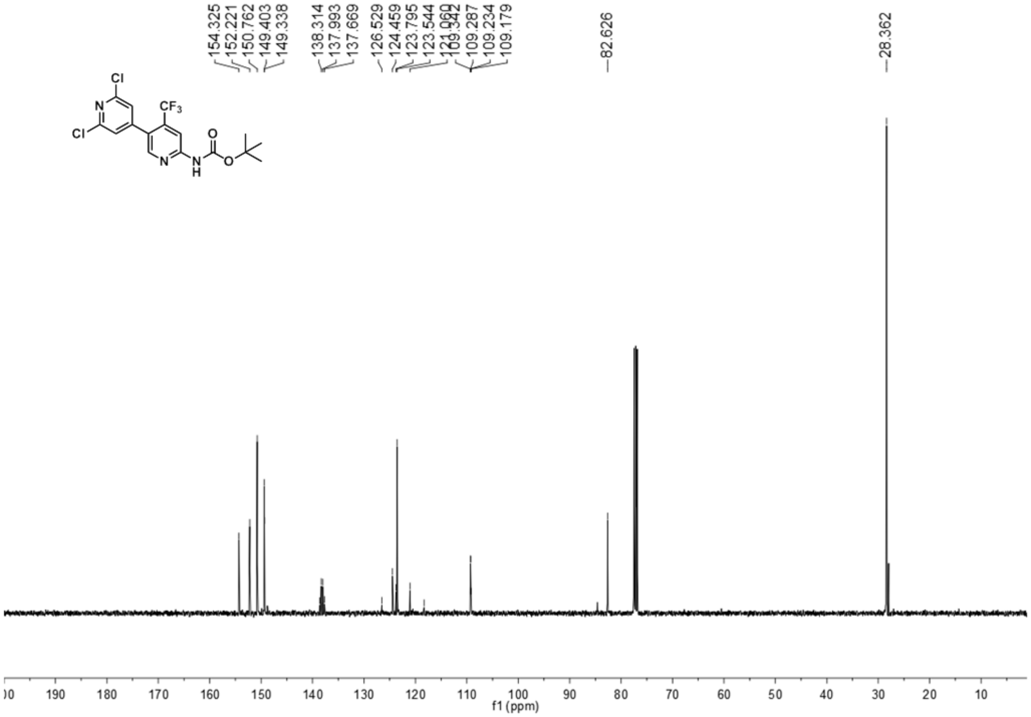

2',6'-Dimorpholino-4-(trifluoromethyl)-(3,4'-bipyridin)-6-amine (**MTD147**):

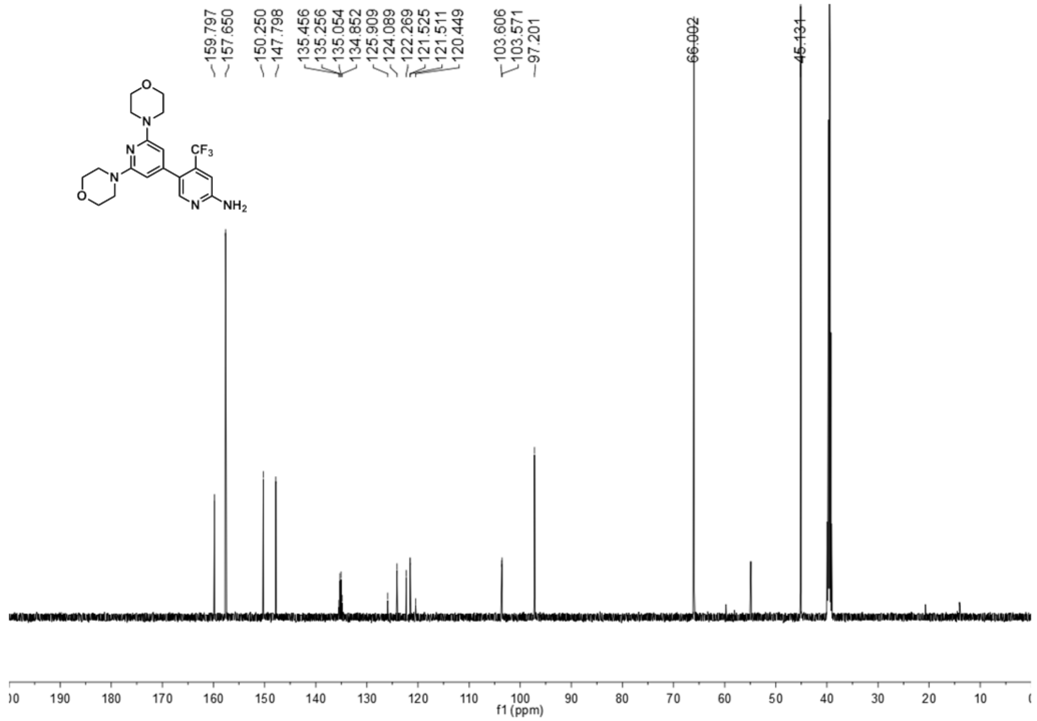

4,4'-(6-Chloropyrimidine-2,4-diyl)dimorpholine (**8**):

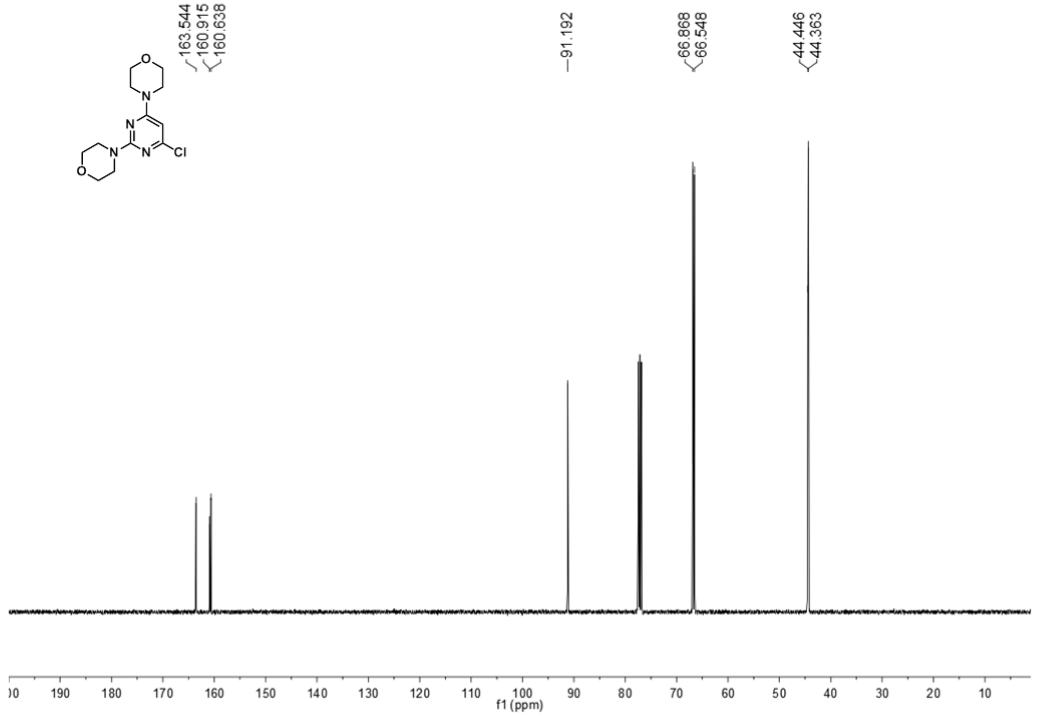

4,4'-(2-Chloropyrimidine-4,6-diyl)dimorpholine (**9**):

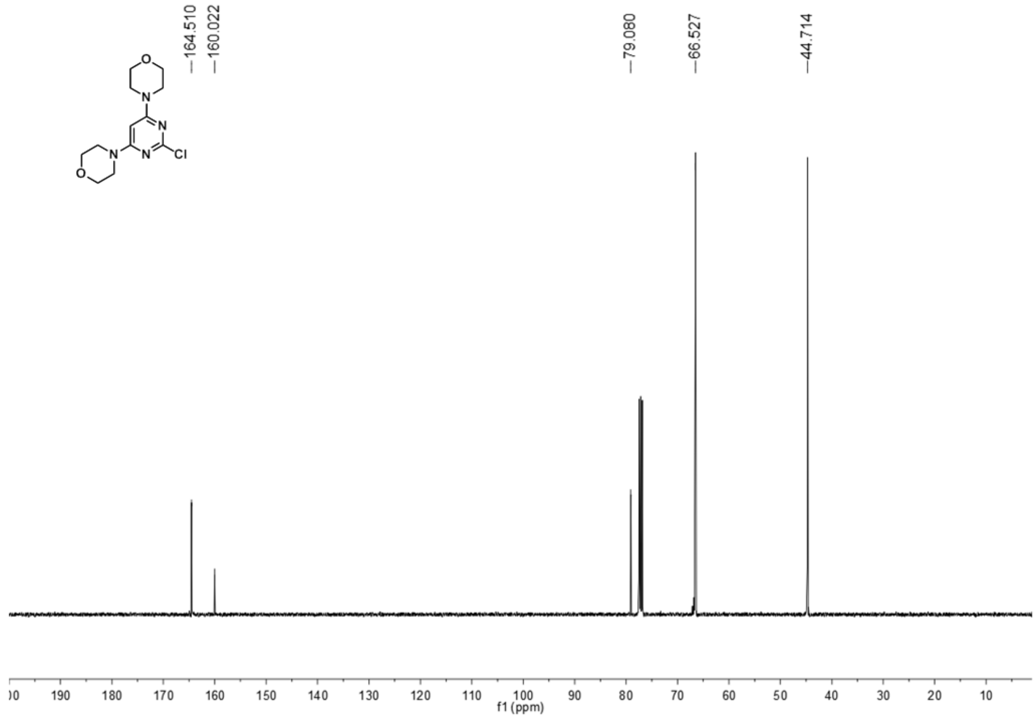

5-(2,6-Dimorpholinopyrimidin-4-yl)-4-(trifluoromethyl)pyridin-2-amine (**BKM120**):

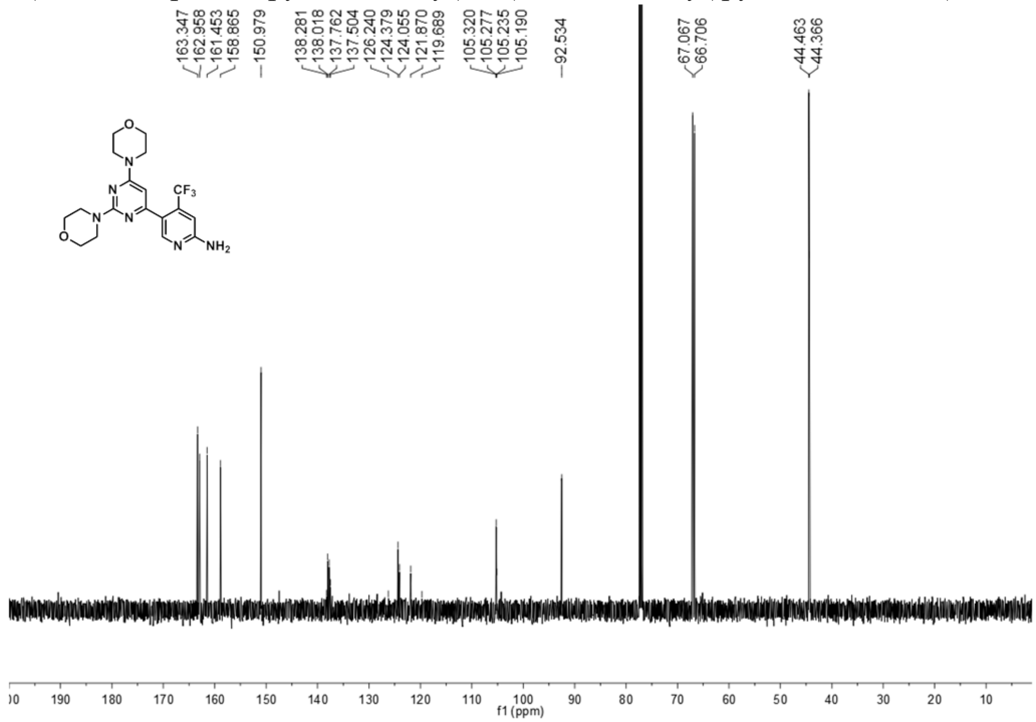

5-(4,6-Dimorpholinopyrimidin-2-yl)-4-(trifluoromethyl)pyridin-2-amine (**BKM120-R1**):

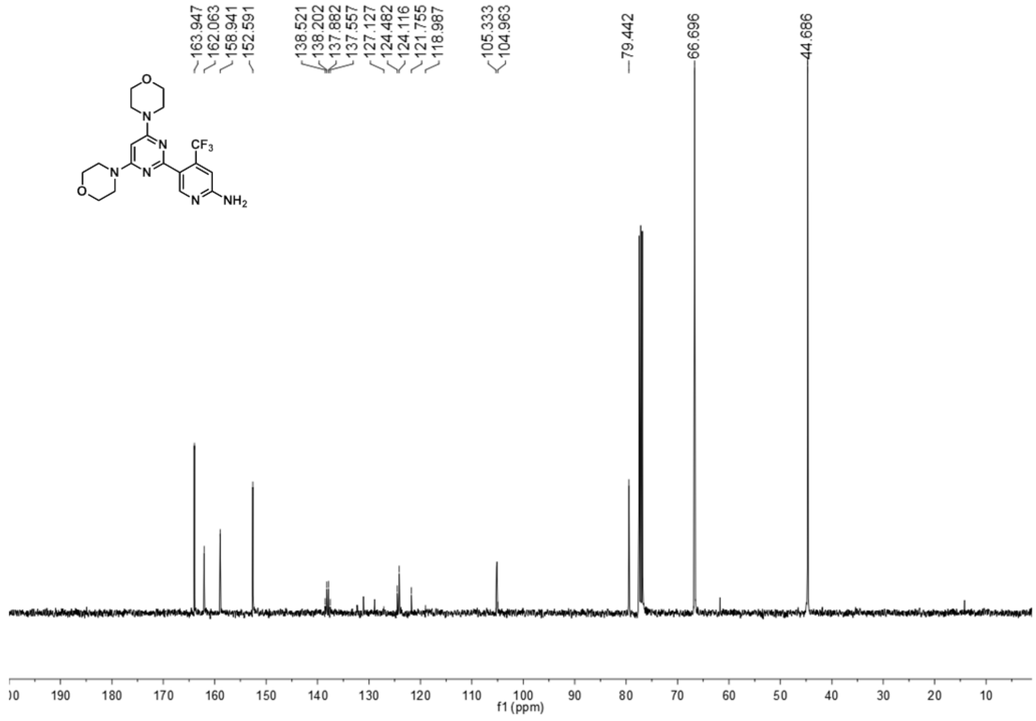

4-(4,6-Dichloropyrimidin-2-yl)morpholine (**10**):

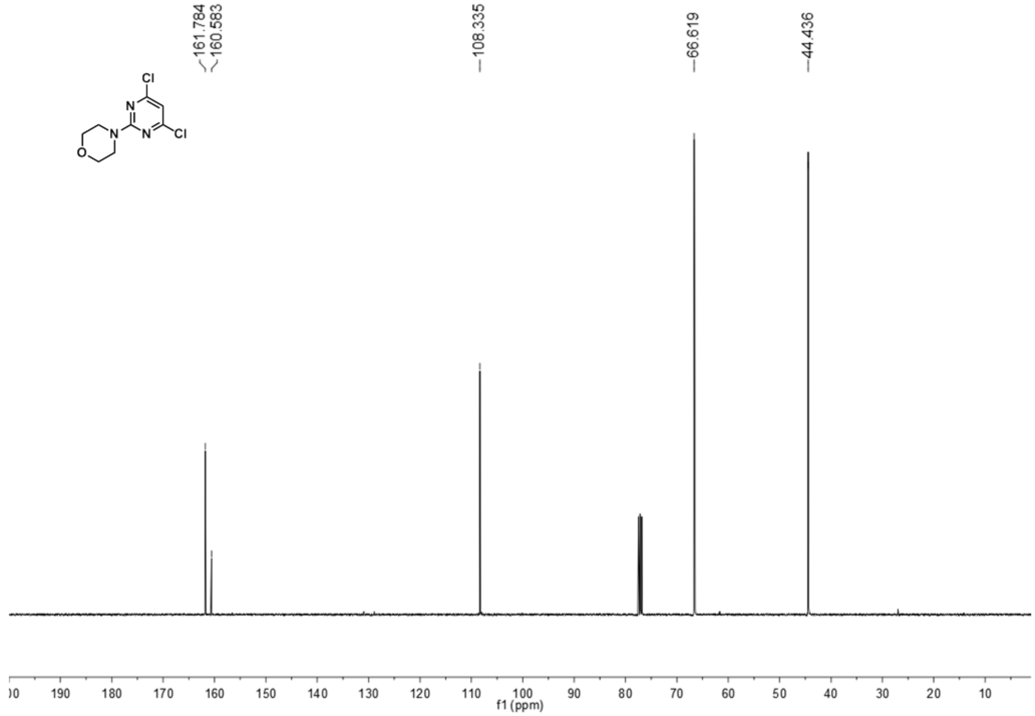

4-(2,6-Dichloropyrimidin-4-yl)morpholine (**11**):

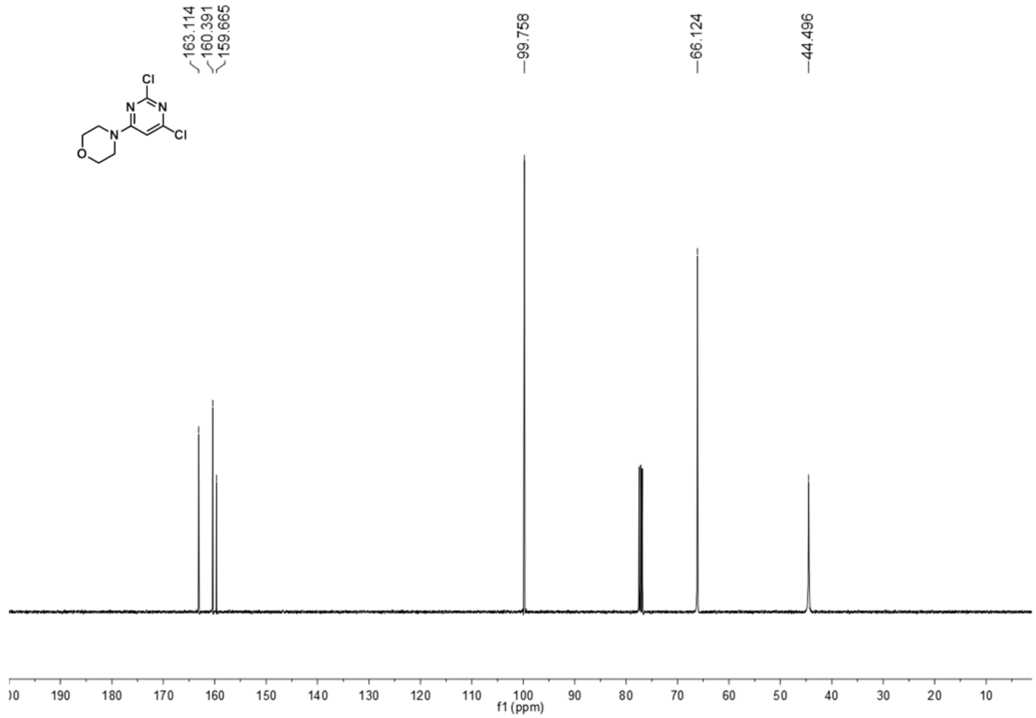

6-(6-Chloro-2-morpholinopyrimidin-4-yl)-2-oxa-6-azaspiro[3.3]heptane (**12**):

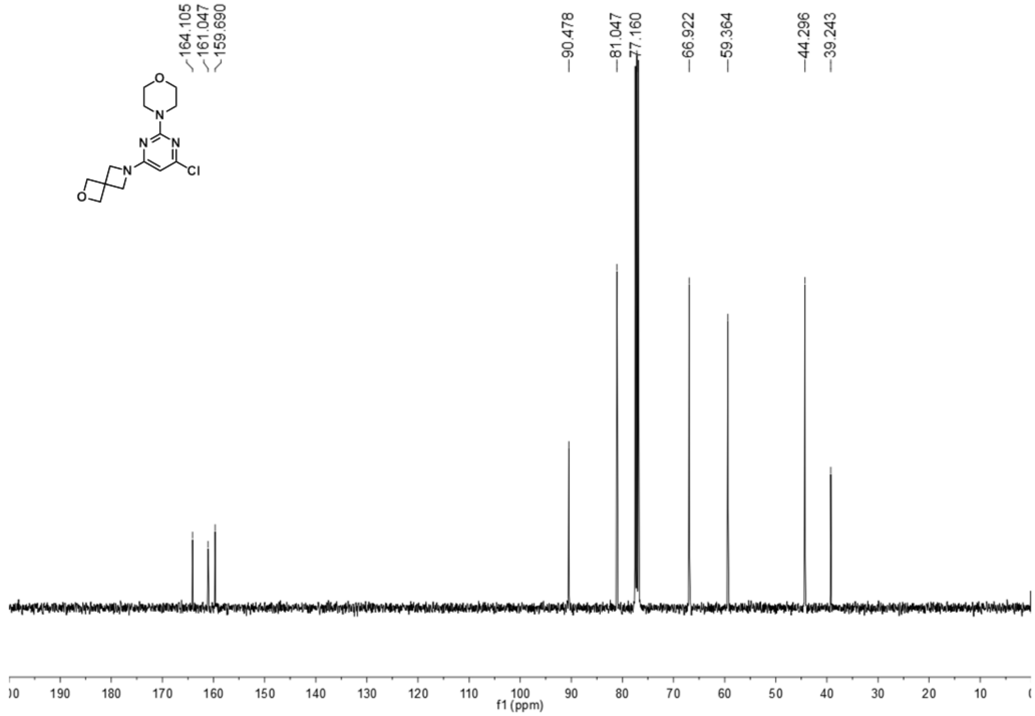

(1-(6-(6-Amino-4-(trifluoromethyl)pyridin-3-yl)-2-morpholinopyrimidin-4-yl)-3-(chloromethyl)azetidin-3-yl)methanol (**PIKiN2-R1**):

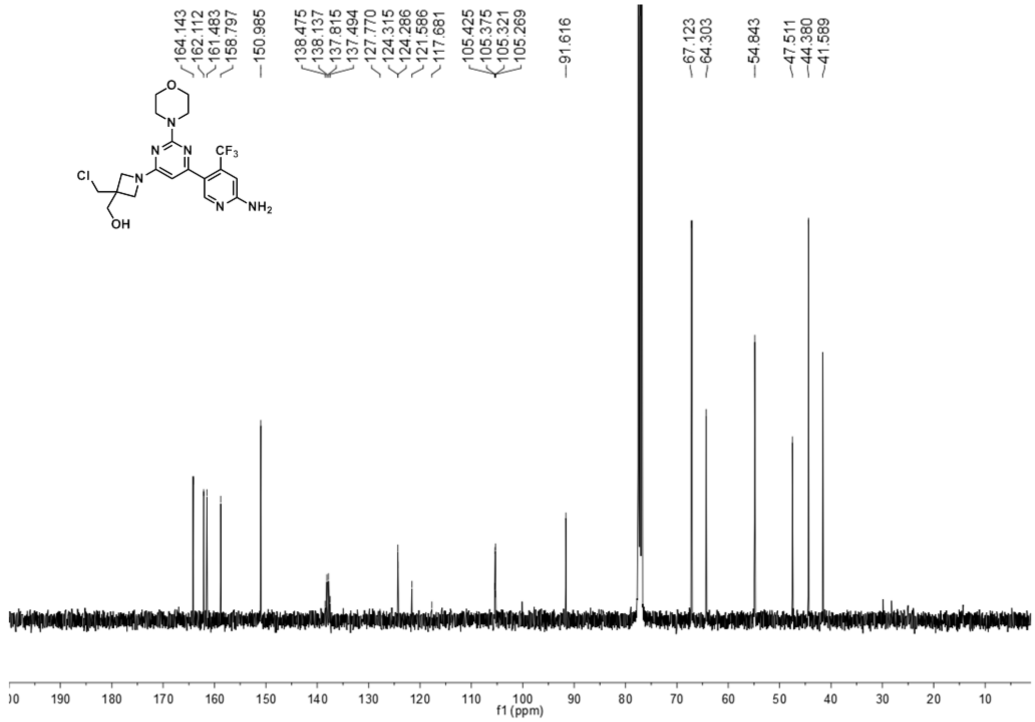

6-(4-Chloro-6-morpholinopyrimidin-2-yl)-2-oxa-6-azaspiro[3.3]heptane (**13**):

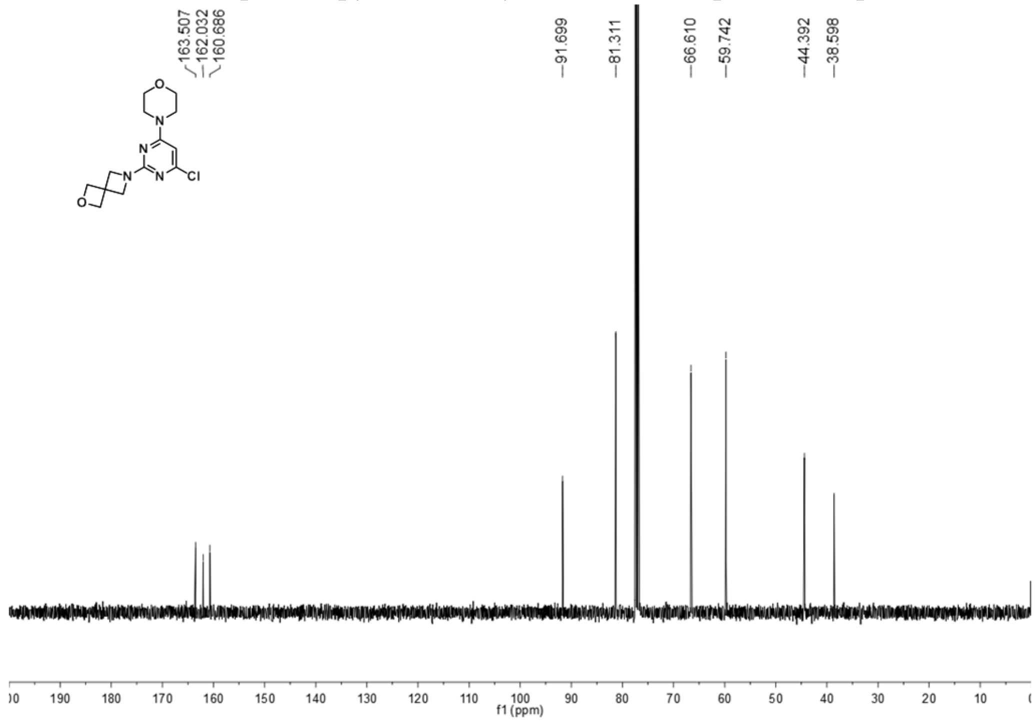

(1-(4-(6-Amino-4-(trifluoromethyl)pyridin-3-yl)-6-morpholinopyrimidin-2-yl)-3-(chloromethyl)azetidin-3-yl)methanol (**PIKiN2**):

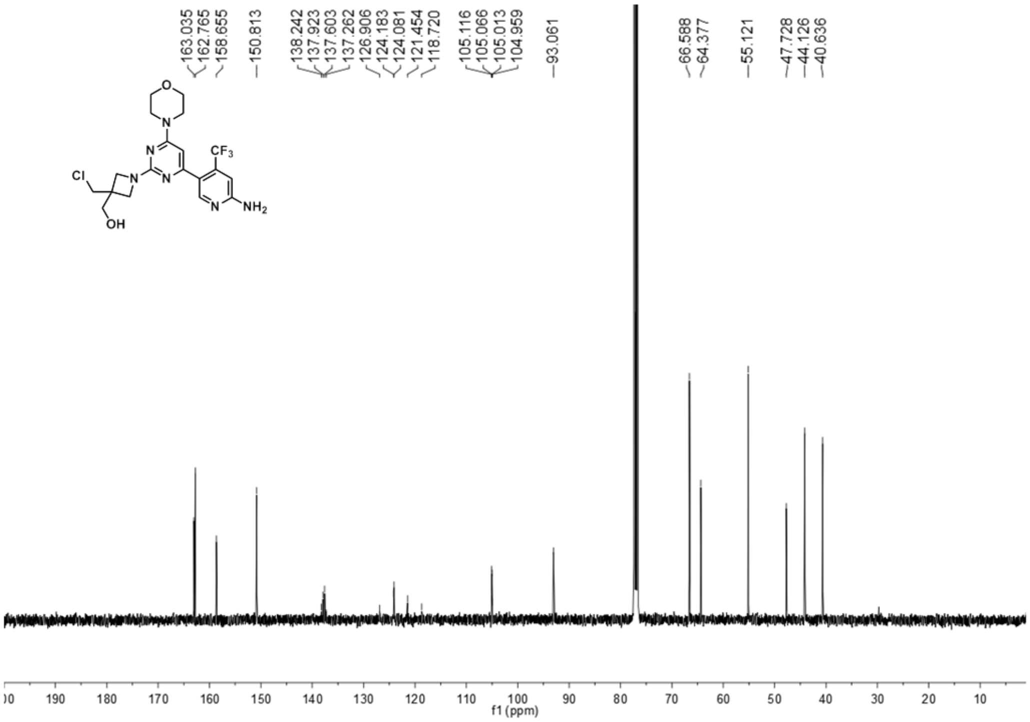

4-(4-Chloro-6-(piperidin-1-yl)pyrimidin-2-yl)morpholine (**14**):

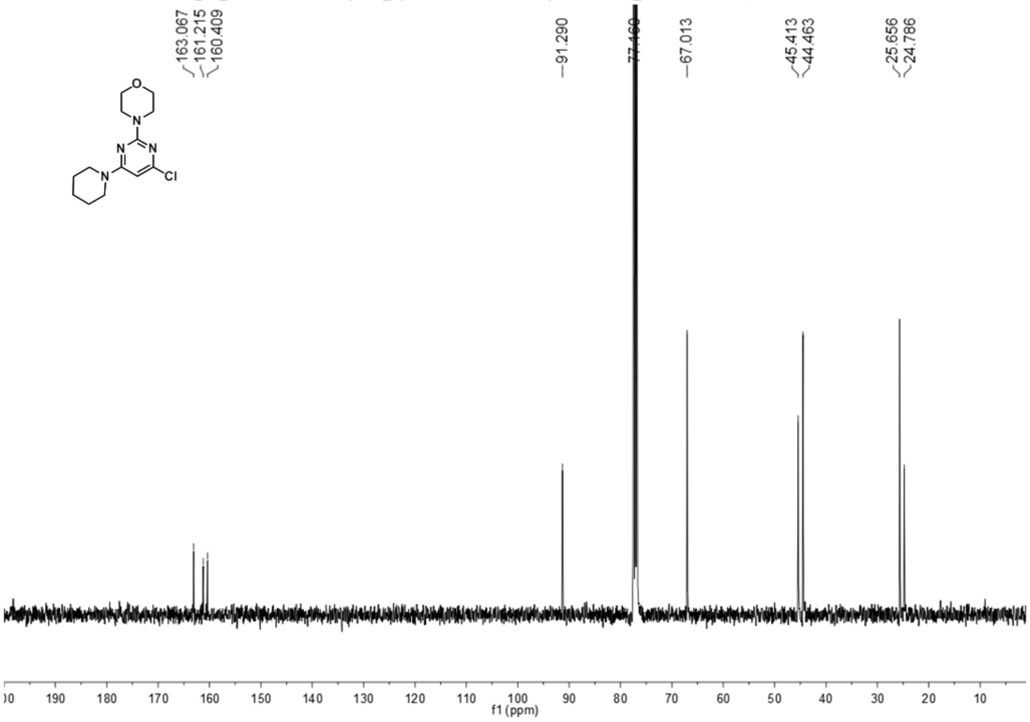

5-(2-Morpholino-6-(piperidin-1-yl)pyrimidin-4-yl)-4-(trifluoromethyl)pyridin-2-amine (**PIKiN1-R1**):

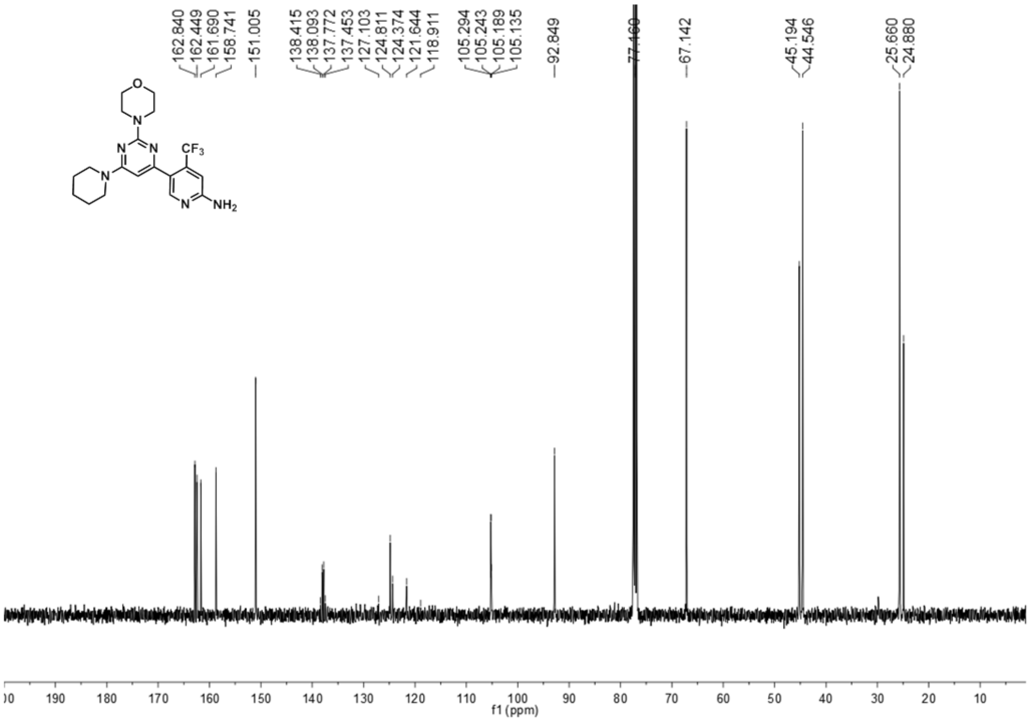

4-(6-Chloro-2-(piperidin-1-yl)pyrimidin-4-yl)morpholine (**15**):

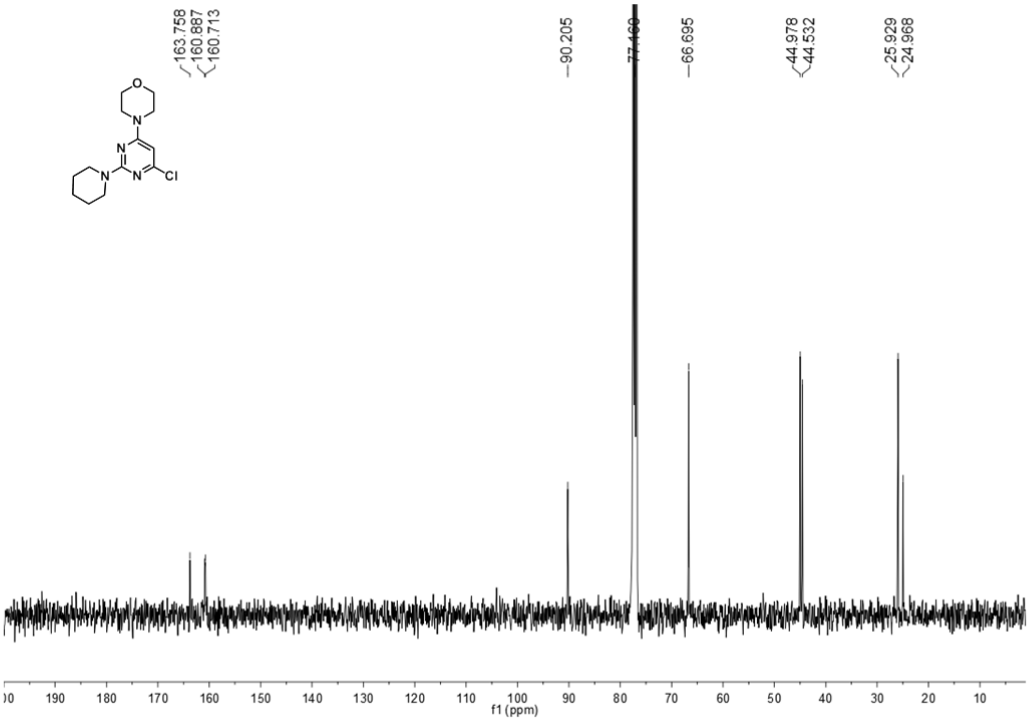

5-(6-Morpholino-2-(piperidin-1-yl)pyrimidin-4-yl)-4-(trifluoromethyl)pyridin-2-amine (**PIKiN1**):

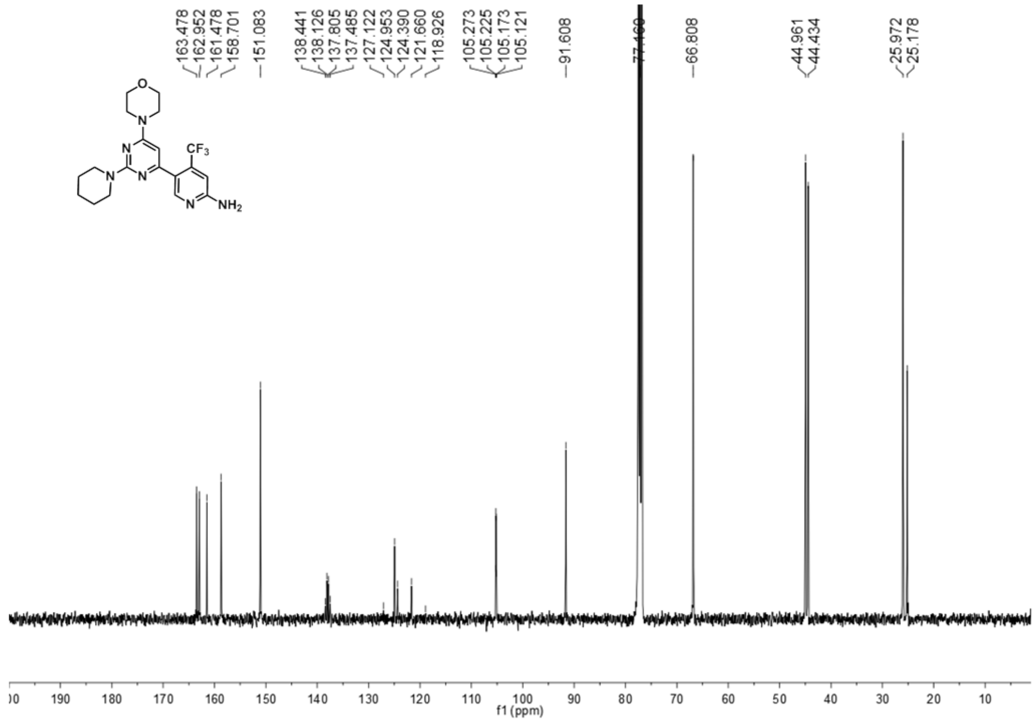

4-(4-Chloro-6-(pyrrolidin-1-yl)pyrimidin-2-yl)morpholine (**16**):

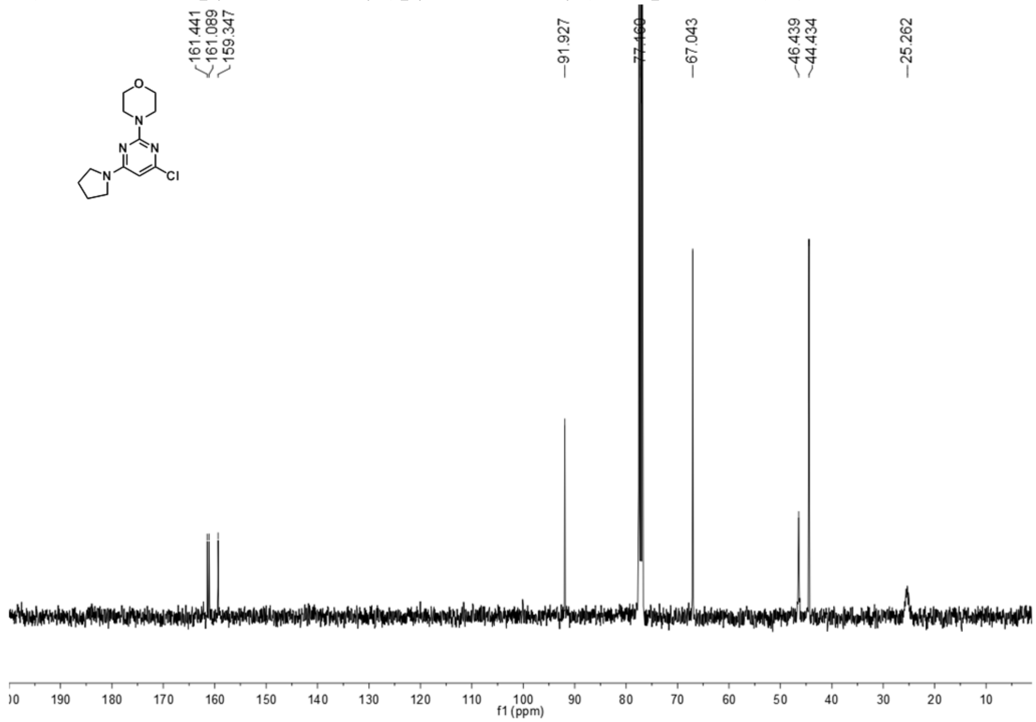

5-(2-Morpholino-6-(pyrrolidin-1-yl)pyrimidin-4-yl)-4-(trifluoromethyl)pyridin-2-amine (**MTD265**):

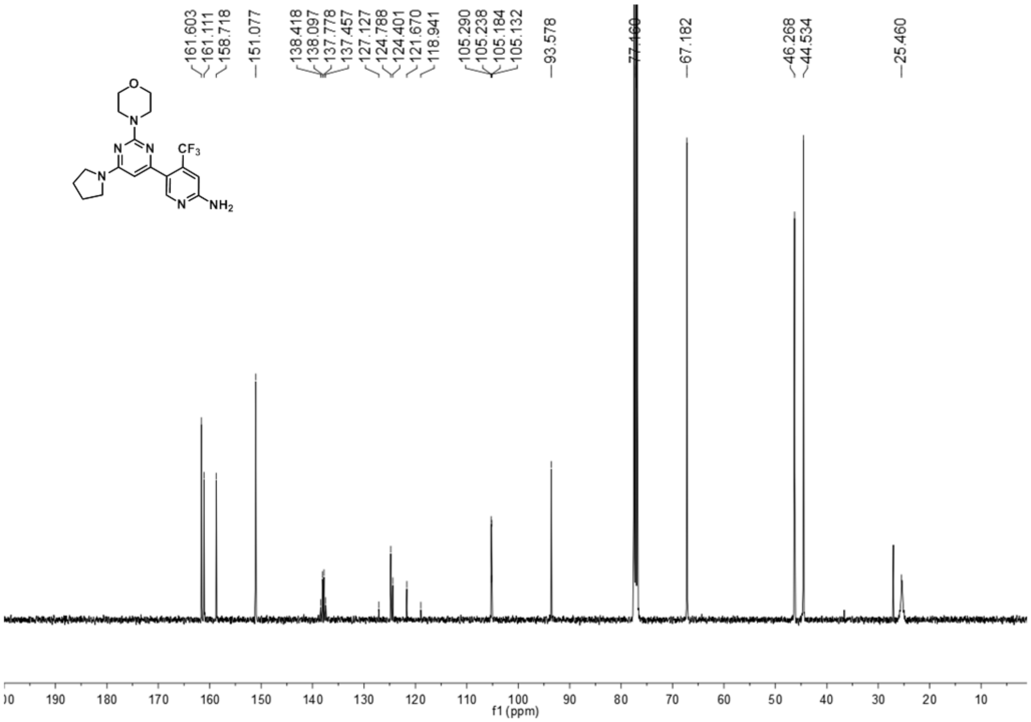

4-(6-Chloro-2-(pyrrolidin-1-yl)pyrimidin-4-yl)morpholine (**17**):

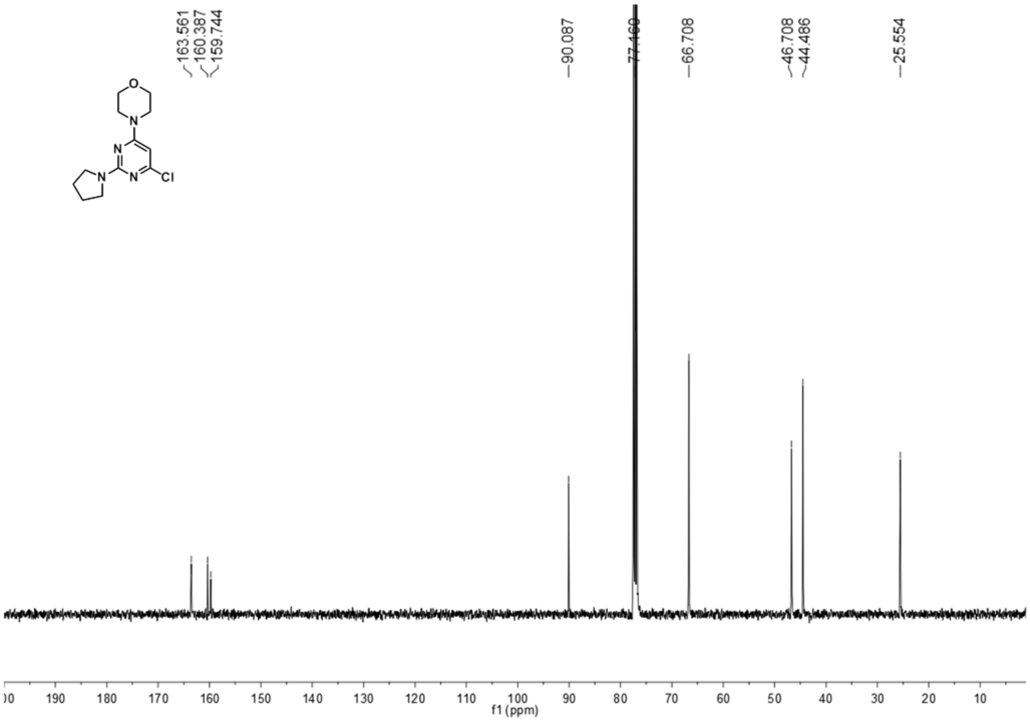

5-(6-Morpholino-2-(pyrrolidin-1-yl)pyrimidin-4-yl)-4-(trifluoromethyl)pyridin-2-amine (**MTD265-R1**):

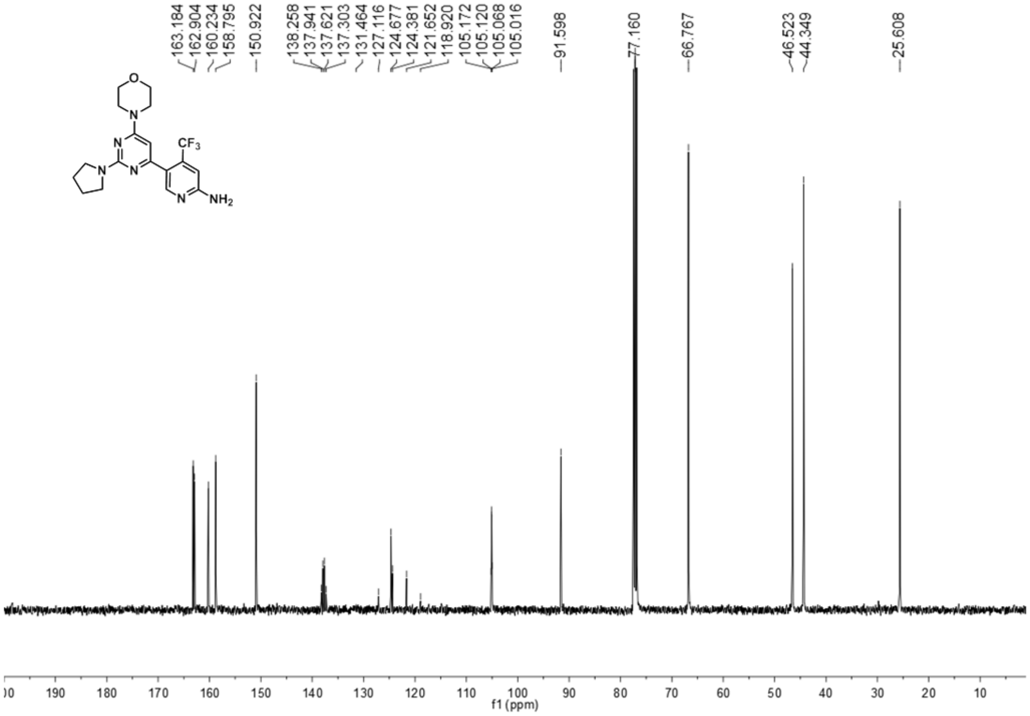

4,4'-(6-Chloro-1,3,5-triazine-2,4-diyl)dimorpholine (**19**):

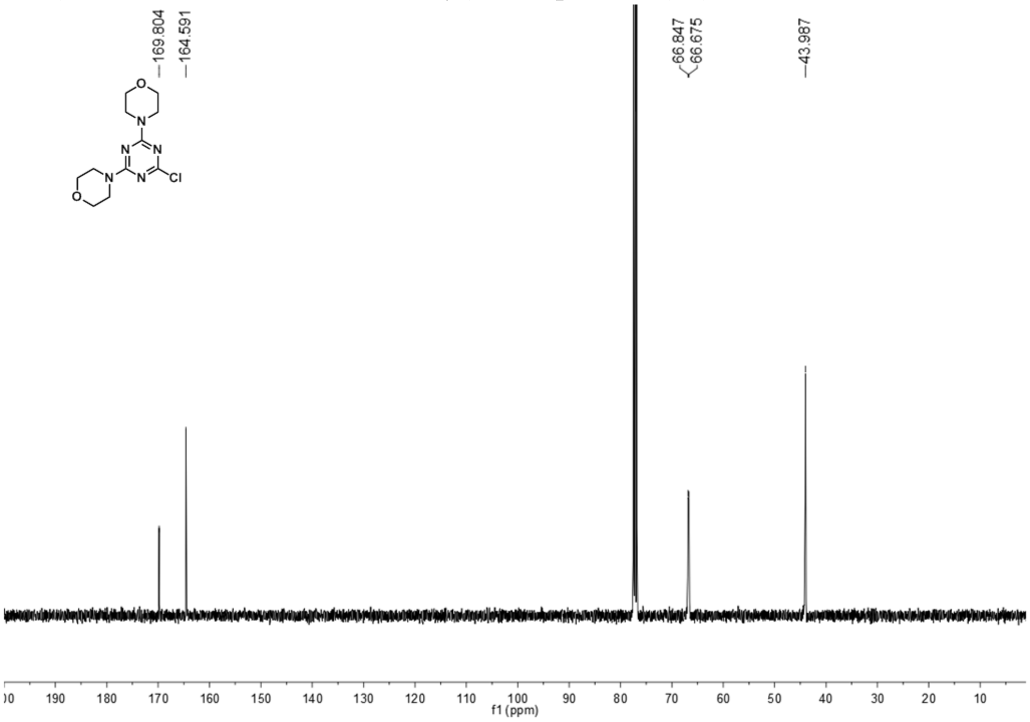

5-(4,6-Dimorpholino-1,3,5-triazin-2-yl)-4-(trifluoromethyl)pyridin-2-amine (**PQR309**):

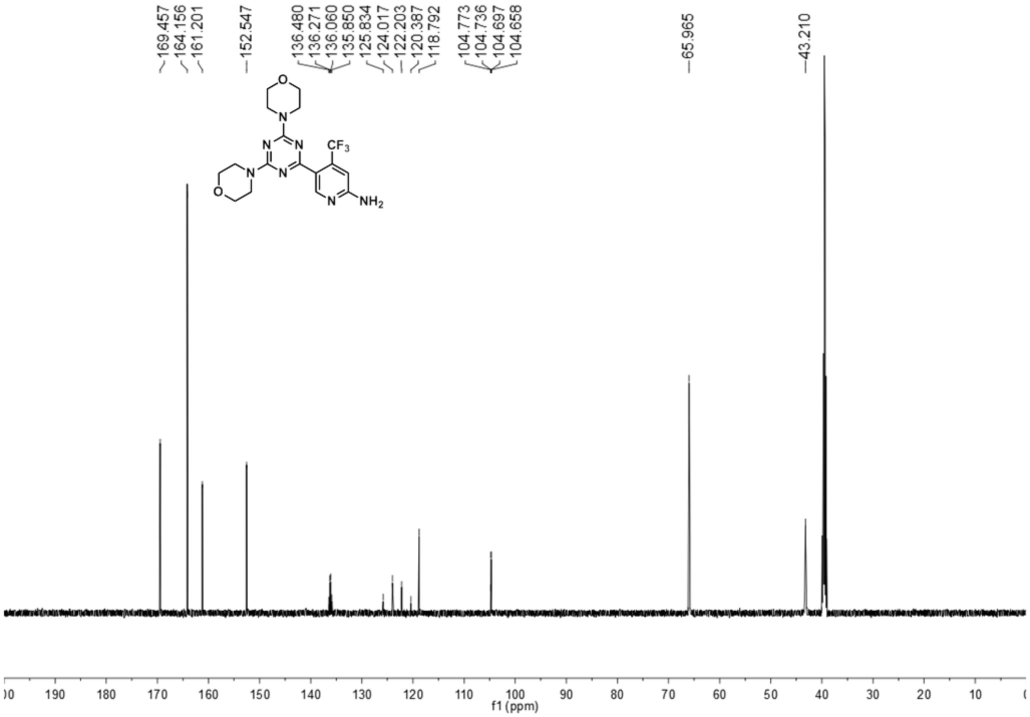

4-(4,6-Dichloro-1,3,5-triazin-2-yl)morpholine (**20**):

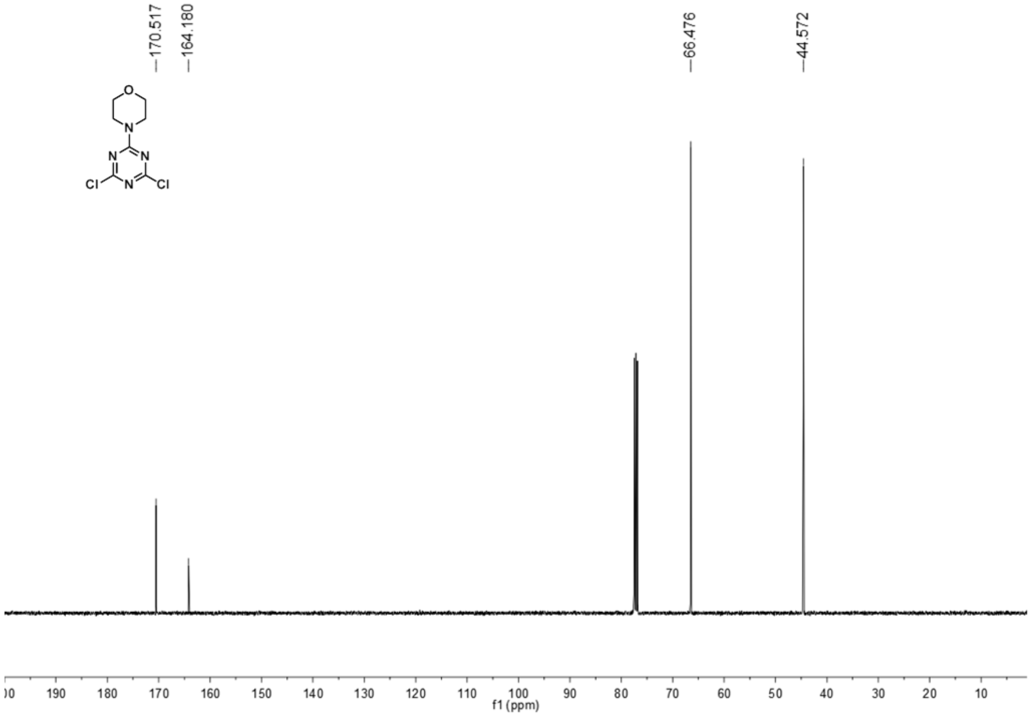

6-(4-Chloro-6-morpholino-1,3,5-triazin-2-yl)-2-oxa-6-azaspiro[3.3]heptane (**21**):

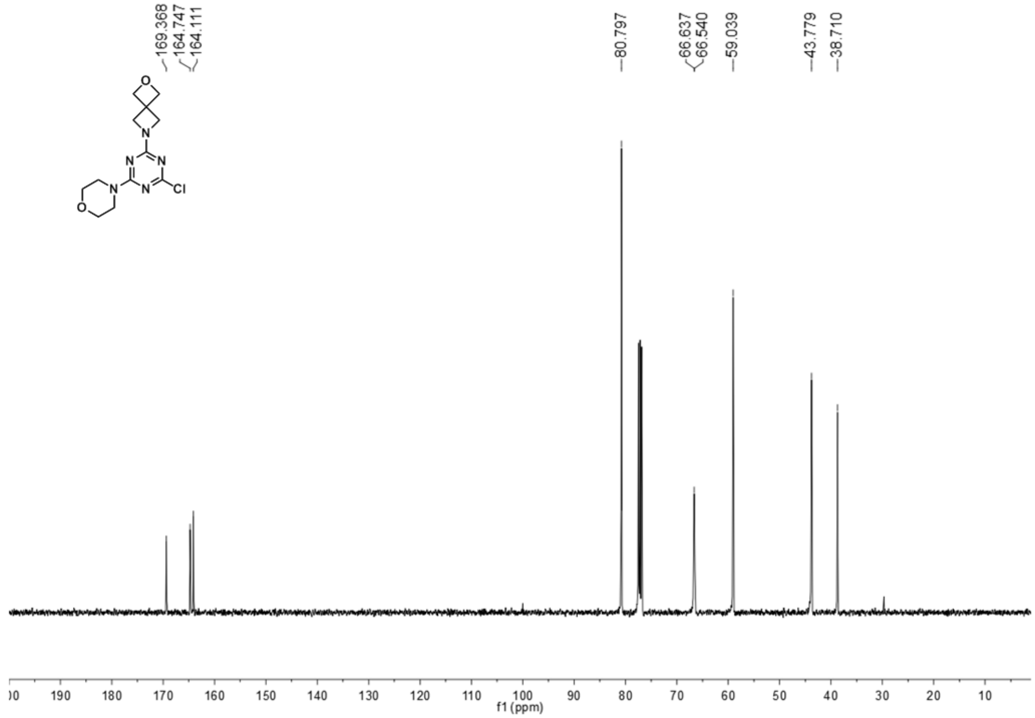

(1-(4-(6-Amino-4-(trifluoromethyl)pyridin-3-yl)-6-morpholino-1,3,5-triazin-2-yl)-3-(chloromethyl)azetidin-3-yl)methanol (**PIKiN3**):

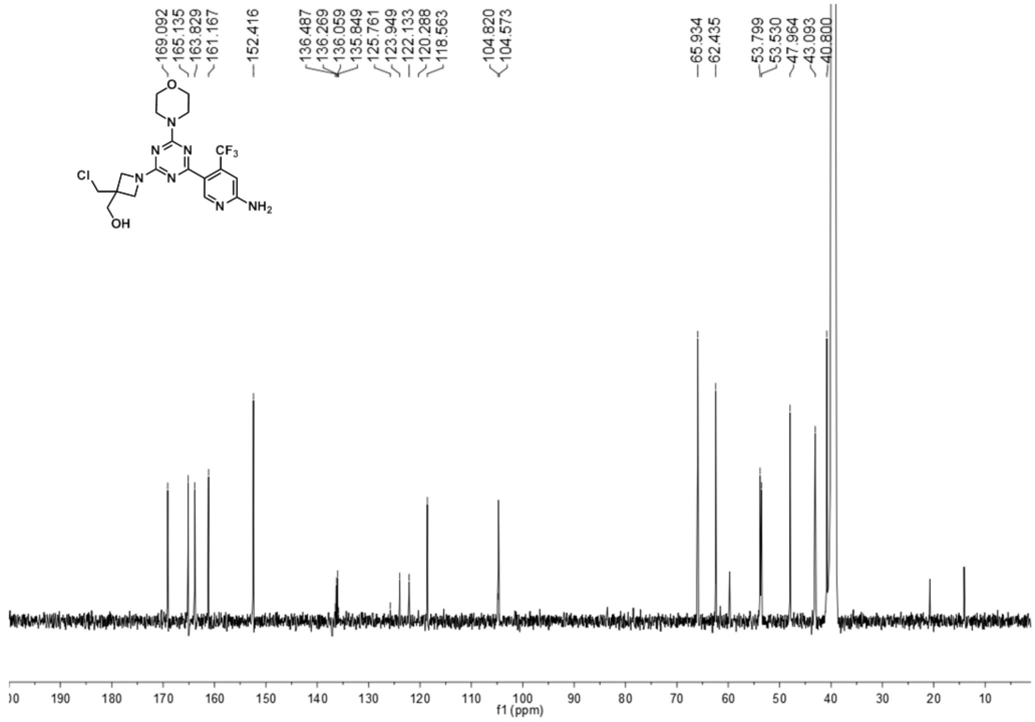

HRMS Spectra:

N′-(5-Bromo-4-(trifluoromethyl)pyridin-2-yl)-*N*,*N*-dimethylformimidamide (**2**):

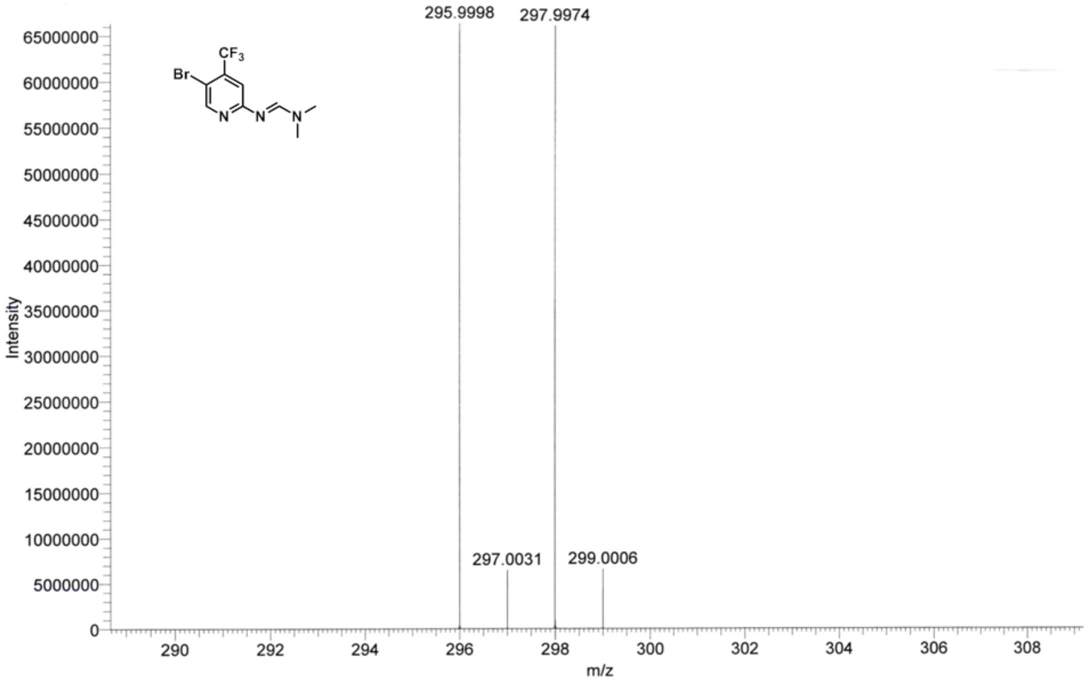

N,*N*-Dimethyl-*N′*-(5-(4,4,5,5-tetramethyl-1,3,2-dioxaborolan-2-yl)-4-(trifluoromethyl)pyridin-2-yl)formimidamide (**3**):

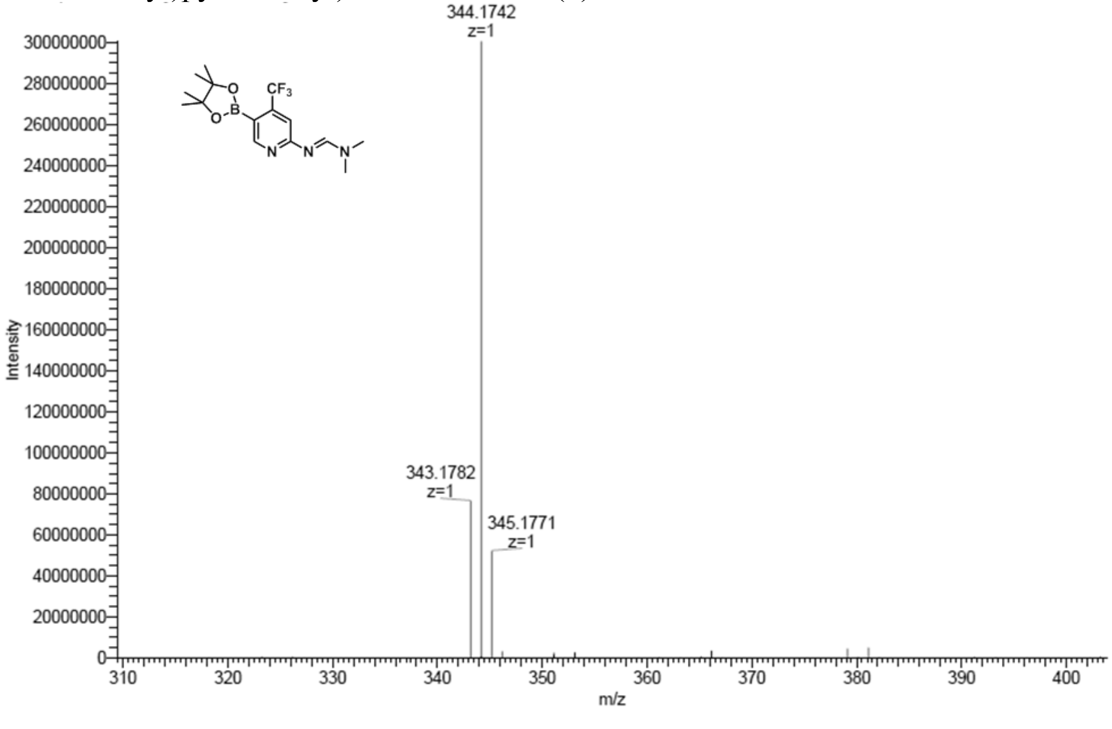

tert-Butyl (5-bromo-4-(trifluoromethyl)pyridin-2-yl)carbamate (**4**):

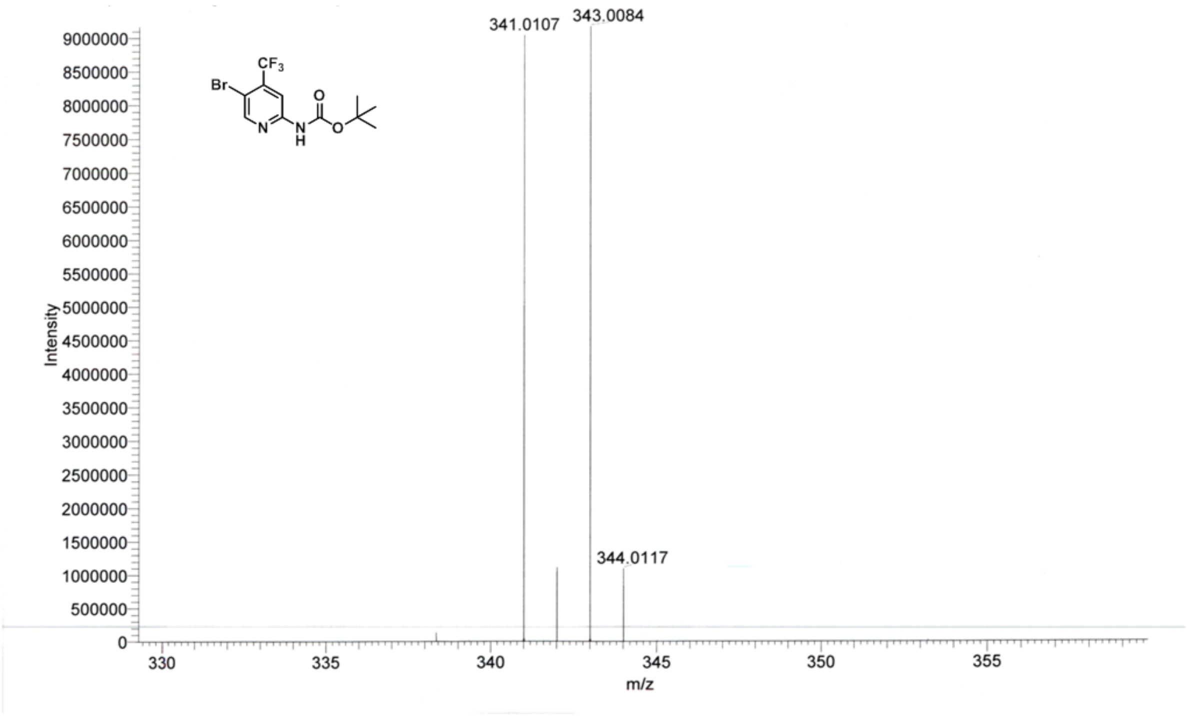

tert-Butyl (2',6'-dichloro-4-(trifluoromethyl)-(3,4'-bipyridin)-6-yl)carbamate (**6**):

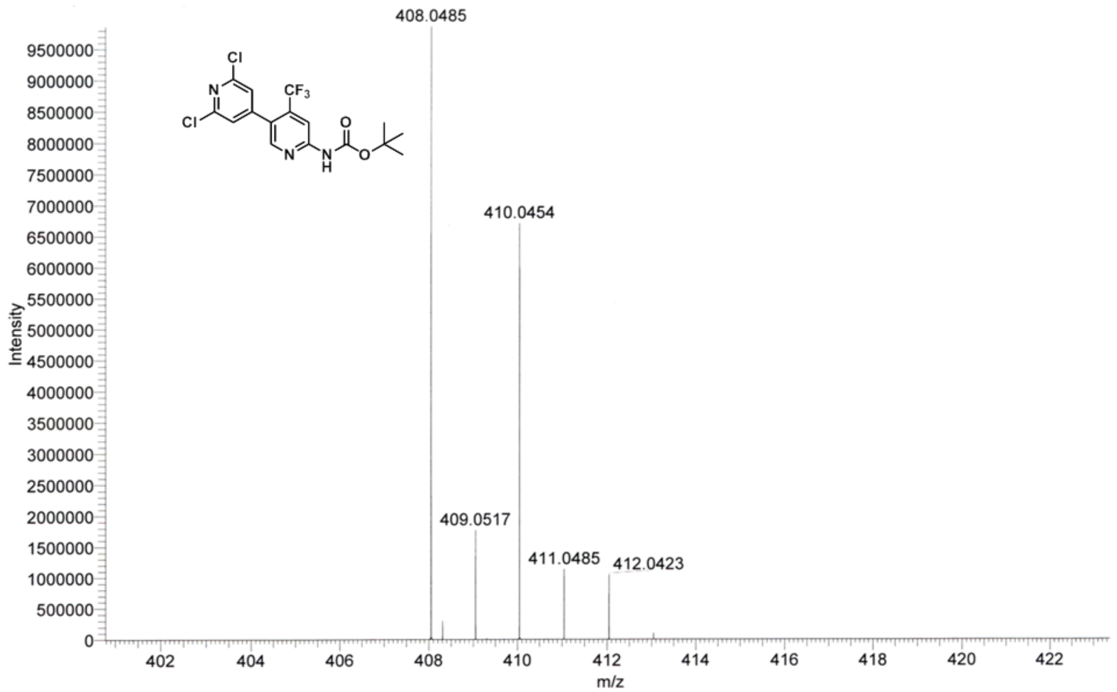

2',6'-Dimorpholino-4-(trifluoromethyl)-(3,4'-bipyridin)-6-amine (**MTD147**):

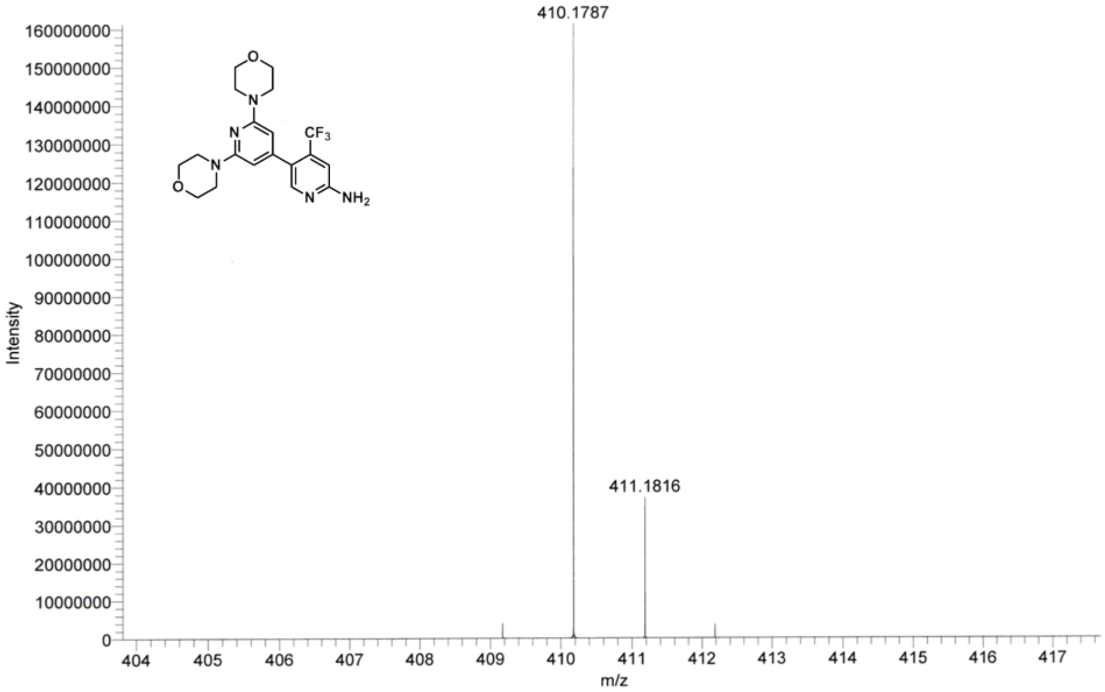

5-(4,6-Dimorpholinopyrimidin-2-yl)-4-(trifluoromethyl)pyridin-2-amine (**BKM120-R1**):

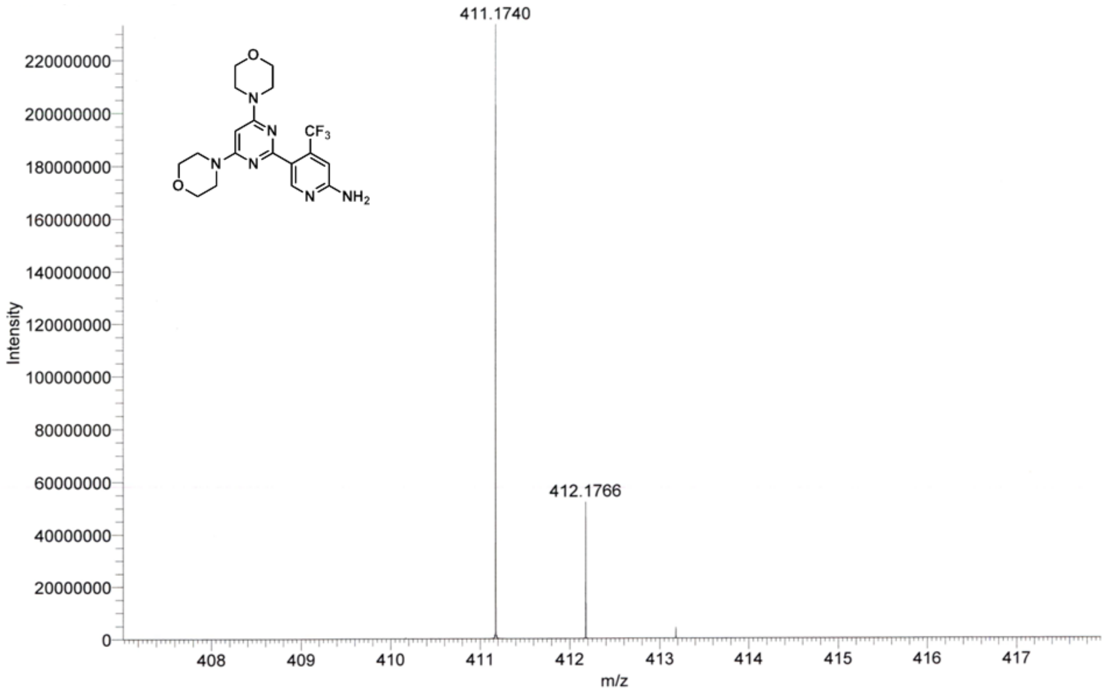

4-(4,6-Dichloropyrimidin-2-yl)morpholine (**10**):

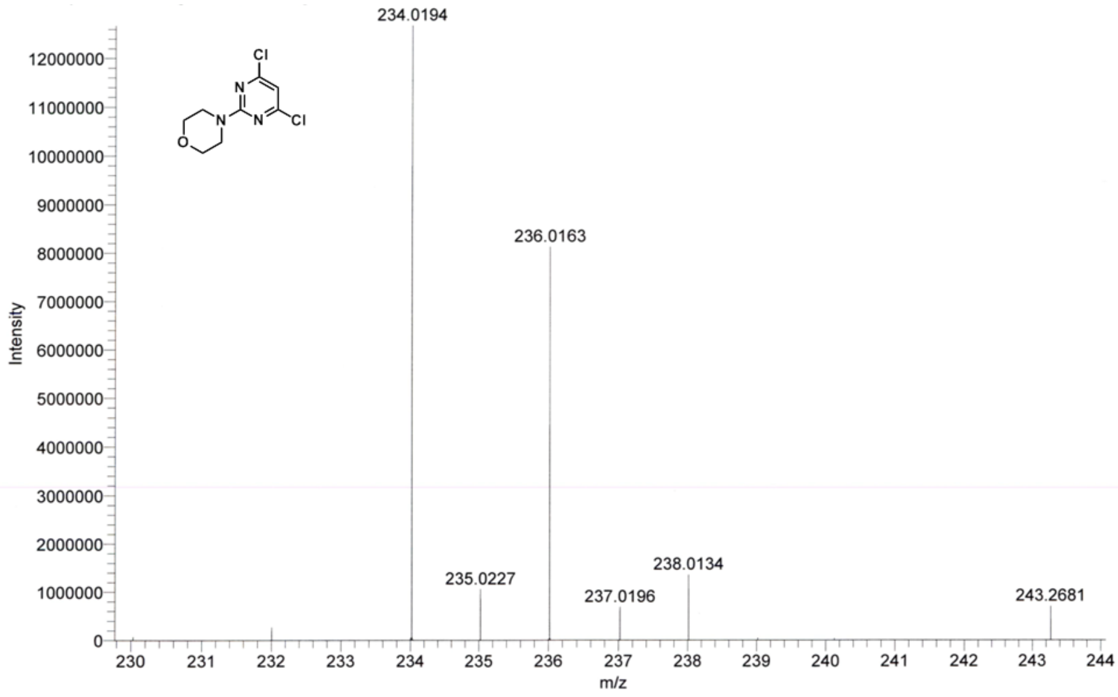

4-(2,6-Dichloropyrimidin-4-yl)morpholine (**11**):

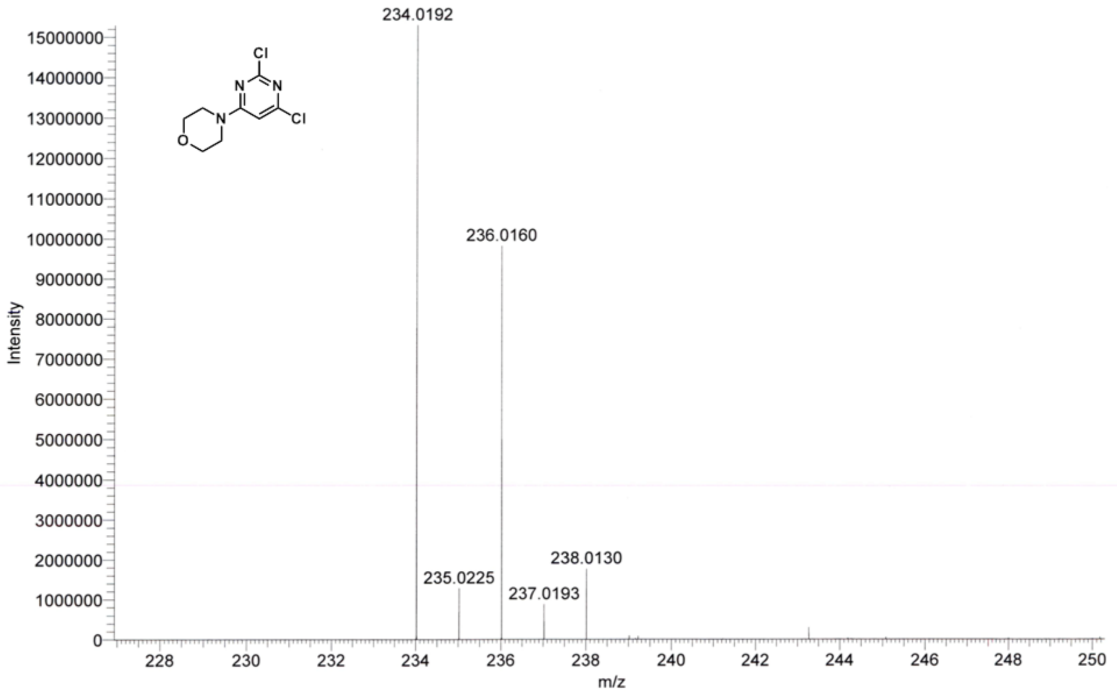

6-(6-Chloro-2-morpholinopyrimidin-4-yl)-2-oxa-6-azaspiro[3.3]heptane (**12**):

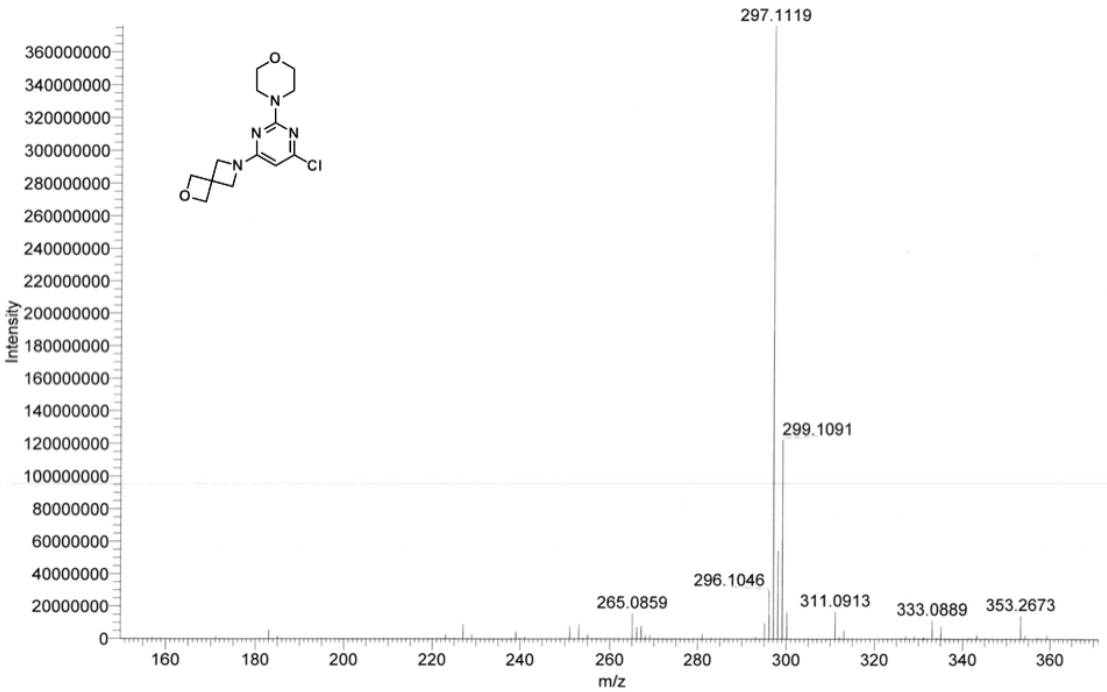

(1-(6-(6-Amino-4-(trifluoromethyl)pyridin-3-yl)-2-morpholinopyrimidin-4-yl)-3-(chloromethyl)azetidin-3-yl)methanol (**PIKiN2-R1**):

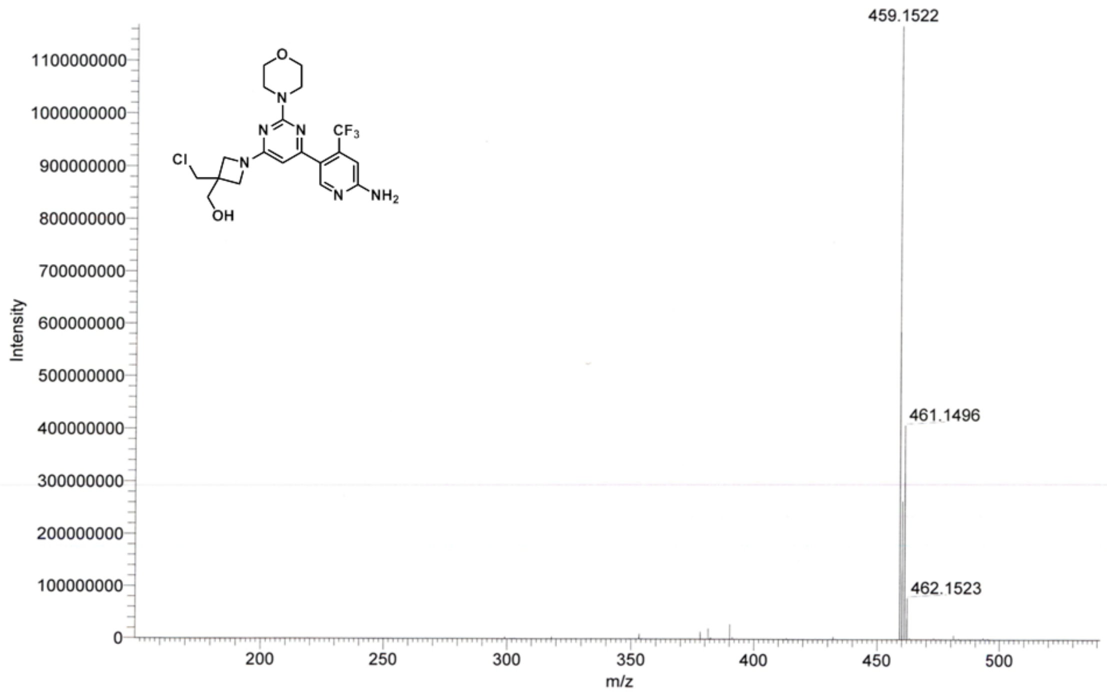

6-(4-Chloro-6-morpholinopyrimidin-2-yl)-2-oxa-6-azaspiro[3.3]heptane (**13**):

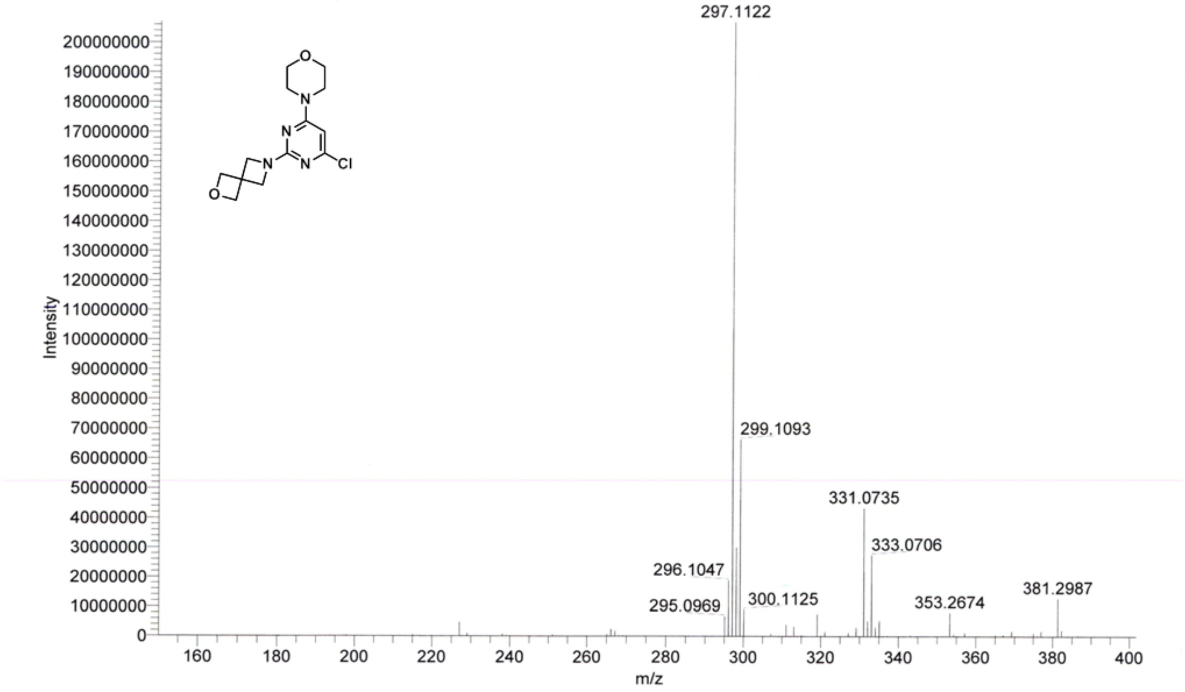

(1-(4-(6-Amino-4-(trifluoromethyl)pyridin-3-yl)-6-morpholinopyrimidin-2-yl)-3-(chloromethyl)azetidin-3-yl)methanol (**PIKiN2**):

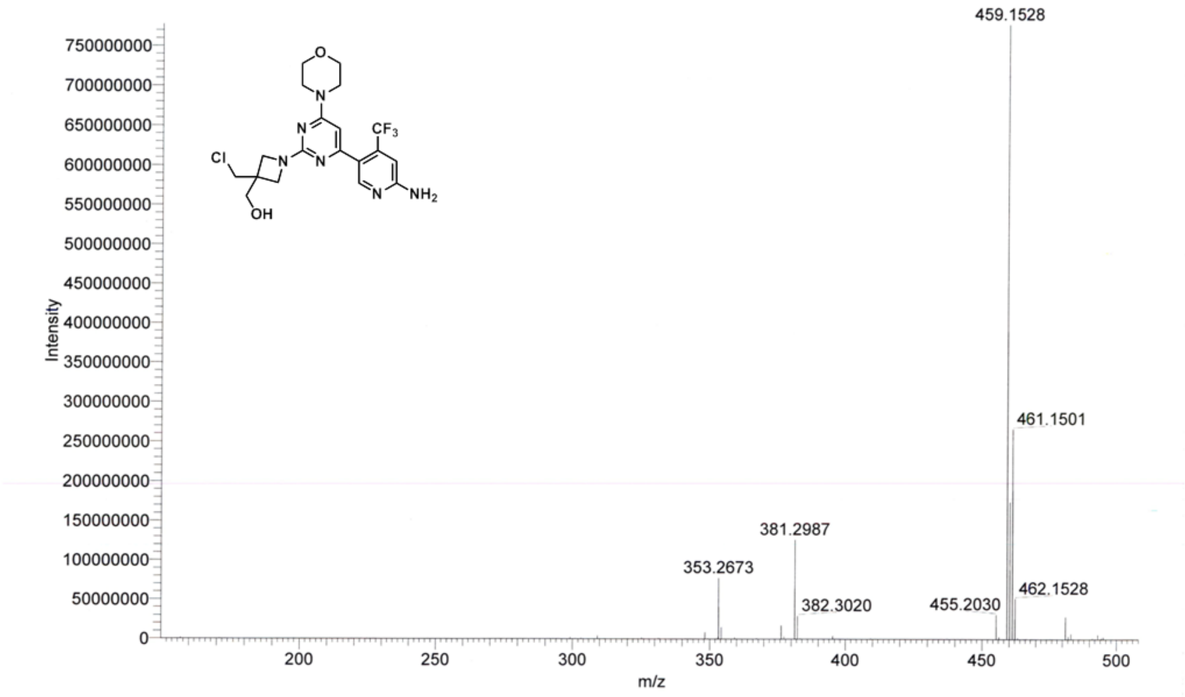

4-(4-Chloro-6-(piperidin-1-yl)pyrimidin-2-yl)morpholine (**14**):

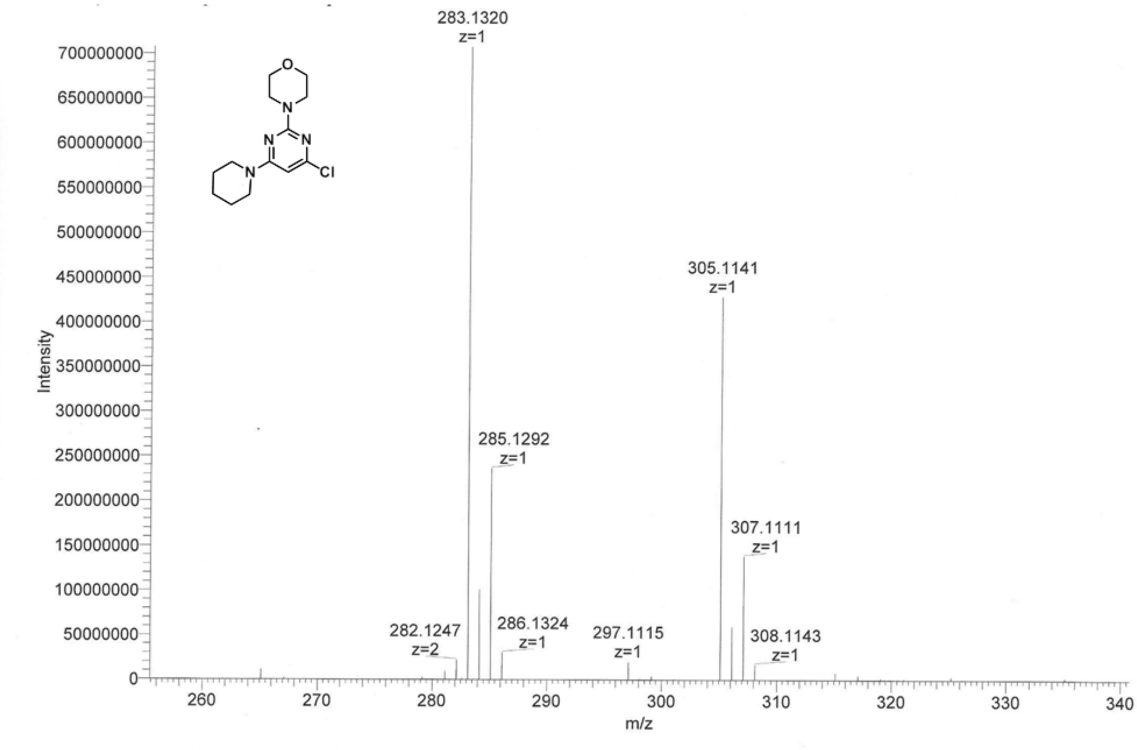

5-(2-Morpholino-6-(piperidin-1-yl)pyrimidin-4-yl)-4-(trifluoromethyl)pyridin-2-amine (**PIKiN1-R1**):

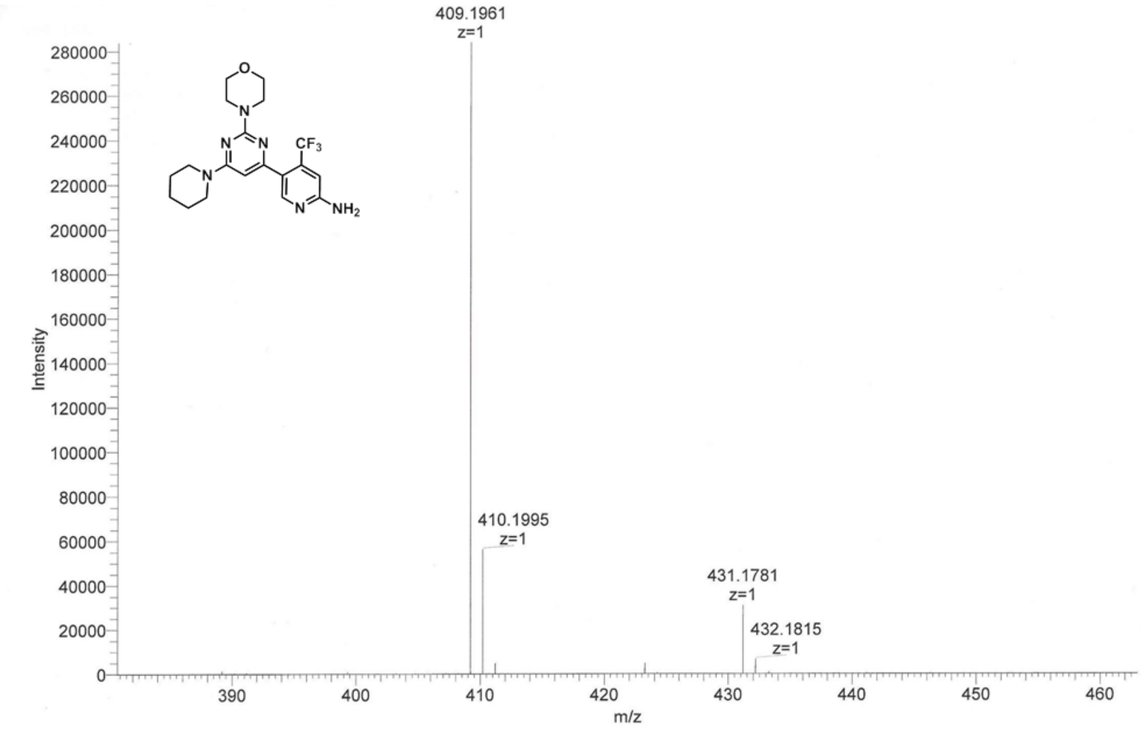

4-(6-Chloro-2-(piperidin-1-yl)pyrimidin-4-yl)morpholine (**15**):

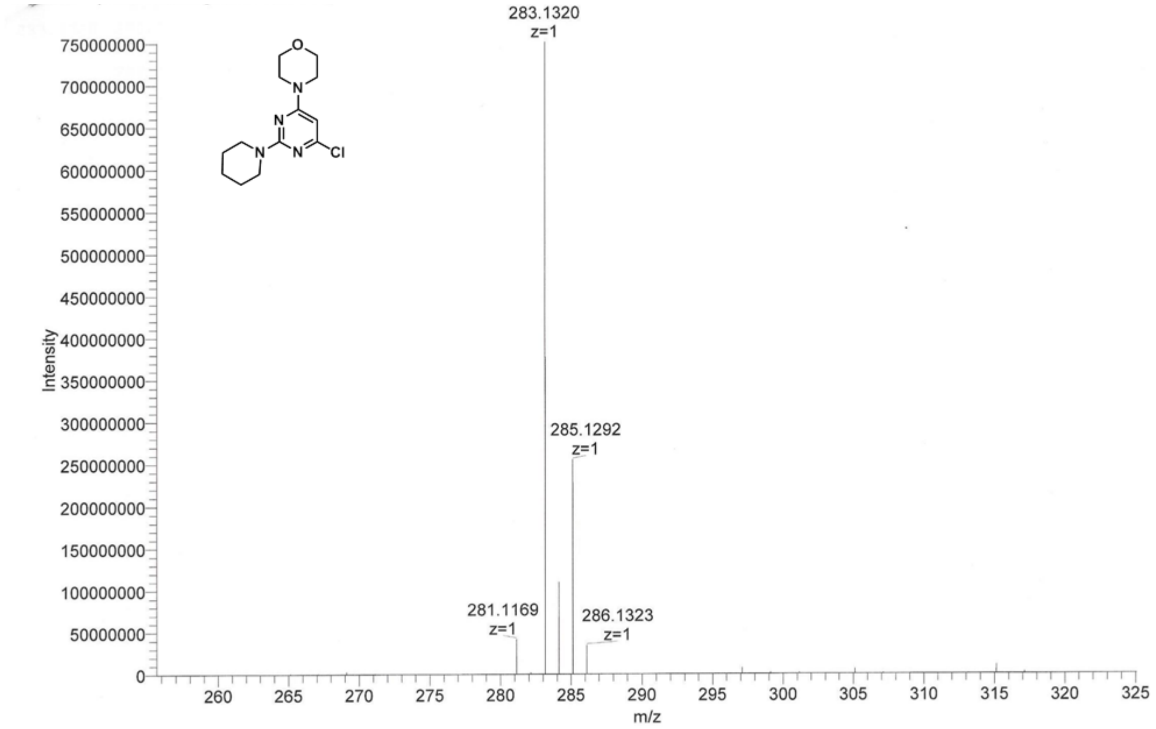

5-(6-Morpholino-2-(piperidin-1-yl)pyrimidin-4-yl)-4-(trifluoromethyl)pyridin-2-amine (**PIKiN1**):

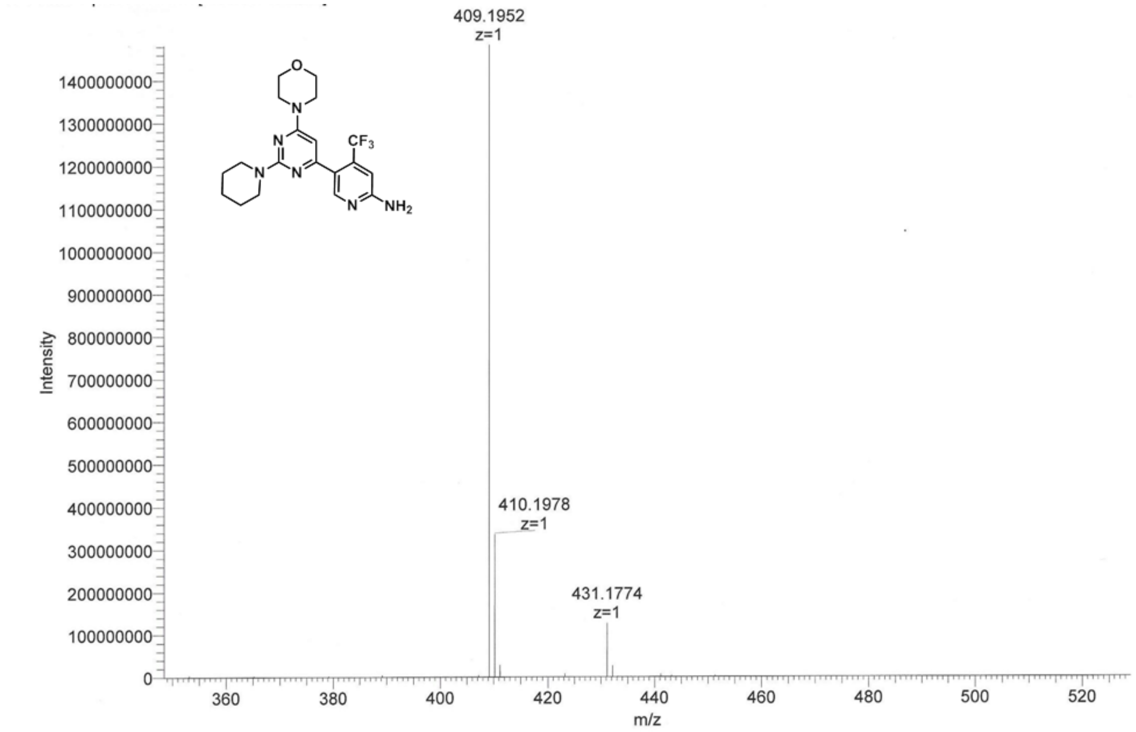

4-(4-Chloro-6-(pyrrolidin-1-yl)pyrimidin-2-yl)morpholine (**16**):

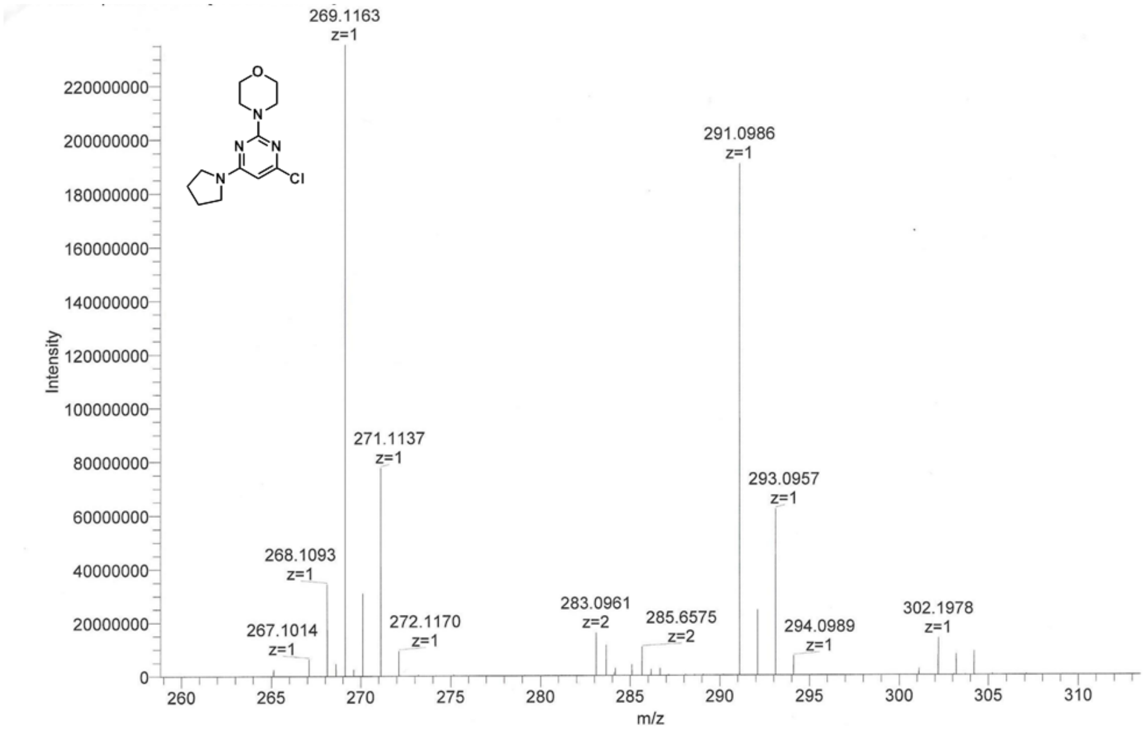

5-(2-Morpholino-6-(pyrrolidin-1-yl)pyrimidin-4-yl)-4-(trifluoromethyl)pyridin-2-amine (**MTD265**):

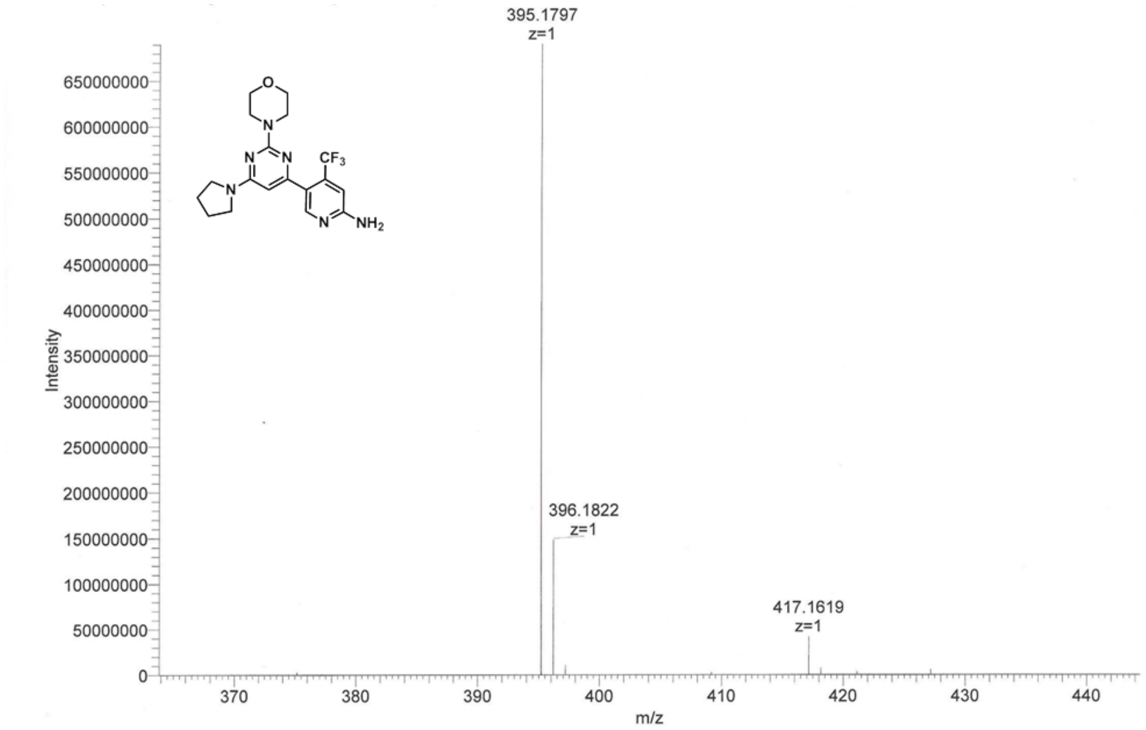

4-(6-Chloro-2-(pyrrolidin-1-yl)pyrimidin-4-yl)morpholine (**17**):

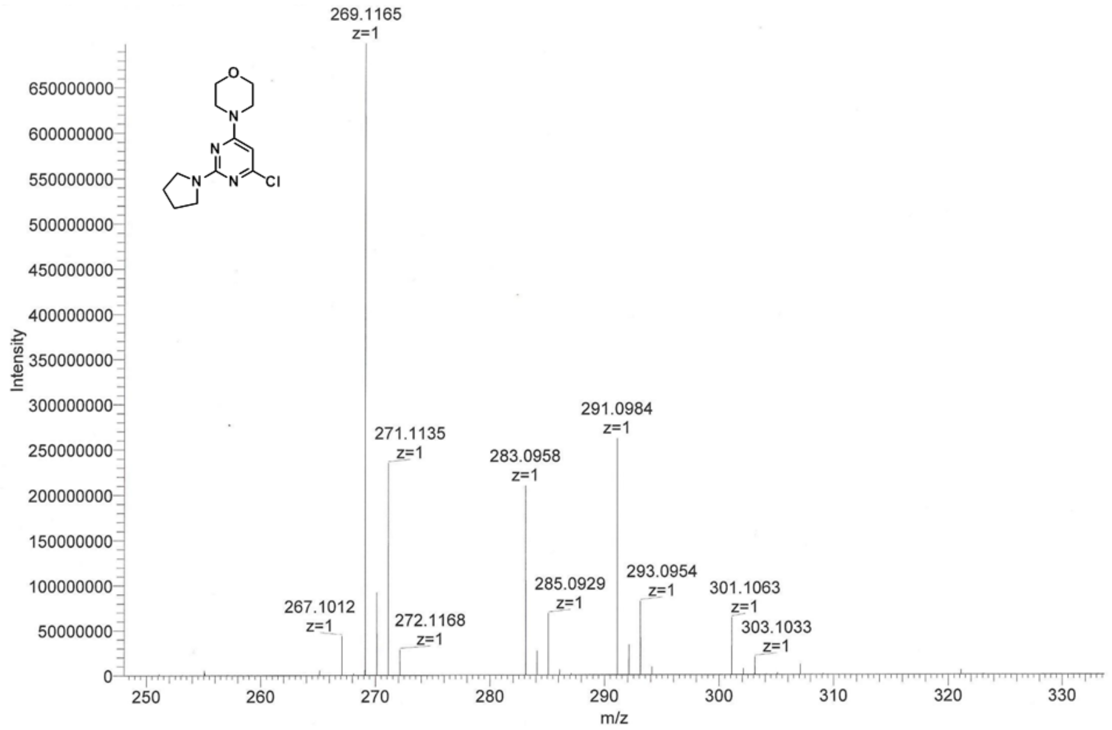

5-(6-Morpholino-2-(pyrrolidin-1-yl)pyrimidin-4-yl)-4-(trifluoromethyl)pyridin-2-amine (**MTD265-R1**):

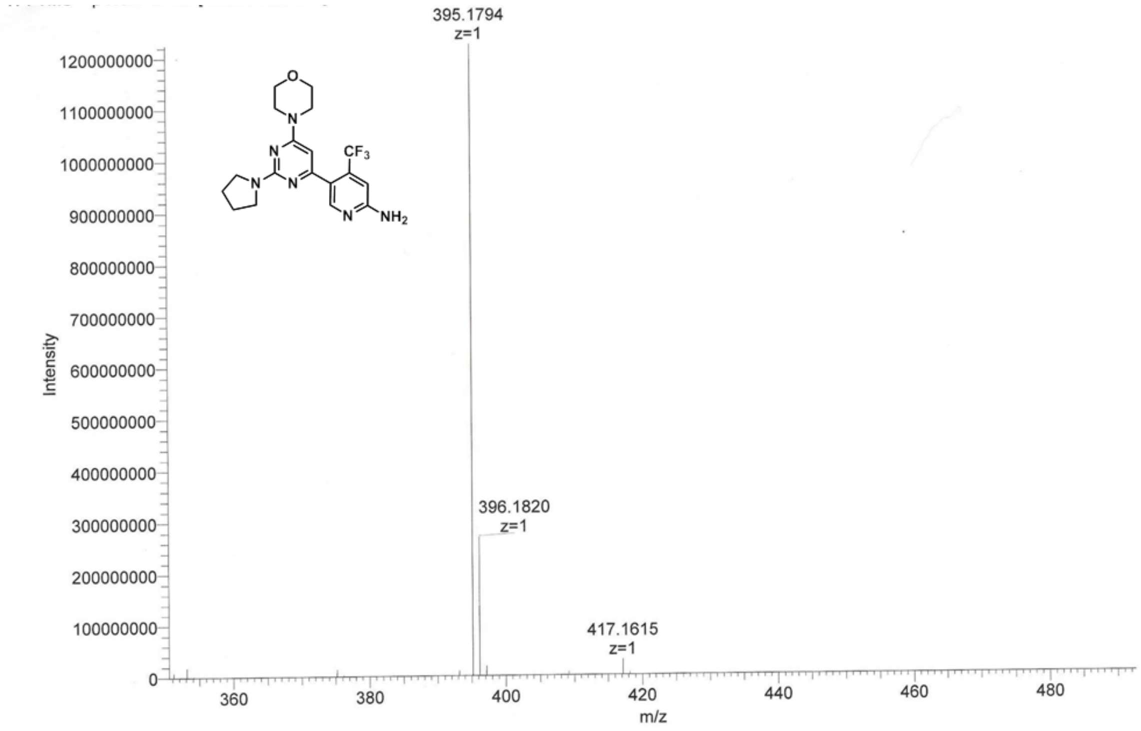

5-(4,6-Dimorpholino-1,3,5-triazin-2-yl)-4-(trifluoromethyl)pyridin-2-amine (**PQR309**):

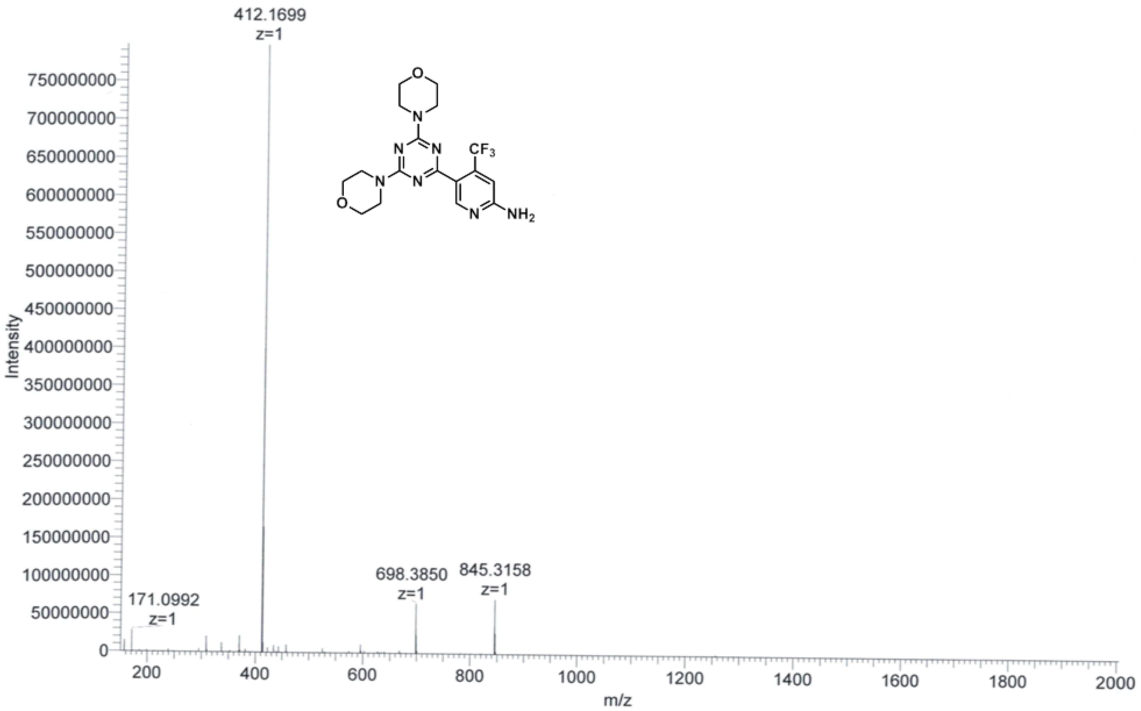

6-(4-Chloro-6-morpholino-1,3,5-triazin-2-yl)-2-oxa-6-azaspiro[3.3]heptane (**21**):

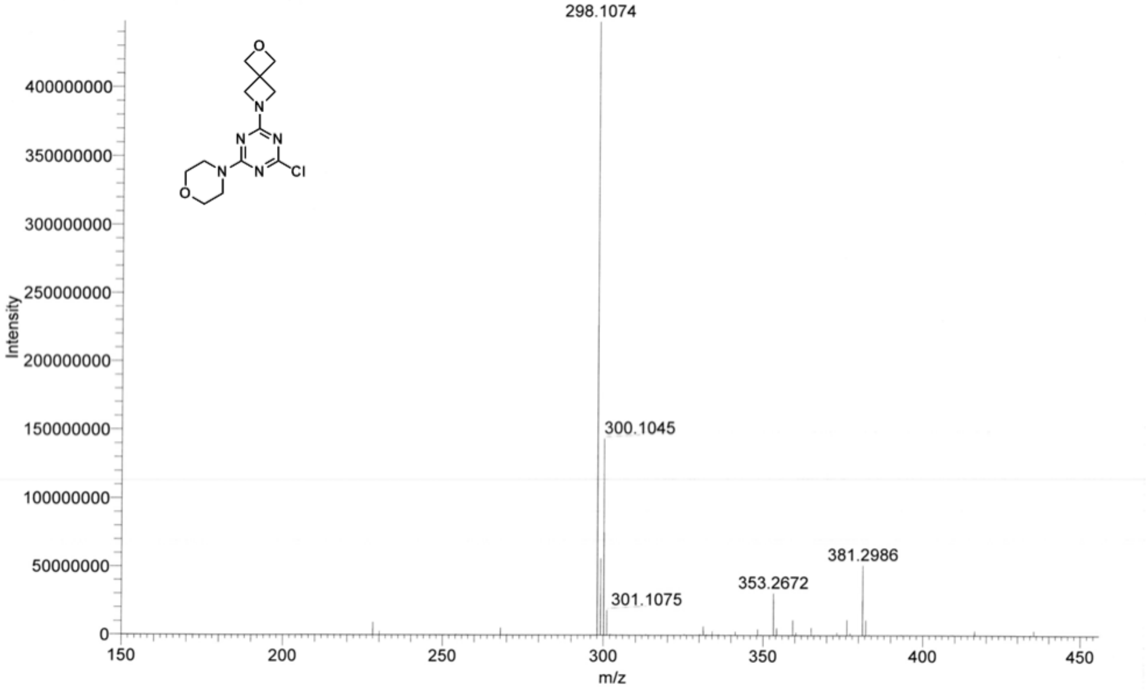

(1-(4-(6-Amino-4-(trifluoromethyl)pyridin-3-yl)-6-morpholino-1,3,5-triazin-2-yl)-3-(chloromethyl)azetidin-3-yl)methanol (**PIKiN3**):

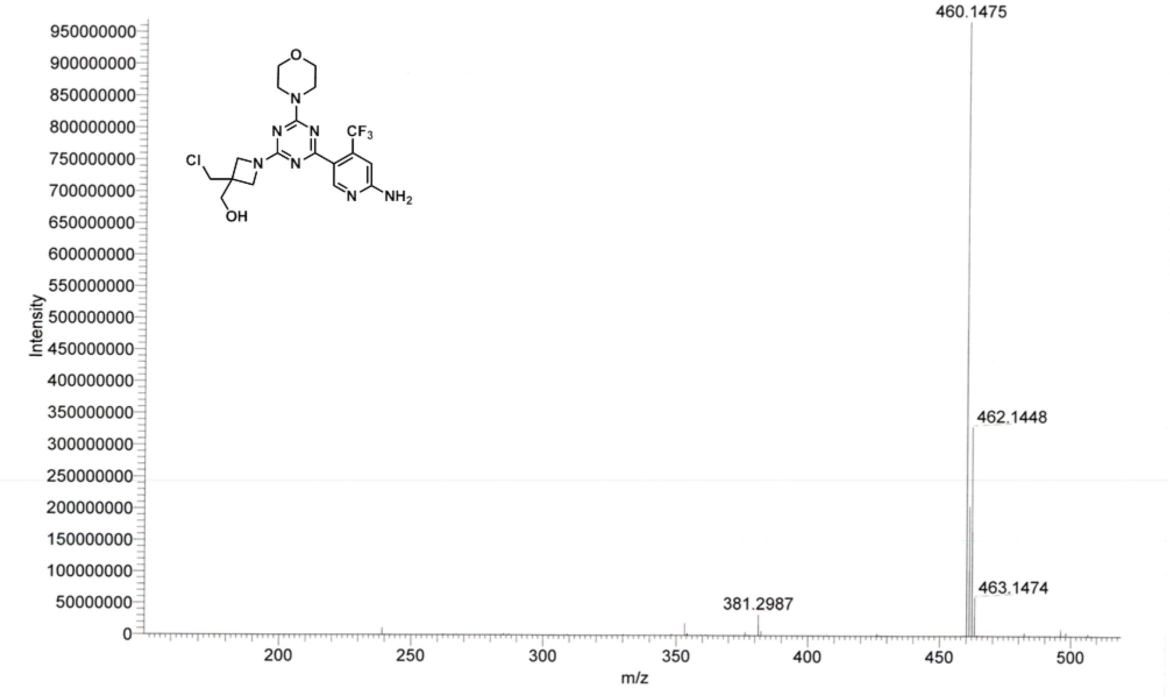

HPLC Chromatograms

2',6'-Dimorpholino-4-(trifluoromethyl)-(3,4'-bipyridin)-6-amine (**MTD147**):

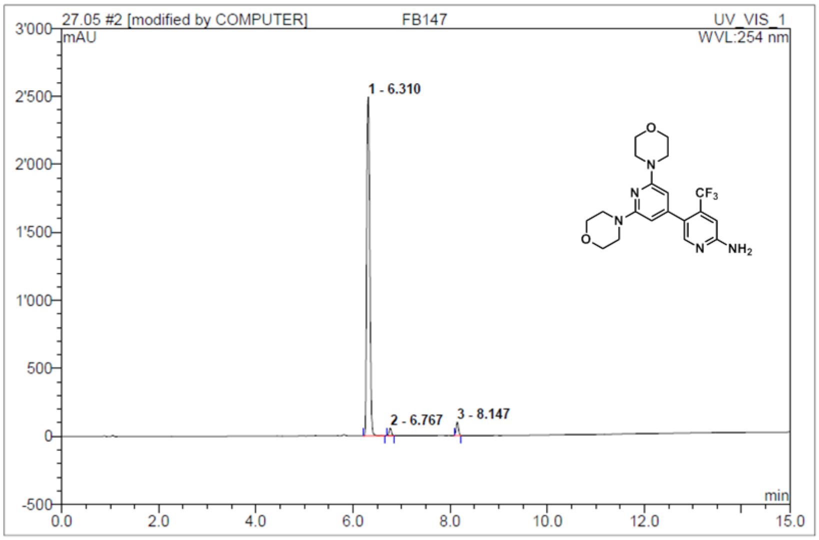

No.	Ret.Time min	Peak Name	Height mAU	Area mAU*min	Rel.Area %	Type
1	6.31	n.a.	2490.644	181.152	95.43	BMB*
2	6.77	n.a.	54.753	3.098	1.63	BMB*
3	8.15	n.a.	98.666	5.568	2.93	BMB*
Total:			2644.063	189.819	100.00	#REF!

5-(2,6-Dimorpholinopyrimidin-4-yl)-4-(trifluoromethyl)pyridin-2-amine (**BKM120**):

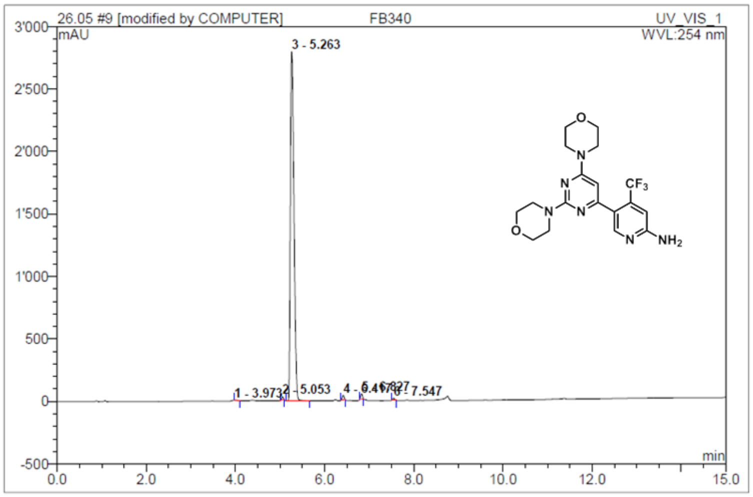

No.	Ret.Time min	Peak Name	Height mAU	Area mAU*min	Rel.Area %	Type
1	3.97	n.a.	0.069	0.006	0.00	BMB*
2	5.05	n.a.	26.156	1.106	0.42	BMB*
3	5.26	n.a.	2790.656	259.100	97.85	BMB*
4	6.42	n.a.	36.006	1.743	0.66	BMB*
5	6.83	n.a.	42.545	1.966	0.74	BMB*
6	7.55	n.a.	17.691	0.885	0.33	BMB*
Total:			2913.122	264.806	100.00	#REF!

5-(4,6-Dimorpholinopyrimidin-2-yl)-4-(trifluoromethyl)pyridin-2-amine (**BKM120-R1**):

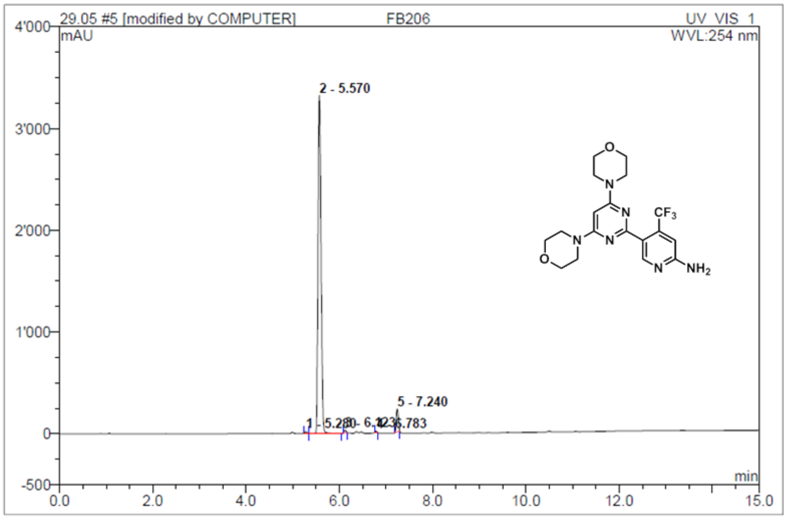

No.	Ret.Time min	Peak Name	Height mAU	Area mAU*min	Rel.Area %	Type
1	5.28	n.a.	17.867	0.881	0.33	BMb*
2	5.57	n.a.	3323.391	253.179	94.66	bMB*
3	6.12	n.a.	26.224	1.164	0.44	BMB*
4	6.78	n.a.	13.531	0.557	0.21	BMB*
5	7.24	n.a.	224.868	11.670	4.36	BMB*
Total:			3605.882	267.451	100.00	#REF!

(1-(6-(6-Amino-4-(trifluoromethyl)pyridin-3-yl)-2-morpholinopyrimidin-4-yl)-3-(chloromethyl)azetidin-3-yl)methanol (**PIKiN2-R1**):

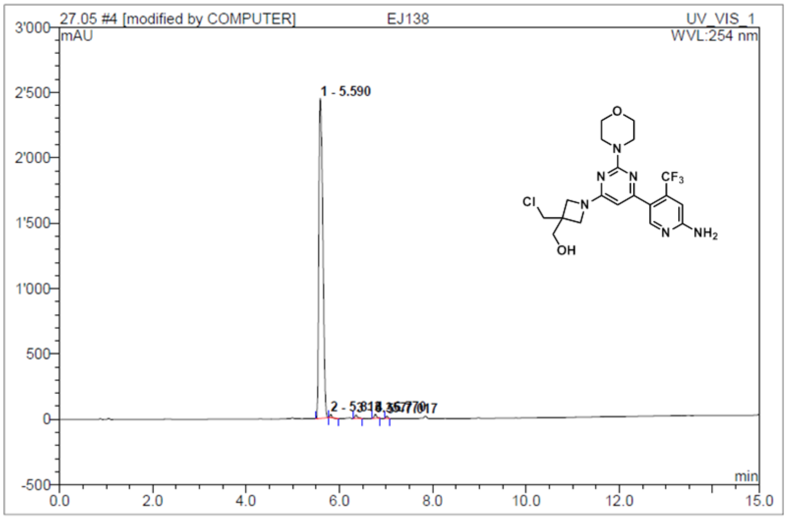

No.	Ret.Time min	Peak Name	Height mAU	Area mAU*min	Rel.Area %	Type
1	5.59	n.a.	2445.264	243.909	97.90	BMB*
2	5.81	n.a.	24.098	0.970	0.39	BMB*
3	6.36	n.a.	25.566	1.664	0.67	BMB*
4	6.77	n.a.	30.355	1.780	0.71	BMB*
5	7.02	n.a.	16.530	0.814	0.33	BMB*
Total:			2541.813	249.137	100.00	#REF!

(1-(4-(6-Amino-4-(trifluoromethyl)pyridin-3-yl)-6-morpholinopyrimidin-2-yl)-3-(chloromethyl)azetidin-3-yl)methanol (**PIKiN2**):

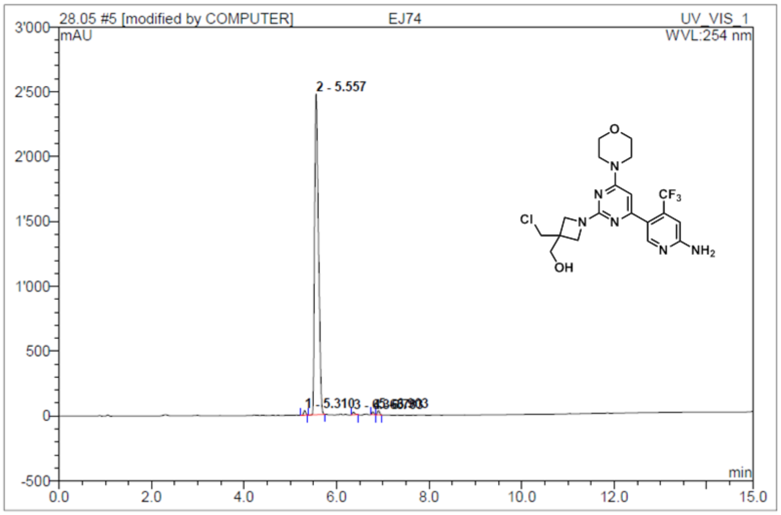

No.	Ret.Time min	Peak Name	Height mAU	Area mAU*min	Rel.Area %	Type
1	5.31	n.a.	35.722	1.949	0.80	BMB*
2	5.56	n.a.	2472.340	238.253	97.51	BMB*
3	6.36	n.a.	19.736	1.274	0.52	BMB*
4	6.78	n.a.	22.872	1.188	0.49	BM *
5	6.90	n.a.	32.363	1.680	0.69	MB*
Total:			2583.032	244.344	100.00	#REF!

5-(2-Morpholino-6-(piperidin-1-yl)pyrimidin-4-yl)-4-(trifluoromethyl)pyridin-2-amine (**PIKiN1-R1**):

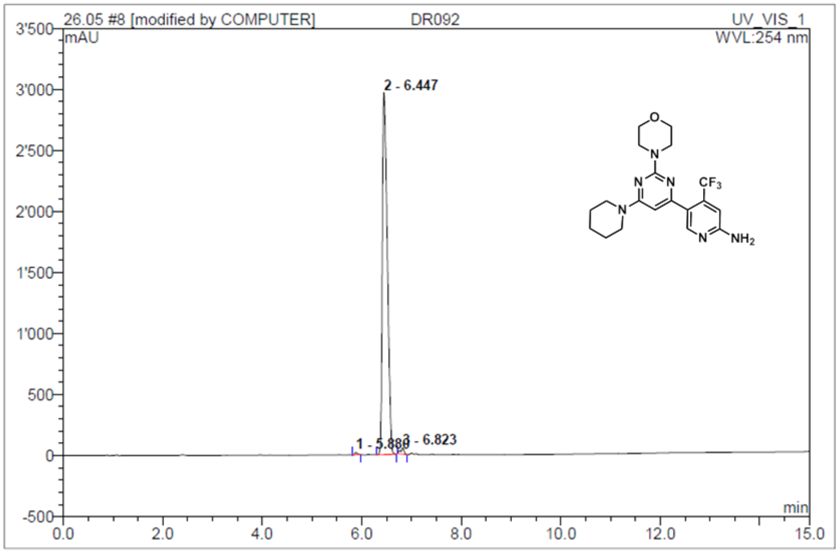

No.	Ret.Time min	Peak Name	Height mAU	Area mAU*min	Rel.Area %	Type
1	5.88	n.a.	19.548	1.185	0.34	BMB*
2	6.45	n.a.	2964.930	338.492	98.43	BMB*
3	6.82	n.a.	49.035	4.225	1.23	BMB*
Total:			3033.514	343.901	100.00	#REF!

5-(6-Morpholino-2-(piperidin-1-yl)pyrimidin-4-yl)-4-(trifluoromethyl)pyridin-2-amine (**PIKiN1**):

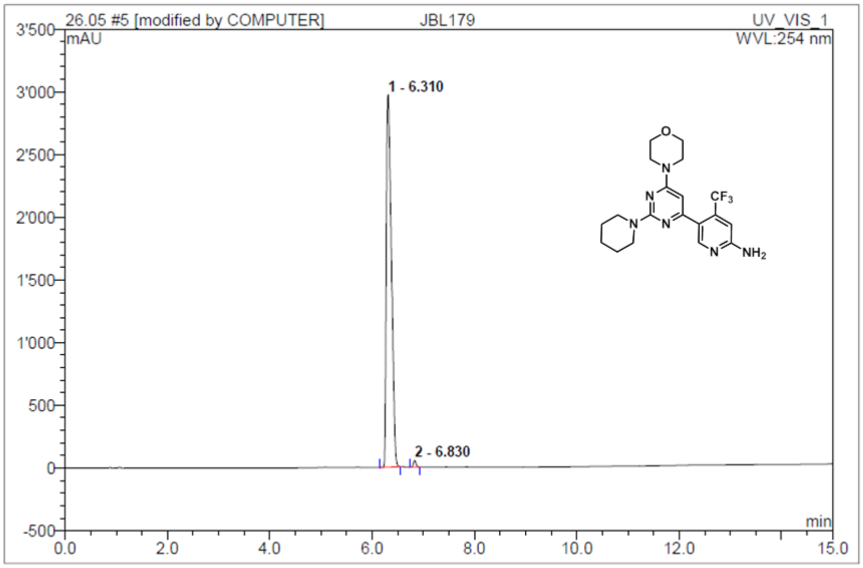

No.	Ret.Time min	Peak Name	Height mAU	Area mAU*min	Rel.Area %	Type
1	6.31	n.a.	2971.900	342.179	99.10	BMB
2	6.83	n.a.	52.911	3.101	0.90	BMB*
Total:			3024.811	345.280	100.00	#REF!

5-(2-Morpholino-6-(pyrrolidin-1-yl)pyrimidin-4-yl)-4-(trifluoromethyl)pyridin-2-amine (**MTD265**):

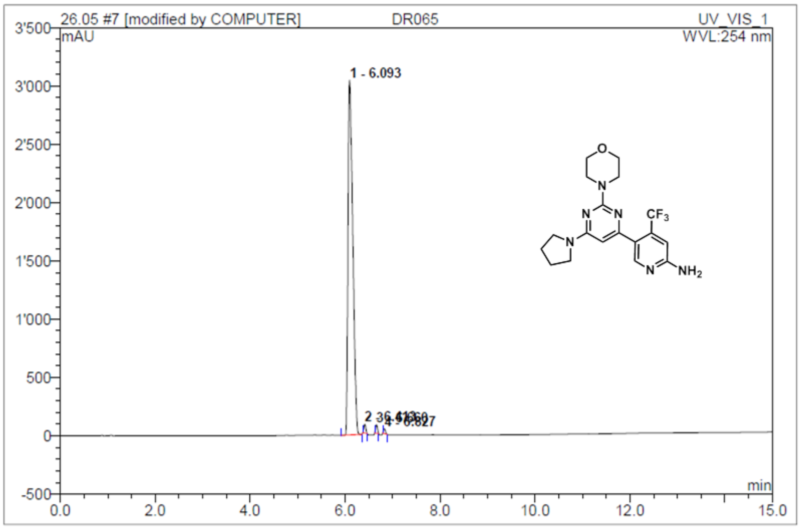

No.	Ret.Time min	Peak Name	Height mAU	Area mAU*min	Rel.Area %	Type
1	6.09	n.a.	3040.728	354.473	97.57	BMB*
2	6.41	n.a.	76.051	3.673	1.01	BMB*
3	6.66	n.a.	71.841	3.298	0.91	BMB*
4	6.83	n.a.	40.106	1.854	0.51	BMB*
Total:			3228.725	363.297	100.00	#REF!

5-(6-Morpholino-2-(pyrrolidin-1-yl)pyrimidin-4-yl)-4-(trifluoromethyl)pyridin-2-amine (**MTD265-R1**):

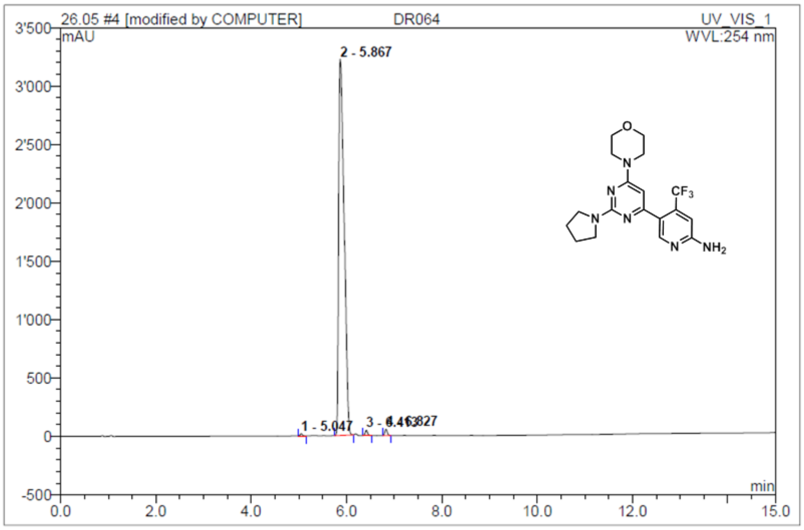

No.	Ret.Time min	Peak Name	Height mAU	Area mAU*min	Rel.Area %	Type
1	5.05	n.a.	18.662	0.985	0.23	BMB*
2	5.87	n.a.	3221.043	429.045	98.42	BMB
3	6.41	n.a.	45.756	2.759	0.63	BMB*
4	6.83	n.a.	53.918	3.157	0.72	BMB*
Total:			3339.380	435.946	100.00	#REF!

5-(4,6-Dimorpholino-1,3,5-triazin-2-yl)-4-(trifluoromethyl)pyridin-2-amine (**PQR309**):

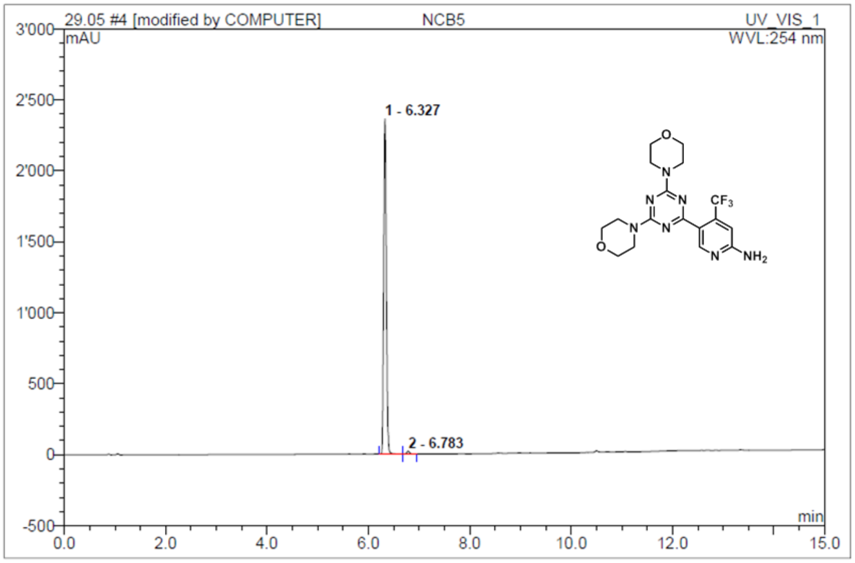

No.	Ret.Time min	Peak Name	Height mAU	Area mAU*min	Rel.Area %	Type
1	6.33	n.a.	2360.587	141.480	99.15	BM
2	6.78	n.a.	20.147	1.220	0.85	MB
Total:			2380.734	142.700	100.00	#REF!

(1-(4-(6-Amino-4-(trifluoromethyl)pyridin-3-yl)-6-morpholino-1,3,5-triazin-2-yl)-3-(chloromethyl)azetidin-3-yl)methanol (**PIKiN3**):

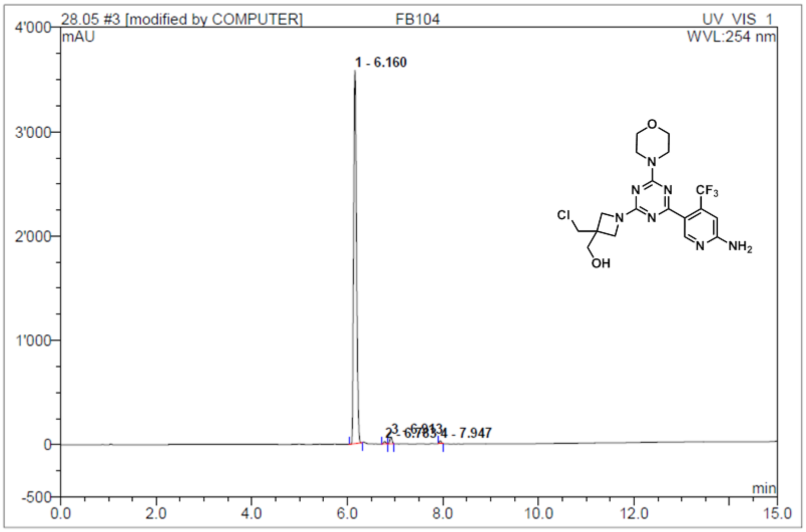

No.	Ret.Time min	Peak Name	Height mAU	Area mAU*min	Rel.Area %	Type
1	6.16	n.a.	3581.006	249.938	97.66	BMB*
2	6.78	n.a.	23.270	1.281	0.50	BM *
3	6.91	n.a.	62.838	3.248	1.27	MB*
4	7.95	n.a.	28.349	1.464	0.57	BMB*
Total:			3695.463	255.931	100.00	#REF!

REFERENCES

1. Peng, W., Tu, Z. C., Long, Z. J., Liu, Q. & Lu, G. Discovery of 2-(2-aminopyrimidin-5-yl)-4-morpholino-N-(pyridin-3-yl)quinazolin-7-amines as novel PI3K/mTOR inhibitors and anticancer agents. *Eur J Med Chem* **108**, 644-654 (2016).
2. Burger, M. T. et al. Identification of NVP-BKM120 as a Potent, Selective, Orally Bioavailable Class I PI3 Kinase Inhibitor for Treating Cancer. *ACS Med Chem Lett* **2**, 774-779 (2011).
3. Kurteva, V. B. & Afonso, C. A. M. Solvent-free synthesis of melamines under microwave irradiation. *Green Chem.* **6**, 183-187 (2004).
4. Chen, Z. et al. Stereoselective synthesis of an active metabolite of the potent PI3 kinase inhibitor PKI-179. *J Org Chem* **75**, 1643-1651 (2010).
